# Supplementary material for: Design, Synthesis, and Development of pyrazolo[1,5-a]pyrimidine Derivatives as a Novel Series of Selective PI3Kδ Inhibitors: Part I—Indole Derivatives
Source: Pharmaceuticals (Basel). 2022 Jul 30;15(8):949. doi: 10.3390/ph15080949 (PMC9412374; doi:10.3390/ph15080949)
Supplement: Supplementary file 1 [file pharmaceuticals-15-00949-s001.zip › Supplementary data - Part_1 new.pdf]

Supplementary data

for

Design, synthesis, and development of pyrazolo[1,5-*a*]pyrimidine derivatives as a novel series of selective PI3K*δ* inhibitors. Part I – Indole derivatives.

Mariola Stypik[1,2*], Marcin Zagozda[1], Stanisław Michałek[1,2], Barbara Dymek[1], Daria Zdżalik-Bielecka[1], Maciej Dziachan[1], Nina Orłowska[1,2], Paweł Gunerka[1], Paweł Turowski[1], Joanna Hucz-Kalitowska[1], Aleksandra Stańczak[1], Paulina Stańczak[1], Krzysztof Mulewski[1], Damian Smuga[1], Filip Stefaniak[1], Lidia Gurba-Bryśkiewicz[1], Arkadiusz Leniak[1], Zbigniew Ochal[2], Mateusz Mach[1], Karolina Dzwonek[1], Monika Lamparska-Przybysz[1], Krzysztof Dubiel[1], Maciej Wieczorek[1]

[1] Celon Pharma S.A. ul. Marymoncka 15, 05-152 Kazuń Nowy, Poland
[2] Faculty of Chemistry, Warsaw University of Technology, ul. Noakowskiego 3, 00-664 Warsaw, Poland
*****corresponding author: mariola.stypik@celonpharma.com

^{1}H NMR, ^{13}C NMR and HRMS supplementary figures of the final compounds **5-13**, **23-45**, **49-51** and **53-55**

INDEX

Compound 5 ^{1}H NMR 4

Compound 6 ^{1}H NMR 4

Compound 7 ^{1}H NMR 5

Compound 9 ^{1}H NMR 5

Compound 10 ^{1}H NMR 6

Compound 11 ^{1}H NMR 6

Compound 12 ^{1}H NMR 7

Compound 13 ^{1}H NMR 7

Compound 23 ^{1}H NMR and ^{13}C NMR 8

Compound 23 HRMS 9

Compound 24 ^{1}H NMR 10

Compound 24 HRMS 11

Compound 25 ^{1}H NMR and ^{13}C NMR 12

Compound 25 HRMS 13

Compound 26 ^{1}H NMR and ^{13}C NMR 14

Compound 26 HRMS 15

Compound 27 ^{1}H NMR and ^{13}C NMR 16

Compound 27 HRMS 17

Compound 28 ^{1}H NMR and ^{13}C NMR 18

Compound 28 HRMS 19

Compound 29 ^{1}H NMR and ^{13}C NMR 20

Compound 29 HRMS 21

Compound 30 ^{1}H NMR and ^{13}C NMR 22

Compound 30 HRMS 23

Compound 31 ^{1}H NMR and ^{13}C NMR 24

Compound 31 HRMS 25

Compound 32 ^{1}H NMR and ^{13}C NMR 26

Compound 32 HRMS 27

Compound 33 ^{1}H NMR and ^{13}C NMR 28

Compound 33 HRMS 29

Compound 34 ^{1}H NMR and ^{13}C NMR 30

Compound 34 HRMS 31

Compound 35 ^{1}H NMR and ^{13}C NMR 32

Compound 35 HRMS 33

Compound 36 ^{1}H NMR and ^{13}C NMR 34

Compound 36 HRMS 35
Compound 37 ^{1}H NMR and ^{13}C NMR 36
Compound 37 HRMS 37
Compound 38 ^{1}H NMR and ^{13}C NMR 38
Compound 38 HRMS 39
Compound 39 ^{1}H NMR and ^{13}C NMR 40
Compound 39 HRMS 41
Compound 40 ^{1}H NMR and ^{13}C NMR 42
Compound 40 HRMS 43
Compound 41 ^{1}H NMR and ^{13}C NMR 44
Compound 41 HRMS 45
Compound 42 ^{1}H NMR and ^{13}C NMR 46
Compound 42 HRMS 47
Compound 43 ^{1}H NMR and ^{13}C NMR 48
Compound 43 HRMS 49
Compound 44 ^{1}H NMR and ^{13}C NMR 50
Compound 44 HRMS 51
Compound 49 ^{1}H NMR and ^{13}C NMR 52
Compound 49 HRMS 53
Compound 50 ^{1}H NMR and ^{13}C NMR 54
Compound 50 HRMS 55
Compound 51 ^{1}H NMR and ^{13}C NMR 56
Compound 51 HRMS 57
Compound 53 ^{1}H NMR and ^{13}C NMR 58
Compound 53 HRMS 59
Compound 54 ^{1}H NMR and ^{13}C NMR 60
Compound 54 HRMS 61
Compound 55 ^{1}H NMR and ^{13}C NMR 62
Compound 55 HRMS 63

Compound 5

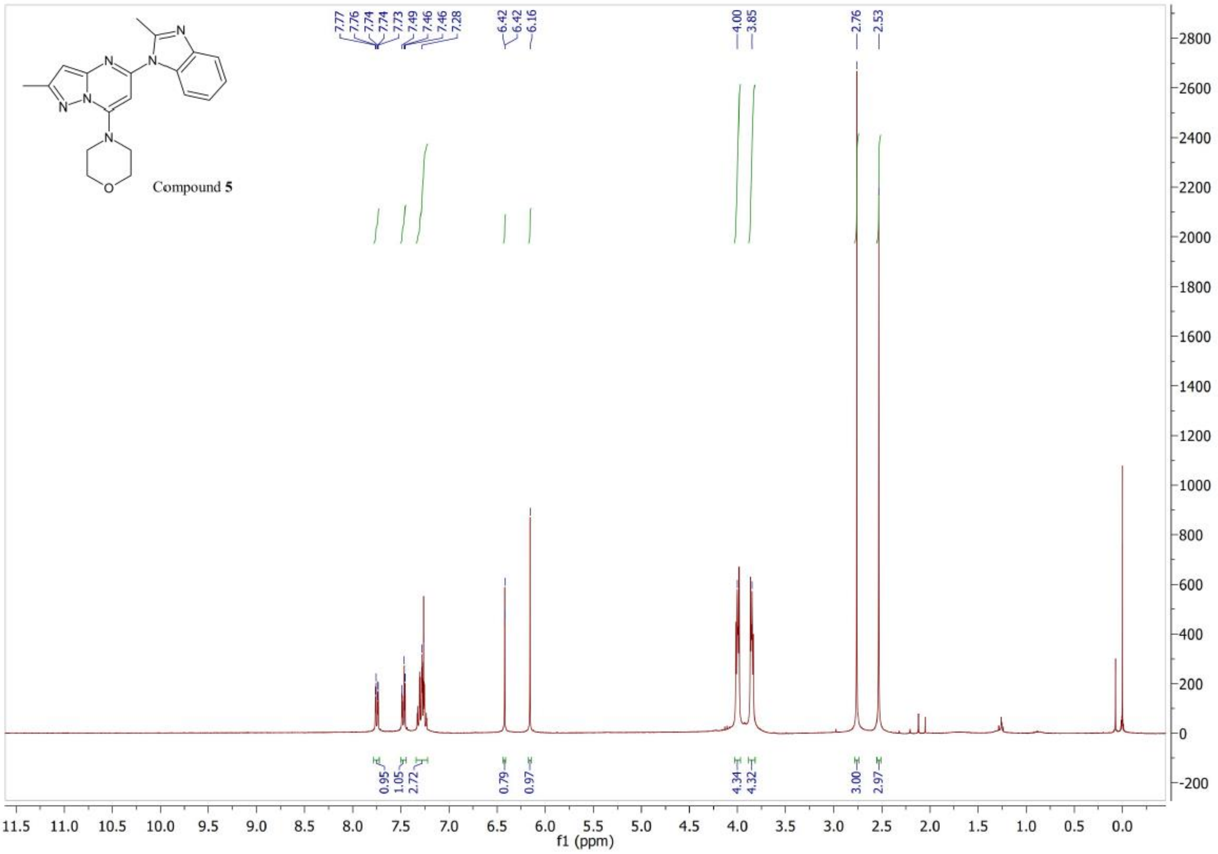

Compound 6

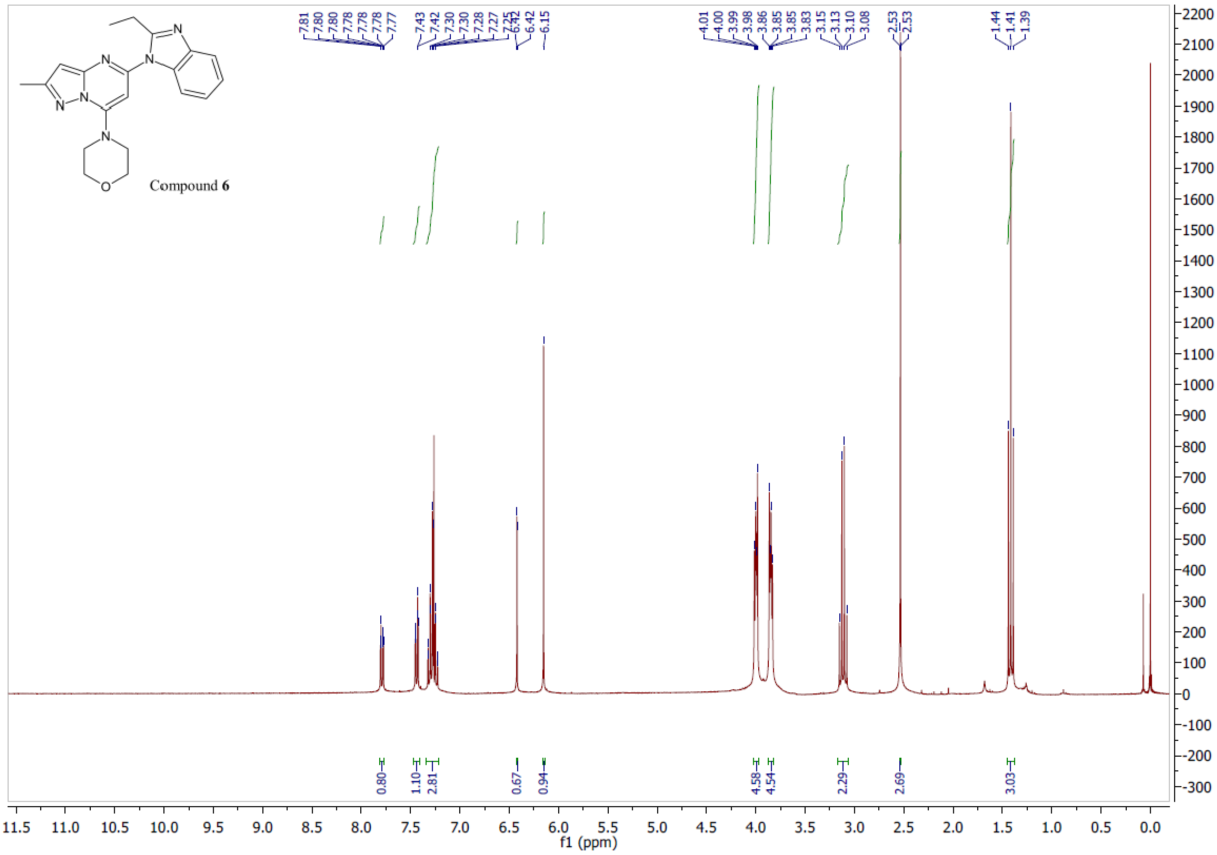

Compound 7

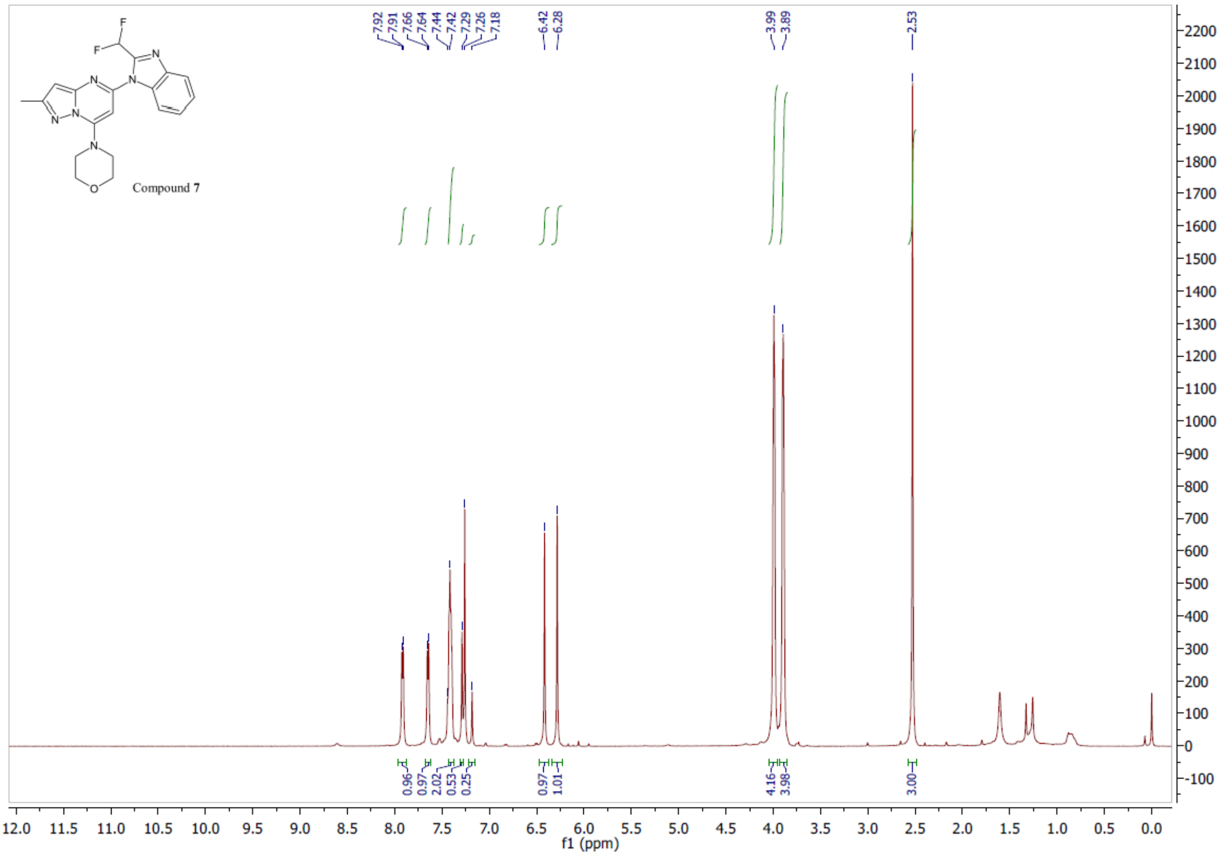

Compound 9

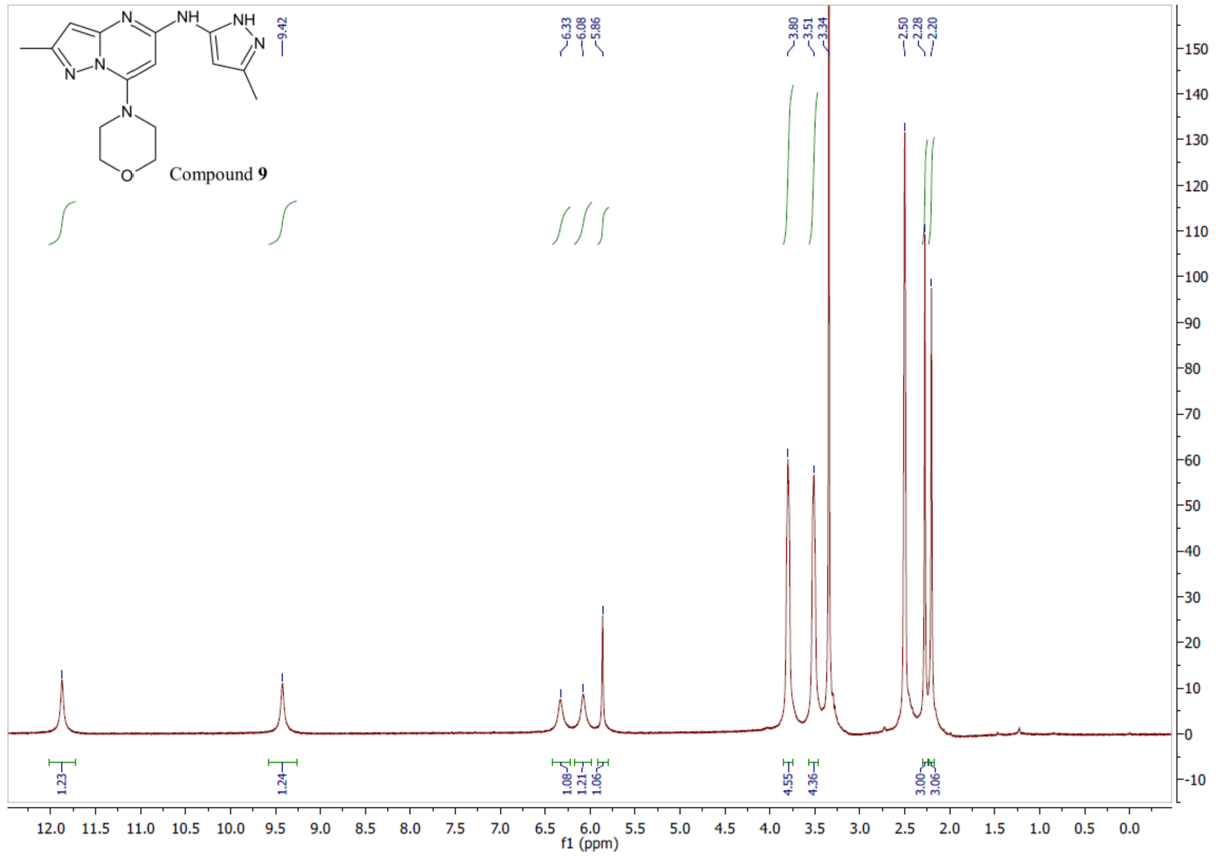

Compound 10

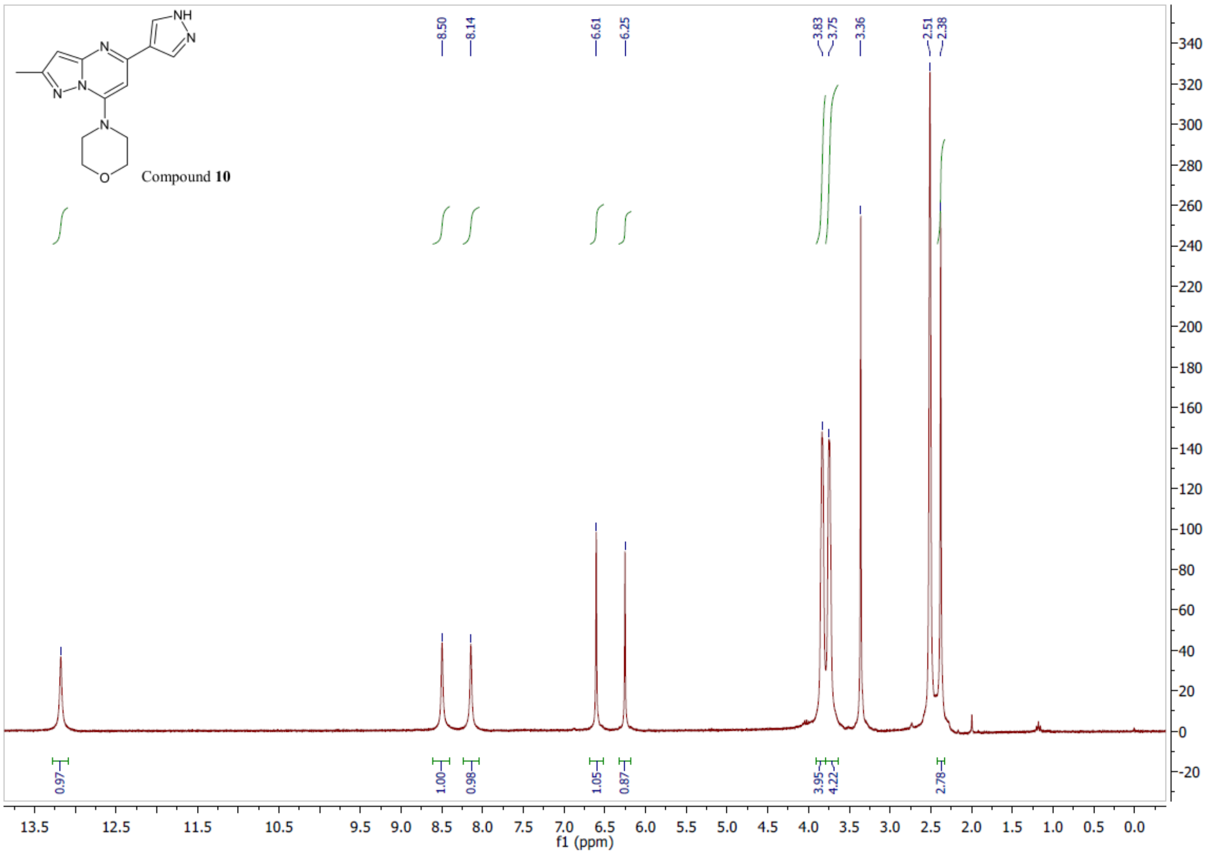

Compound 11

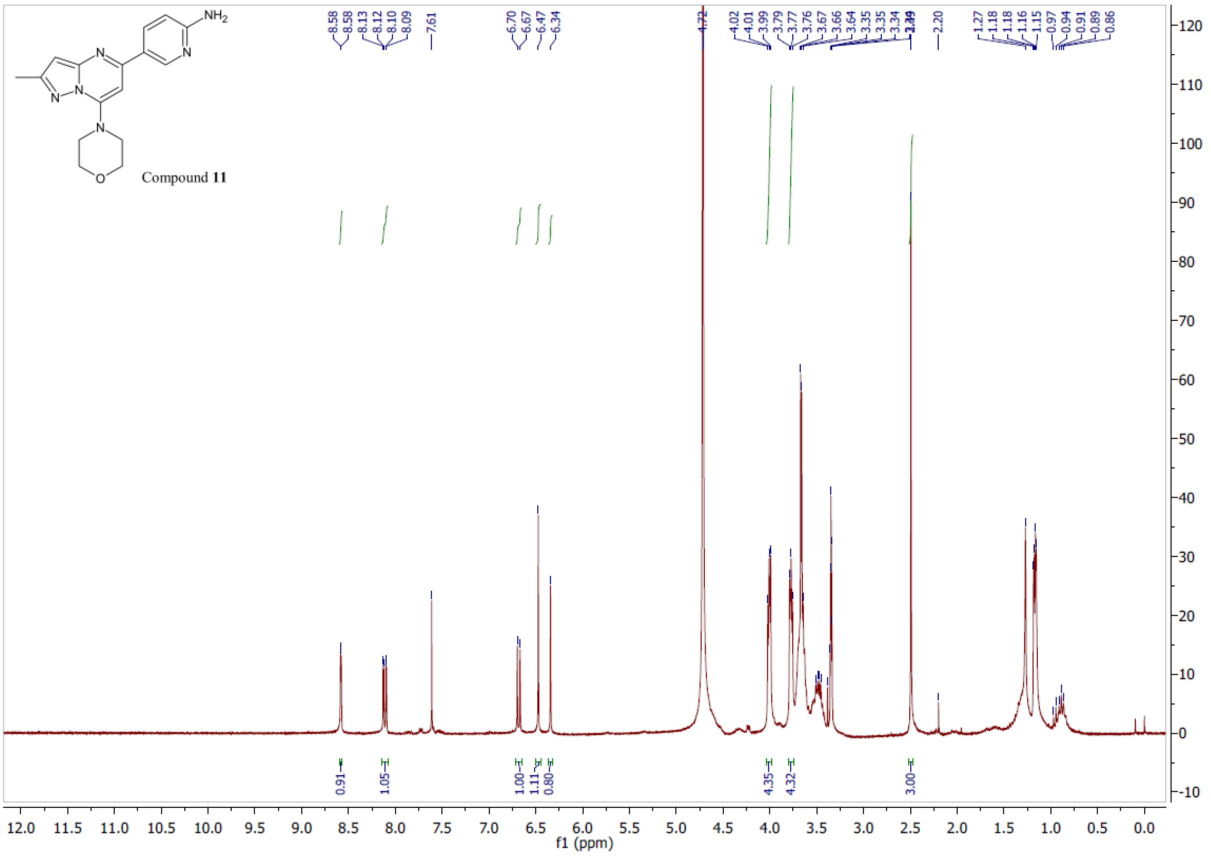

Compound 12

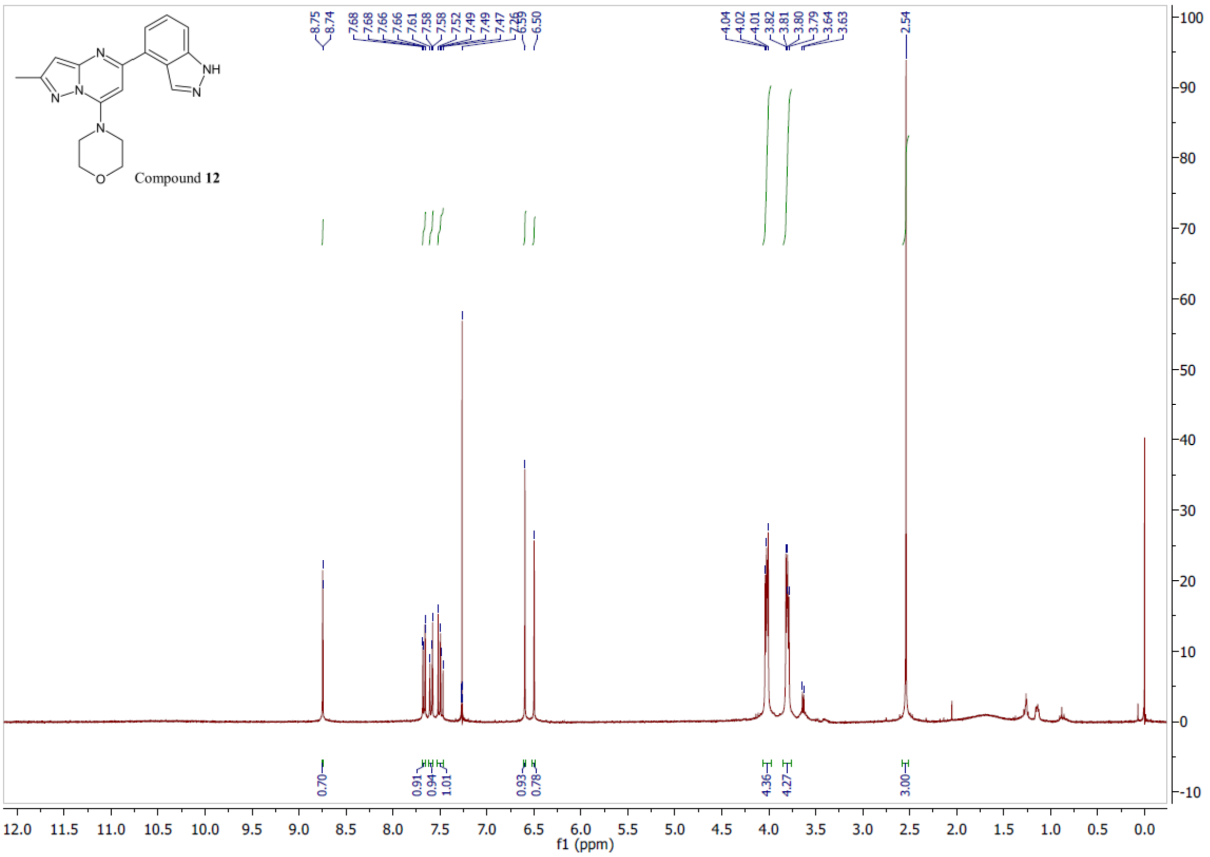

Compound 13

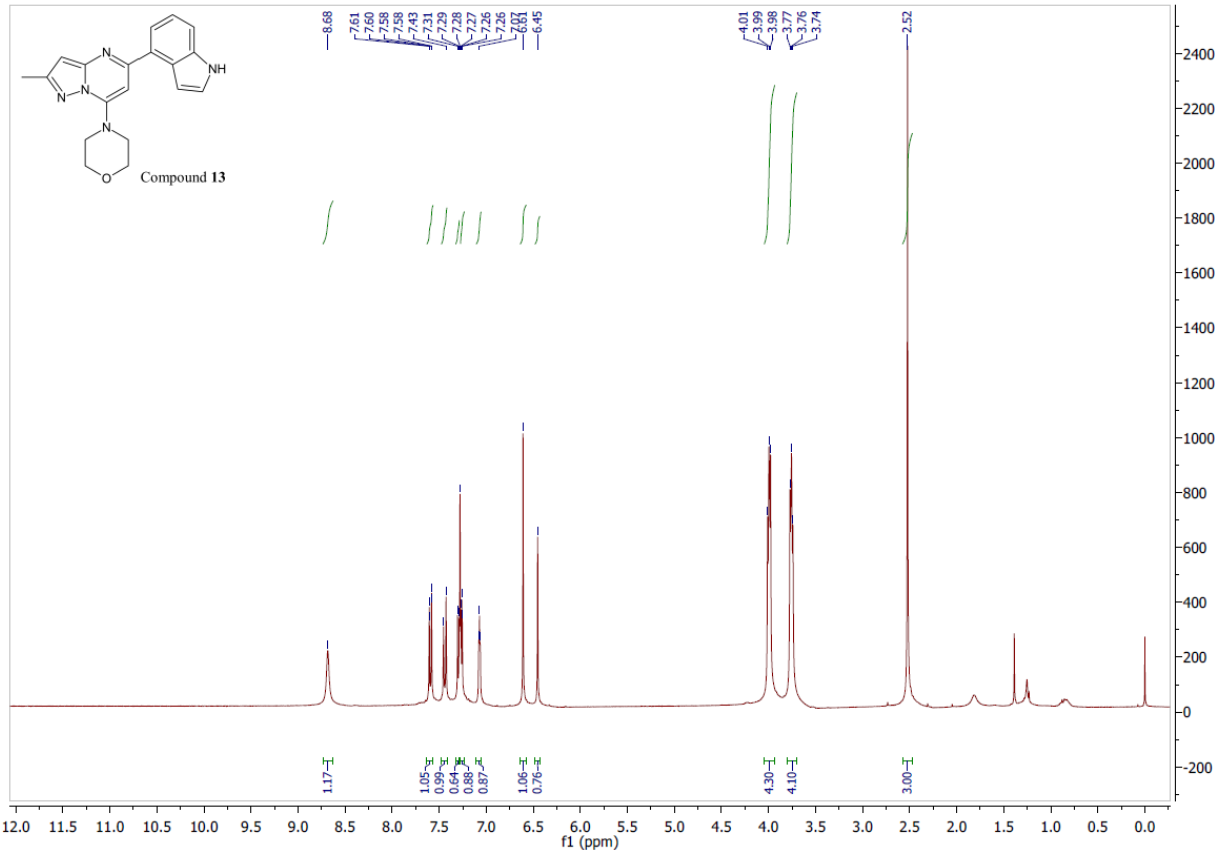

Compound 23

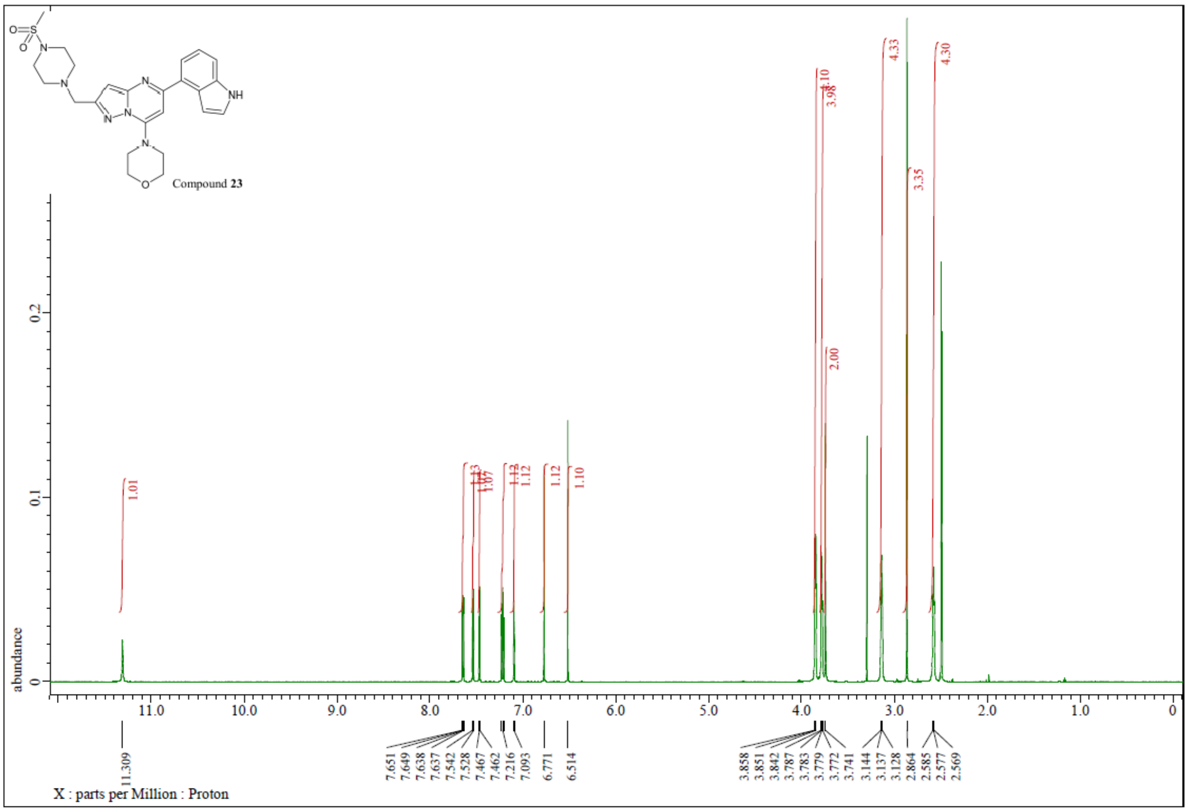
Compound 23
abundance
X : parts per Million : Proton

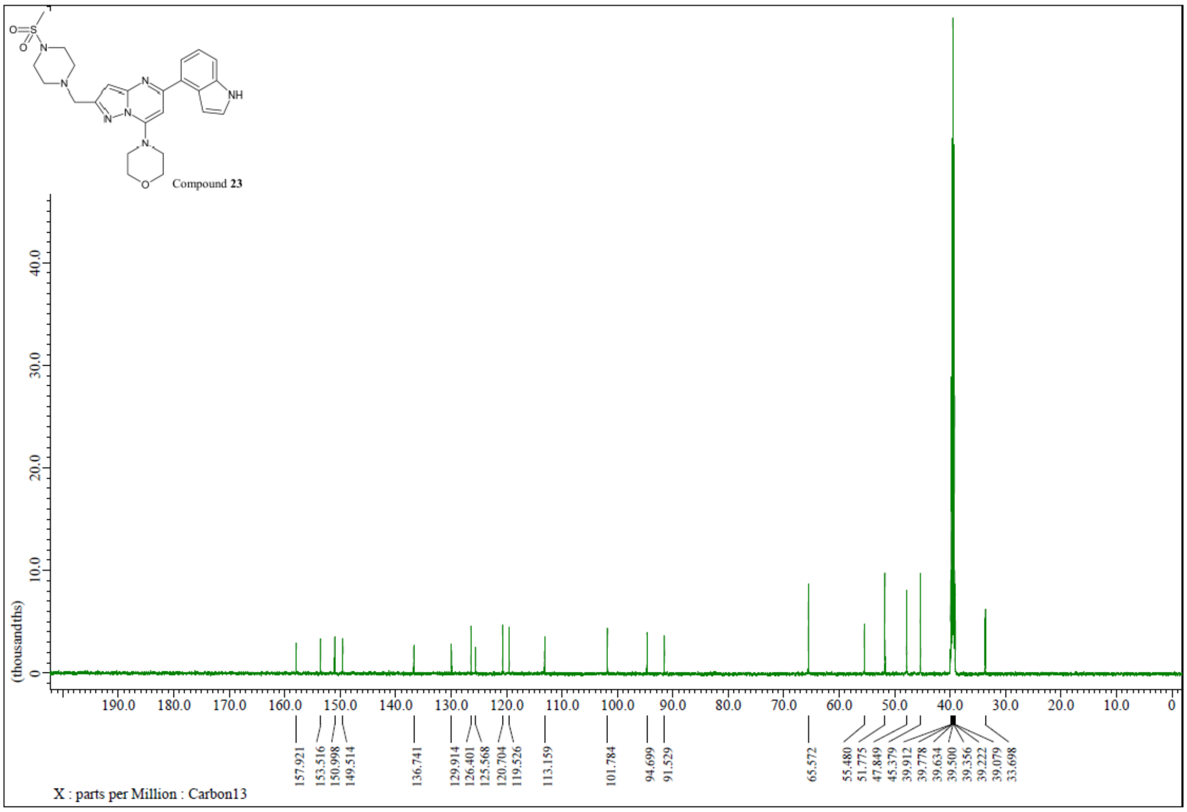
Compound 23
(thousandths)
X : parts per Million : Carbon13

Compound 23

Name	*Obs. m/z*	Obs. RT	Obs. Mass	Tgt Formula	Tgt Mass	Tgt Mass Error	Find Cpds Algorit
Compound **23**	248.61037	1.2	495.2061	C24 H29 N7 O3 S	495.20526	1.69	Find by Formula

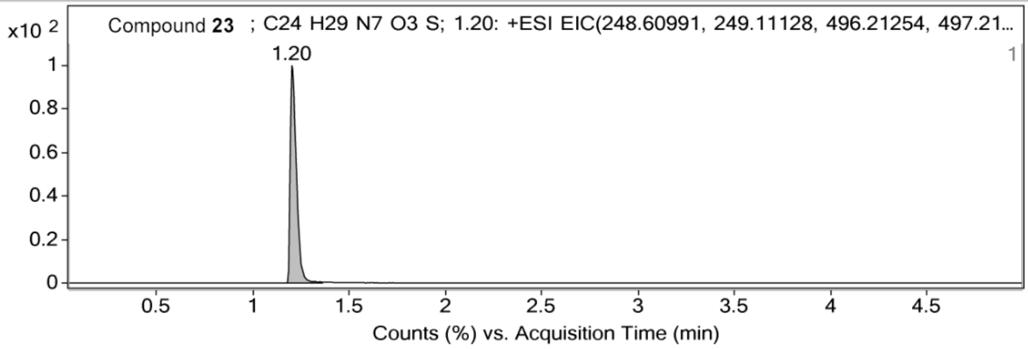

MS Zoomed Spectrum

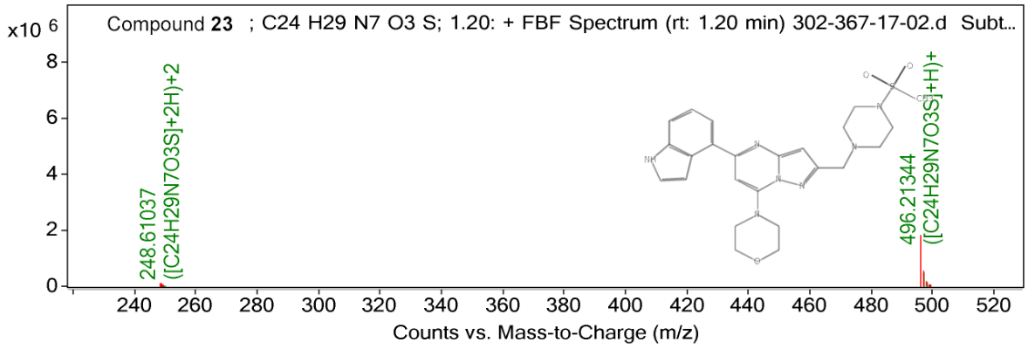

MS Zoomed Spectrum

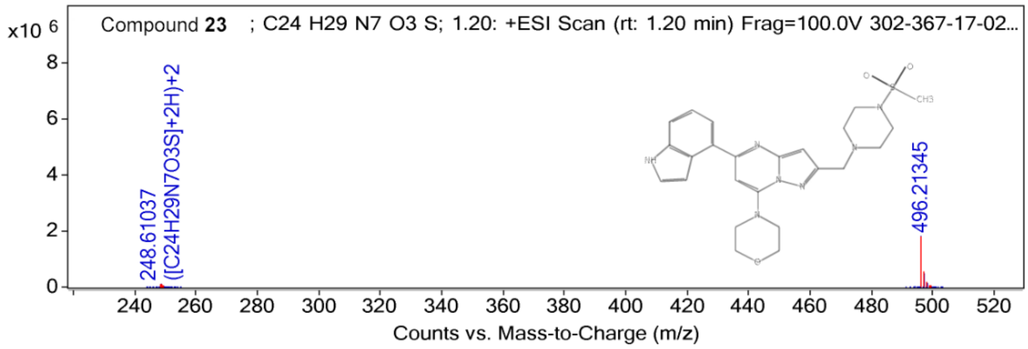

MS Spectrum Peak List

Obs. m/z	Charge	Abund	Formula	Ion/Isotope	Tgt Mass Error (ppm)
248.61037	2	102185.98	C24H29N7O3S	(M+2H)+2	
249.11171	2	27759.85	C24H29N7O3S	(M+2H)+2	
249.6106	2	9742.93	C24H29N7O3S	(M+2H)+2	
250.11214	2	2644.55	C24H29N7O3S	(M+2H)+2	
496.21344	1	1807565	C24H29N7O3S	(M+H)+	
497.2157	1	473910.06	C24H29N7O3S	(M+H)+	
498.21467	1	127993.41	C24H29N7O3S	(M+H)+	
499.21422	1	21856.06	C24H29N7O3S	(M+H)+	
248.61037	2	102185.98	C24H29N7O3S	(M+2H)+2	1.85
249.11171	2	27759.85	C24H29N7O3S	(M+2H)+2	1.72
249.6106	2	9742.93	C24H29N7O3S	(M+2H)+2	1.25
250.11214	2	2644.55	C24H29N7O3S	(M+2H)+2	5.99
496.21344	1	1807565	C24H29N7O3S	(M+H)+	1.83
496.21345		1811059.88			
497.2157	1	473910.06	C24H29N7O3S	(M+H)+	0.84
498.21467	1	127993.41	C24H29N7O3S	(M+H)+	2.74
499.21422	1	21856.06	C24H29N7O3S	(M+H)+	0.44

Compound 24

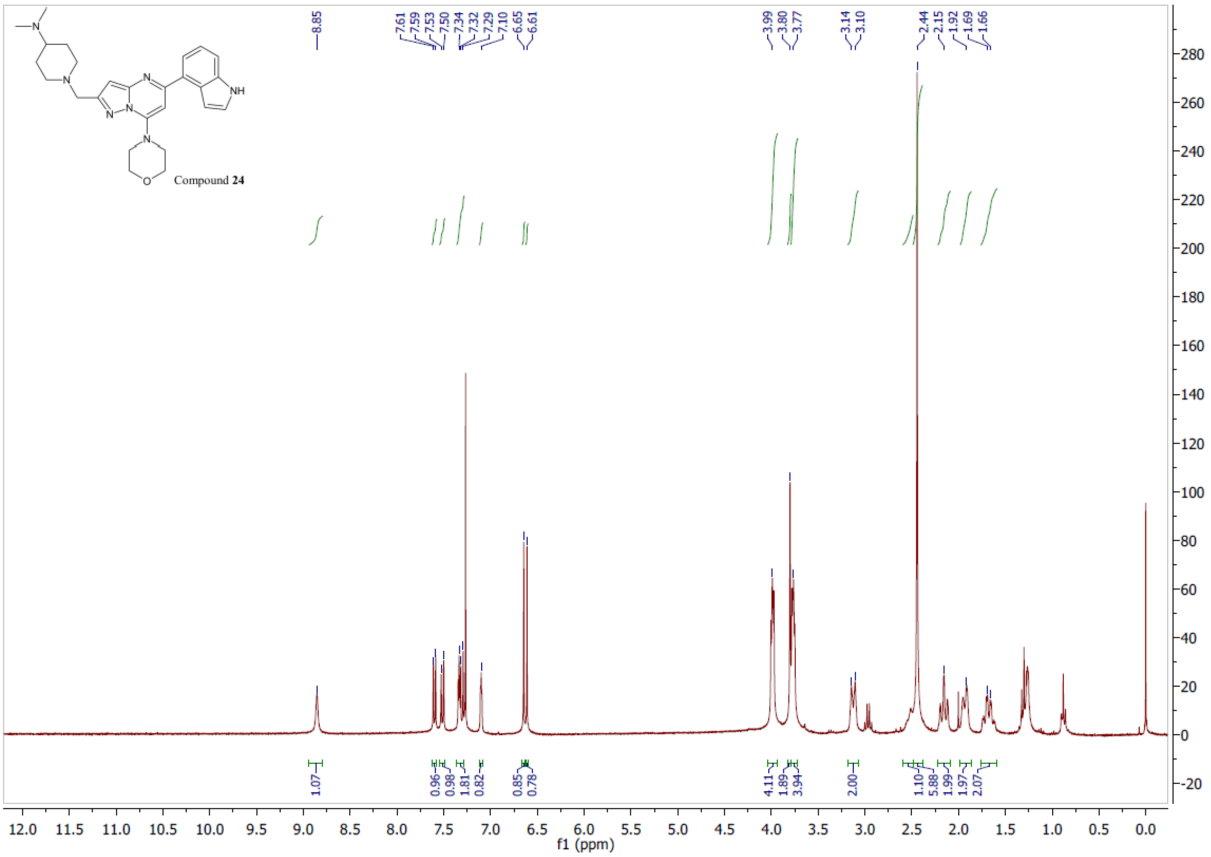

Compound 24

Name	*Obs. m/z*	Obs. RT	Obs. Mass	Tgt Formula	Tgt Mass	Tgt Mass Error	Find Cpds Algorith
Compound **24**	460.2842	1.02	459.2768	C26 H33 N7 O	459.2747	4.76	Find by Formula

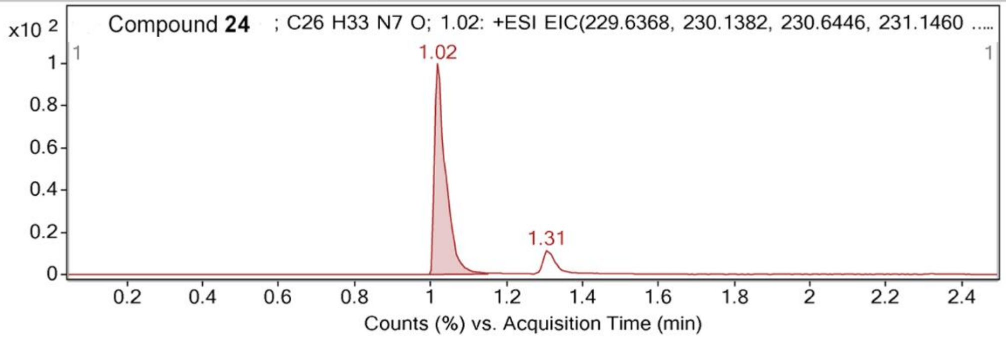

MS Zoomed Spectrum

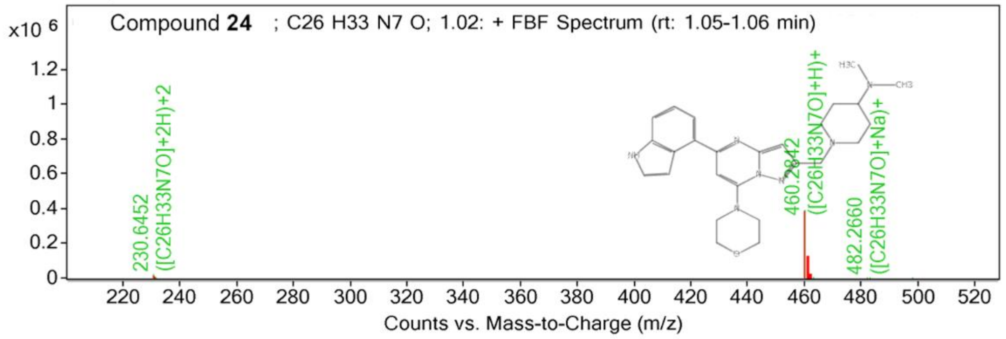

MS Zoomed Spectrum

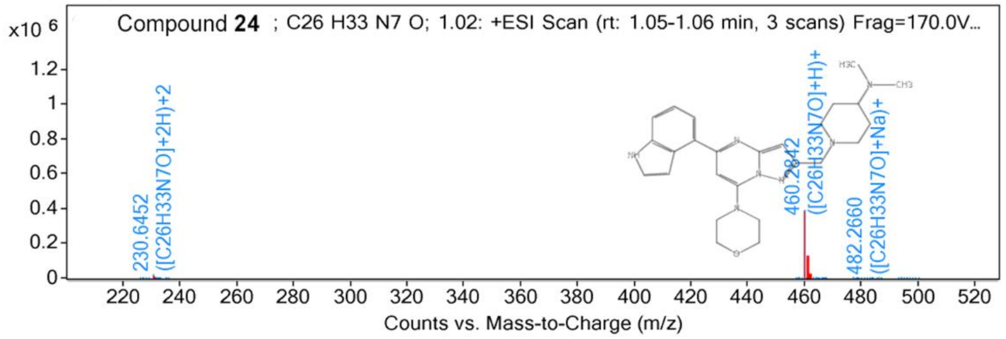

MS Spectrum Peak List

Obs. m/z	Charge	Abund	Formula	Ion/Isotope	Tgt Mass Error (ppm)
460.2842	1	387752.75	C26H33N7O	(M+H)+	
460.2842	1	387752.75	C26H33N7O	(M+H)+	5

--- End Of Report ---

Compound 25

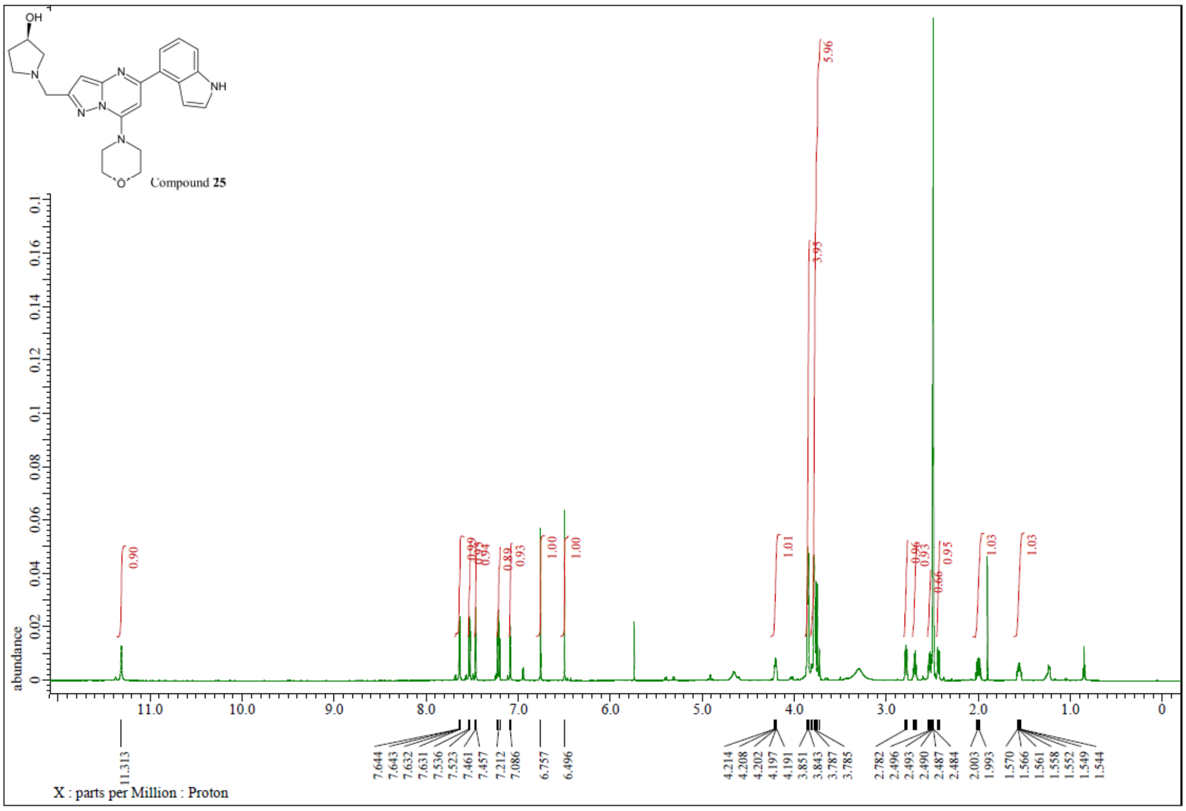
Compound 25
abundance
X : parts per Million : Proton

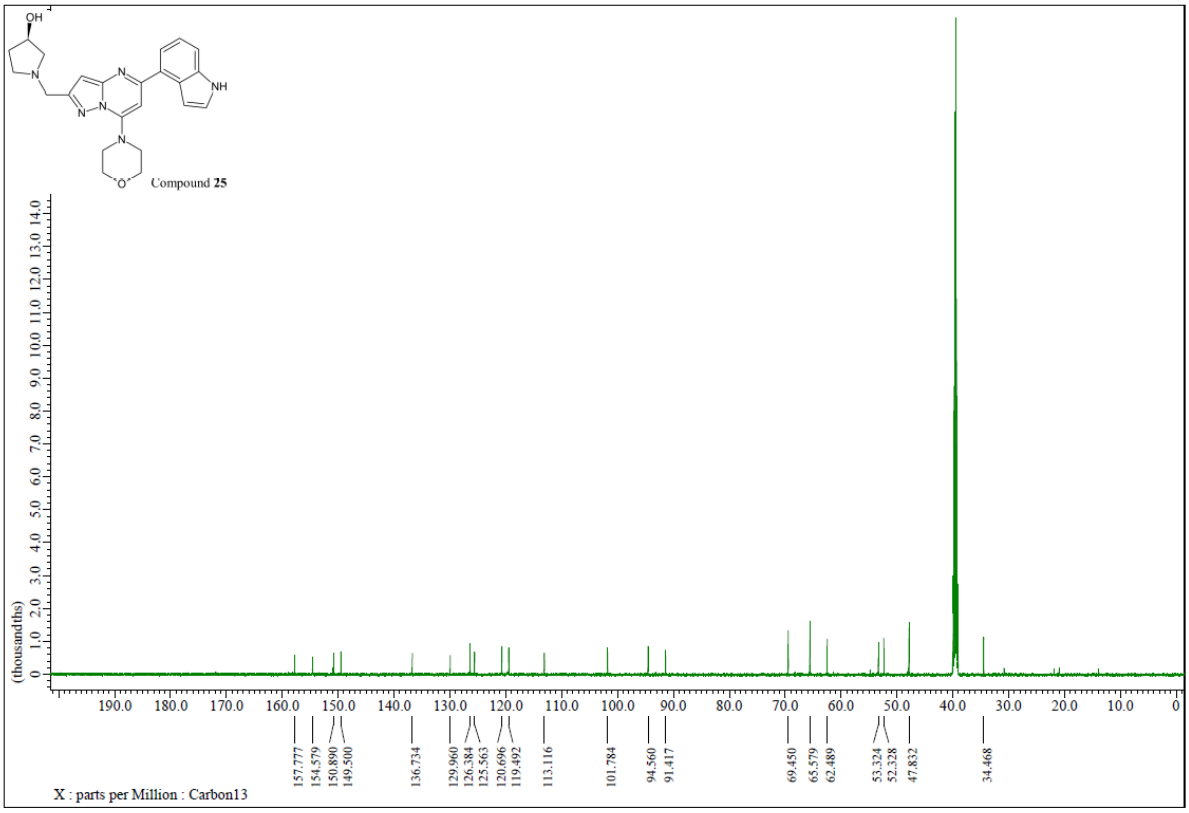
Compound 25
(thousandths)
X : parts per Million : Carbon13

Compound 25

Name	*Obs. m/z*	Obs. RT	Obs. Mass	Tgt Formula	Tgt Mass	Tgt Mass Error	Find Cpds Algorit
Compound **25**	419.21911	1.07	418.21196	C23 H26 N6 O2	418.21172	0.57	Find by Formula

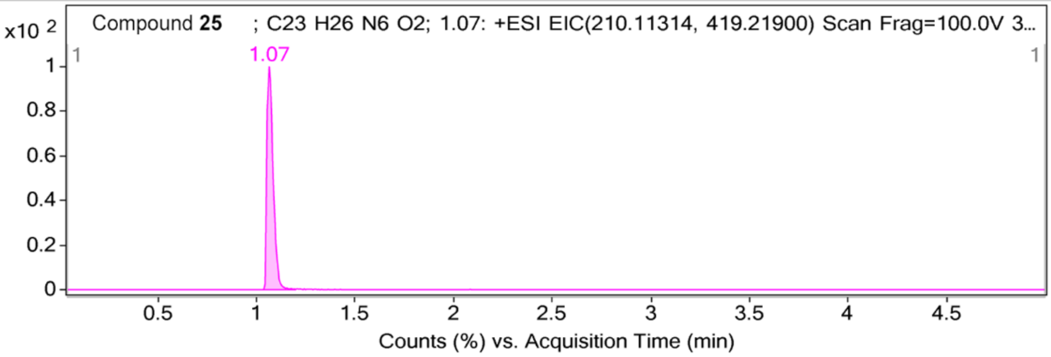

MS Zoomed Spectrum

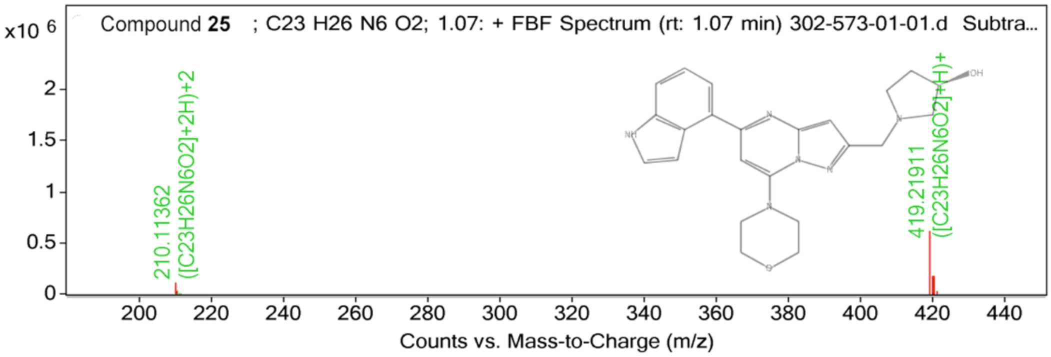

MS Zoomed Spectrum

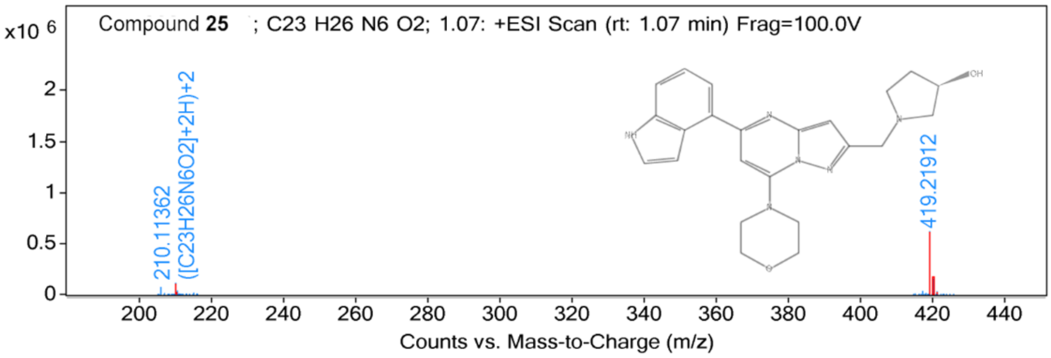

MS Spectrum Peak List

Obs. m/z	Charge	Abund	Formula	Ion/Isotope	Tgt Mass Error (ppm)
210.11362	2	107105.88	C23H26N6O2	(M+2H)+2	
210.61466	2	33089.72	C23H26N6O2	(M+2H)+2	
211.11704	2	4649.13	C23H26N6O2	(M+2H)+2	
211.61535	2	736.03	C23H26N6O2	(M+2H)+2	
419.21911	1	612612	C23H26N6O2	(M+H)+	
420.22217	1	149687.05	C23H26N6O2	(M+H)+	
421.2243	1	22332.43	C23H26N6O2	(M+H)+	
210.11362	2	107105.88	C23H26N6O2	(M+2H)+2	2.31
210.61466	2	33089.72	C23H26N6O2	(M+2H)+2	0.38
211.11704	2	4649.13	C23H26N6O2	(M+2H)+2	5.23
211.61535	2	736.03	C23H26N6O2	(M+2H)+2	-8.95
419.21911	1	612612	C23H26N6O2	(M+H)+	0.27
419.21912		613355.75			
420.22217	1	149687.05	C23H26N6O2	(M+H)+	0.68
421.2243	1	22332.43	C23H26N6O2	(M+H)+	-0.71

--- End Of Report ---

Compound 26

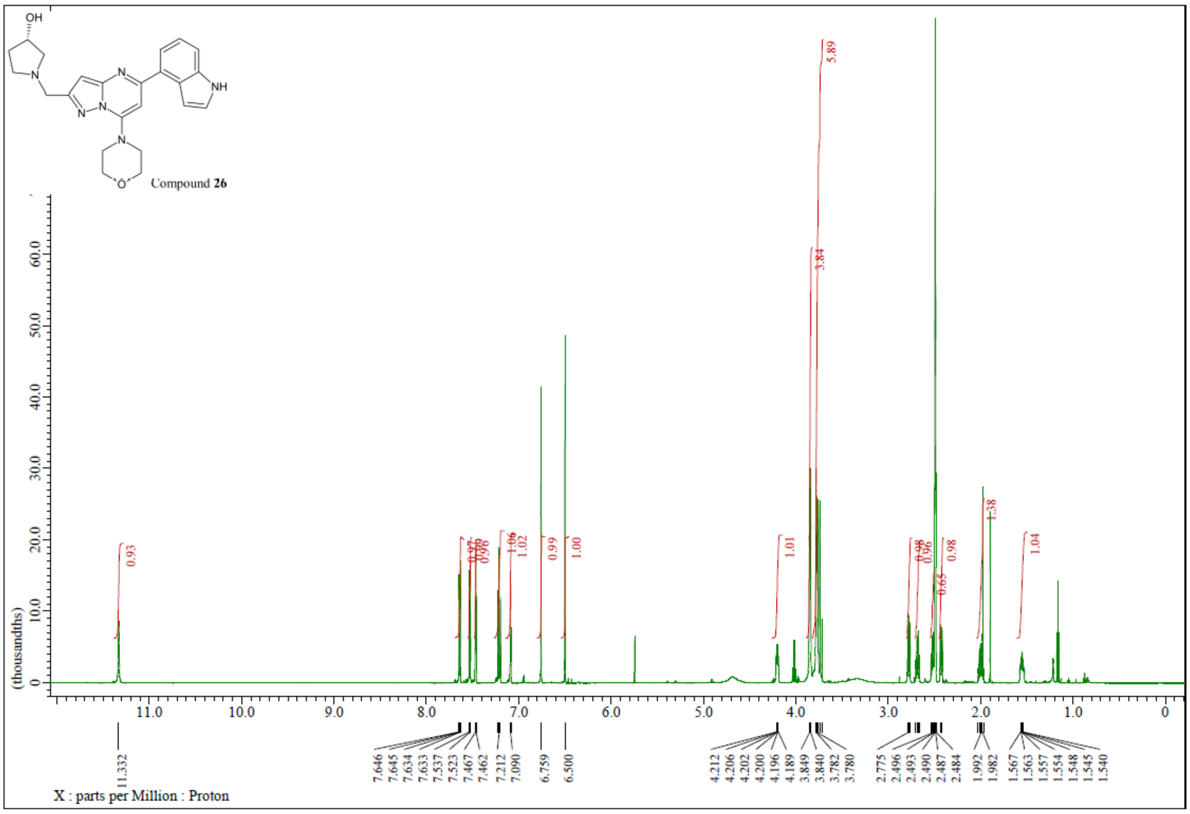
Compound 26
(thousandths)
X : parts per Million : Proton
11.332
7.646
7.645
7.634
7.633
7.537
7.523
7.467
7.462
7.212
7.090
6.759
6.500
4.212
4.206
4.202
4.200
4.196
4.189
3.849
3.840
3.782
3.780
2.775
2.496
2.493
2.490
2.487
2.484
1.992
1.982
1.567
1.563
1.557
1.554
1.548
1.545
1.540

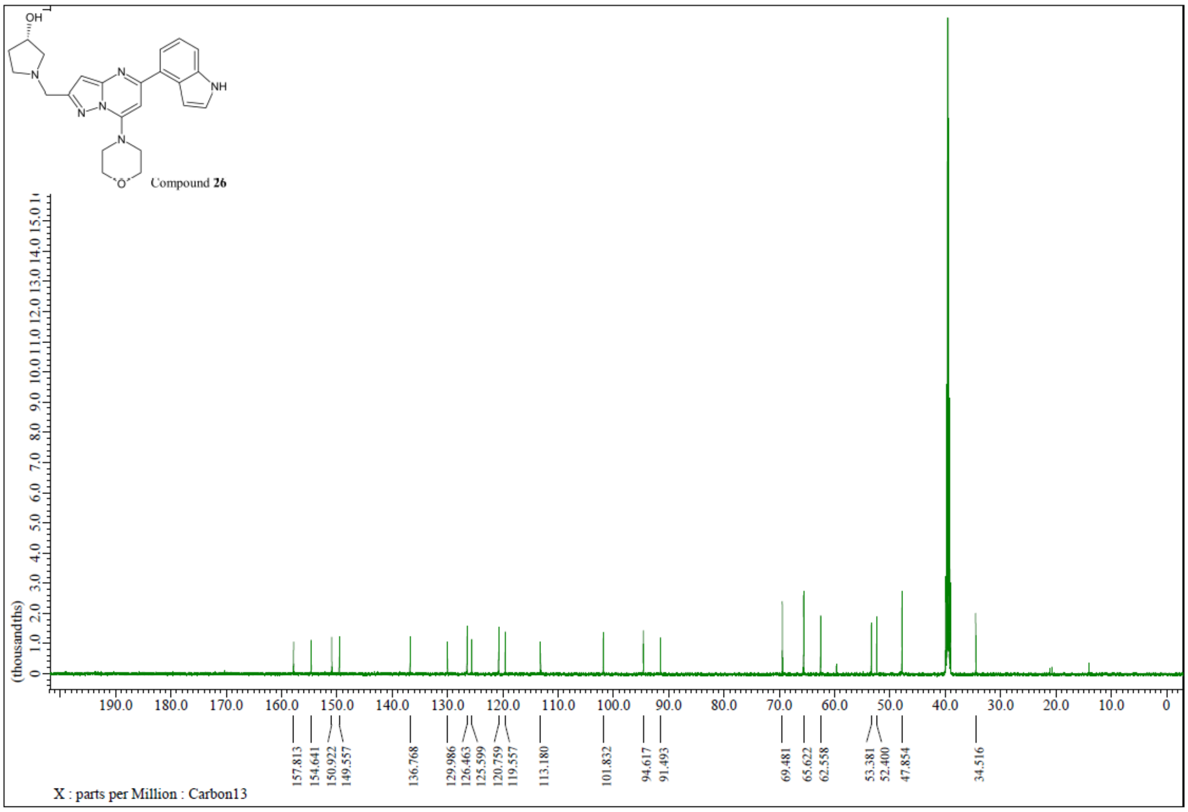
Compound 26
(thousandths)
X : parts per Million : Carbon13
157.813
154.641
150.922
149.557
136.768
129.986
126.463
125.599
120.759
119.557
113.180
101.832
94.617
91.493
69.481
65.622
62.558
53.381
52.400
47.854
34.516

Compound 26

Name	*Obs. m/z*	Obs. RT	Obs. Mass	Tgt Formula	Tgt Mass	Tgt Mass Error	Find Cpds Algorit
Compound **26**	210.11353	1.06	418.21255	C23 H26 N6 O2	418.21172	1.98	Find by Formula

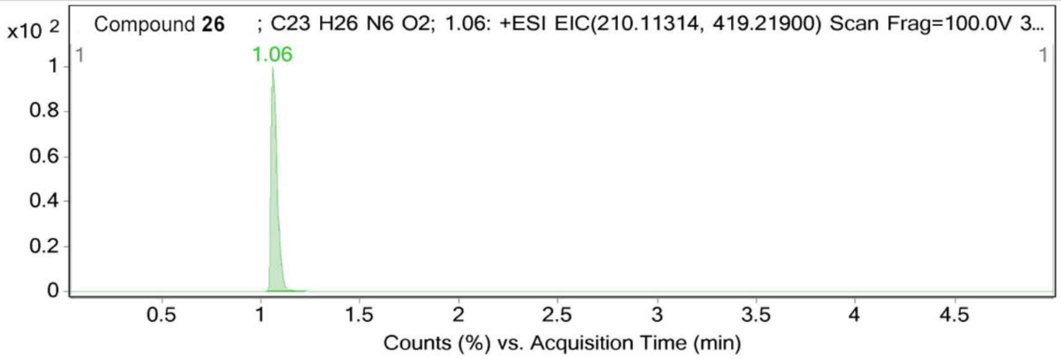

MS Zoomed Spectrum

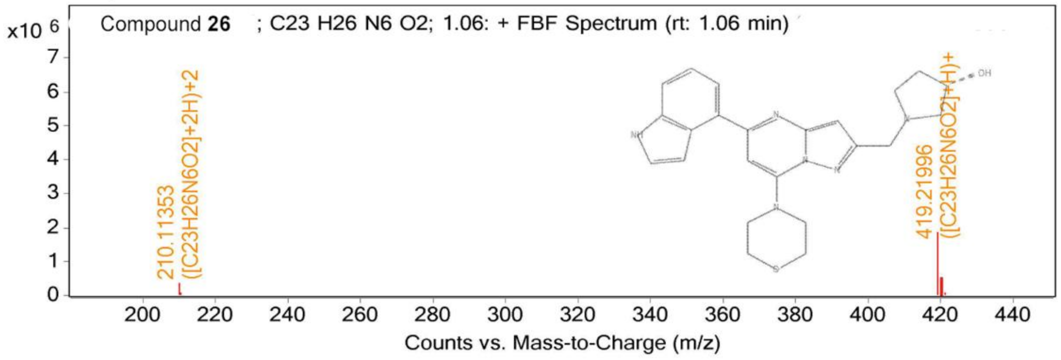

MS Zoomed Spectrum

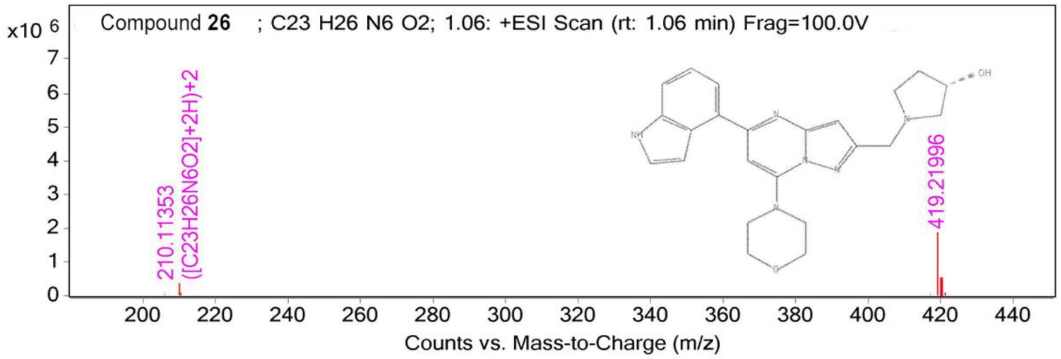

MS Spectrum Peak List

Obs. m/z	Charge	Abund	Formula	Ion/Isotope	Tgt Mass Error (ppm)
210.11353	2	350755.53	C23H26N6O2	(M+2H)+2	
210.61496	2	90613.7	C23H26N6O2	(M+2H)+2	
211.11719	2	12392.01	C23H26N6O2	(M+2H)+2	
419.21996	1	1863211.5	C23H26N6O2	(M+H)+	
420.22229	1	447282	C23H26N6O2	(M+H)+	
421.2245	1	65189.49	C23H26N6O2	(M+H)+	
210.11353	2	350755.53	C23H26N6O2	(M+2H)+2	1.86
210.61496	2	90613.7	C23H26N6O2	(M+2H)+2	1.81
211.11719	2	12392.01	C23H26N6O2	(M+2H)+2	5.92
419.21996	1	1863211.5	C23H26N6O2	(M+H)+	2.29
419.21996		1864620.63			
420.22229	1	447282	C23H26N6O2	(M+H)+	0.97
421.2245	1	65189.49	C23H26N6O2	(M+H)+	-0.23

--- End Of Report ---

Compound 27

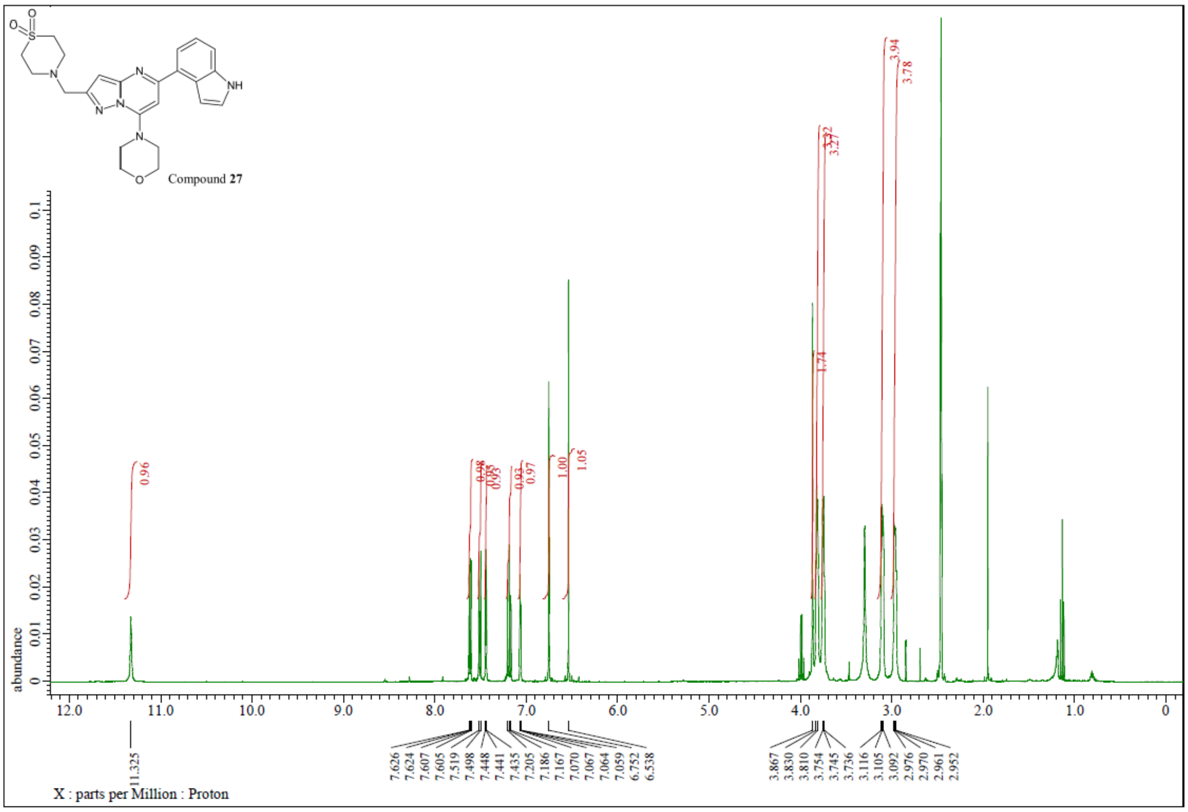

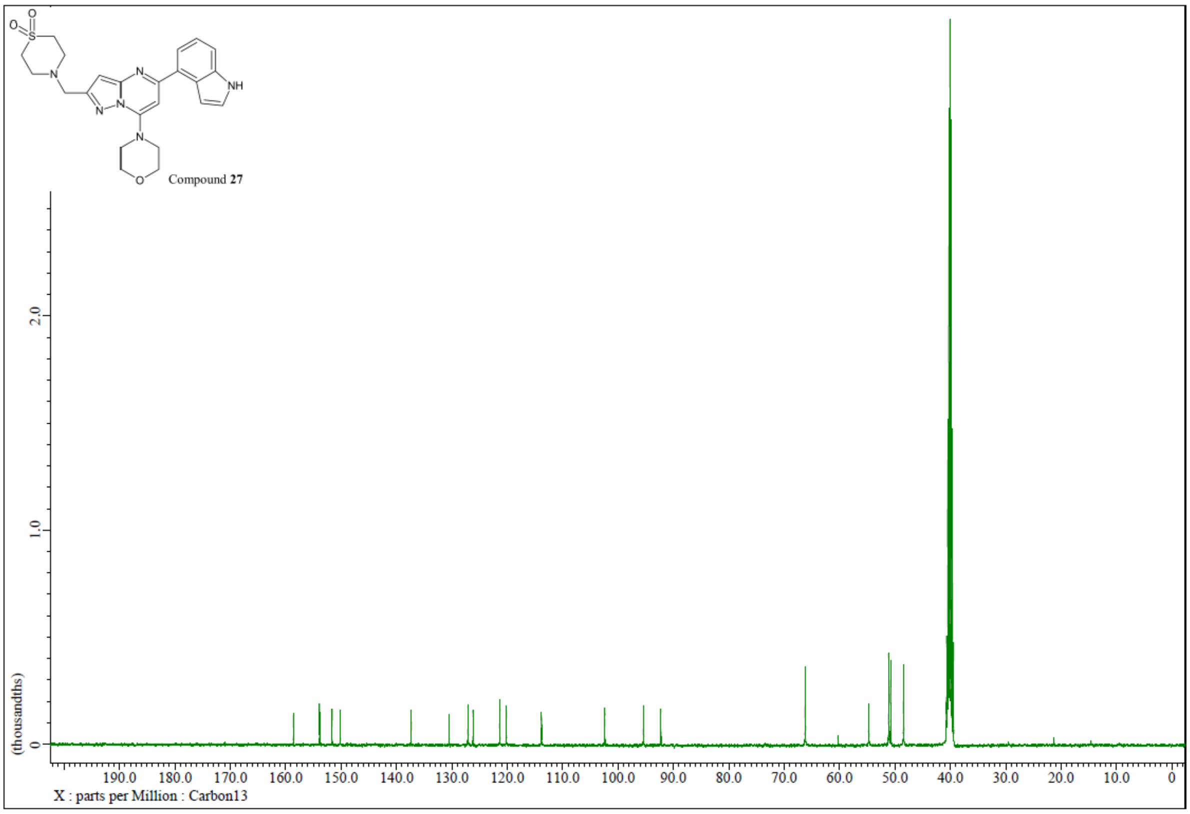

Compound 27

Name	*Obs. m/z*	Obs. RT	Obs. Mass	Tgt Formula	Tgt Mass	Tgt Mass Error	Find Cpds Algorit
Compound **27**	467.18662	1.37	466.17936	C23 H26 N6 O3 S	466.17871	1.4	Find by Formula

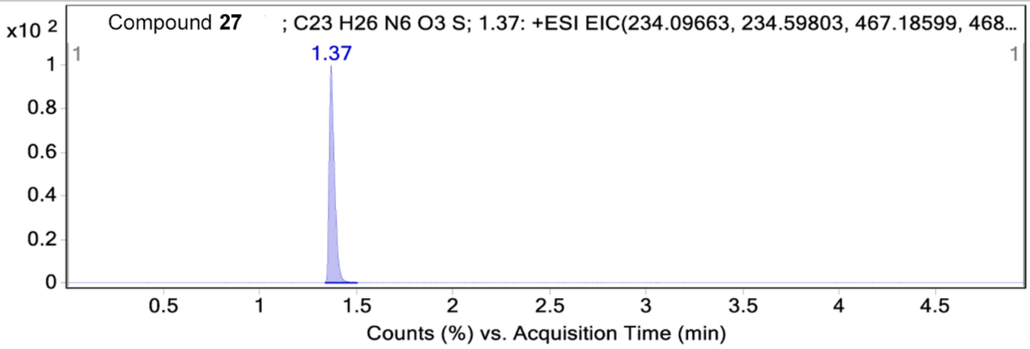

MS Zoomed Spectrum

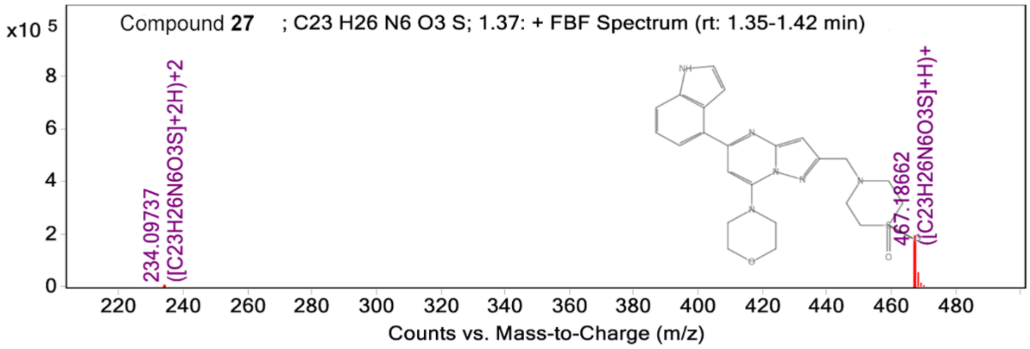

MS Zoomed Spectrum

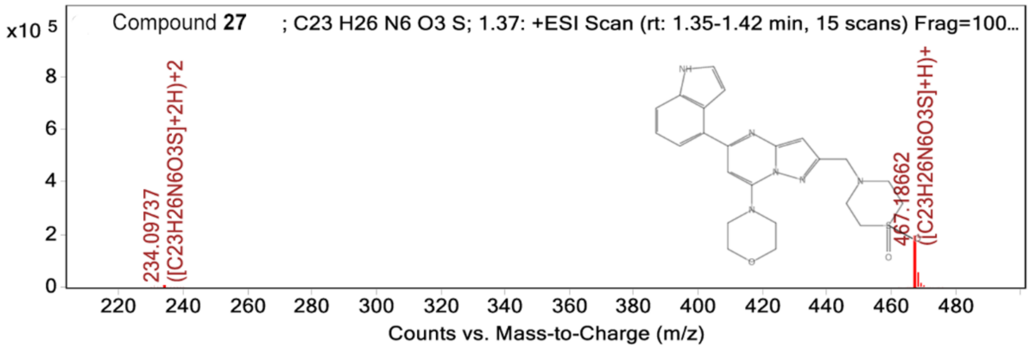

MS Spectrum Peak List

Obs. m/z	Charge	Abund	Formula	Ion/Isotope	Tgt Mass Error (ppm)
234.09737	2	4366.86	C23H26N6O3S	(M+2H)+2	
234.59878	2	1087.41	C23H26N6O3S	(M+2H)+2	
235.09806	2	303.18	C23H26N6O3S	(M+2H)+2	
235.59344	2	49.08	C23H26N6O3S	(M+2H)+2	
467.18662	1	192986.59	C23H26N6O3S	(M+H)+	
468.1894	1	54429.01	C23H26N6O3S	(M+H)+	
469.18746	1	14103.24	C23H26N6O3S	(M+H)+	
470.18826	1	2630.32	C23H26N6O3S	(M+H)+	
471.18539	1	698.5	C23H26N6O3S	(M+H)+	
234.09737	2	4366.86	C23H26N6O3S	(M+2H)+2	3.17
234.59878	2	1087.41	C23H26N6O3S	(M+2H)+2	3.2
235.09806	2	303.18	C23H26N6O3S	(M+2H)+2	4.83
235.59344	2	49.08	C23H26N6O3S	(M+2H)+2	-16.47
467.18662	1	192986.59	C23H26N6O3S	(M+H)+	1.35
468.1894	1	54429.01	C23H26N6O3S	(M+H)+	1.32
469.18746	1	14103.24	C23H26N6O3S	(M+H)+	1.89
470.18826	1	2630.32	C23H26N6O3S	(M+H)+	1.9
471.18539	1	698.5	C23H26N6O3S	(M+H)+	-7.1

--- End Of Report ---

Compound 28

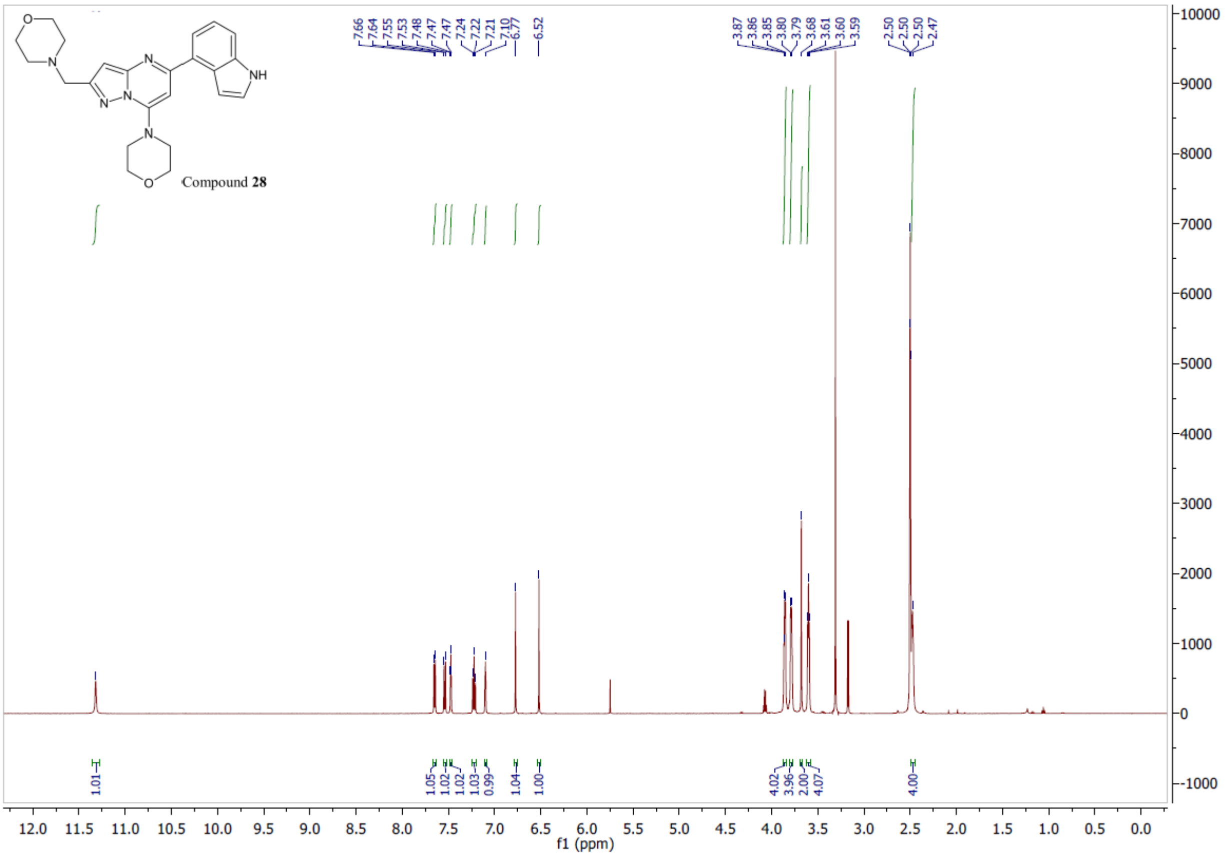

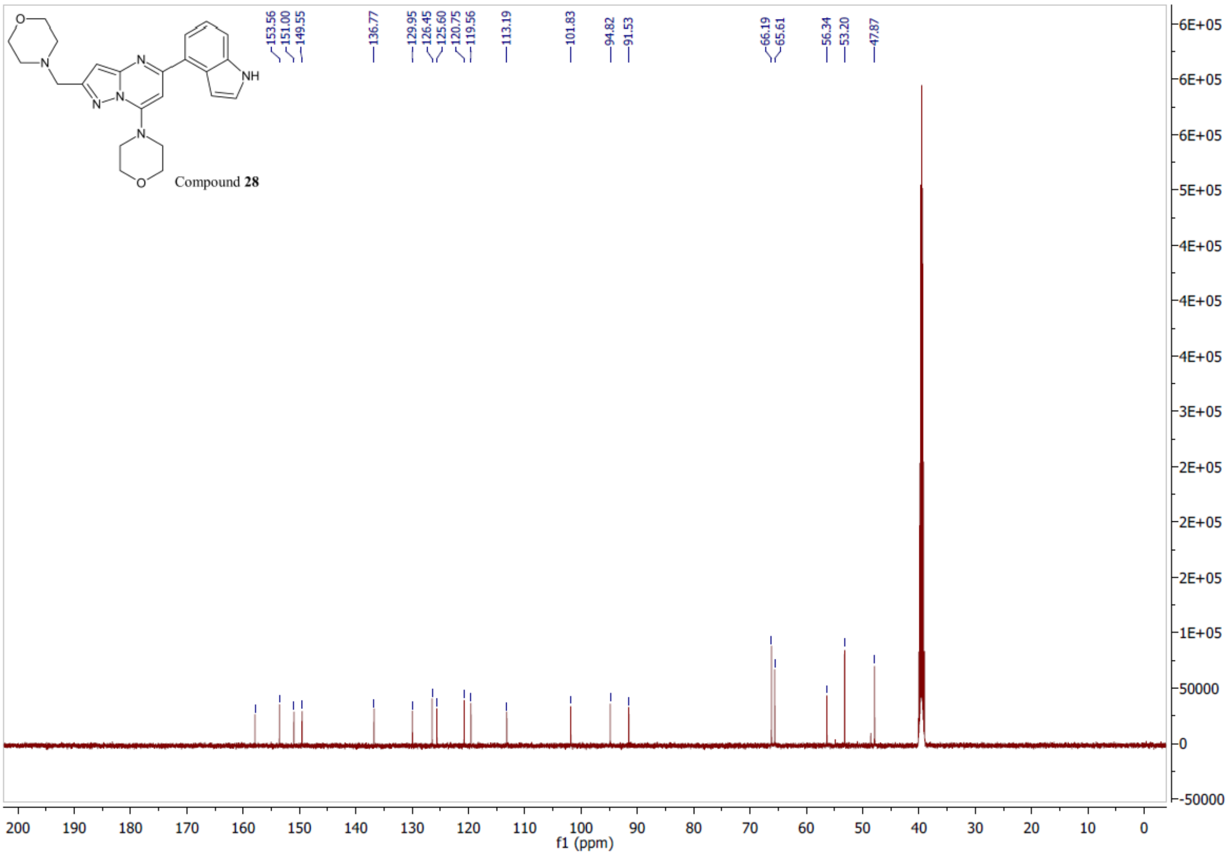

Compound 28

Compound Label	Name	*m/z*	RT	Algorithm	Mass
Compound **28**	Compound **28**	419.21961	1.22	Find by Formula	418.21231

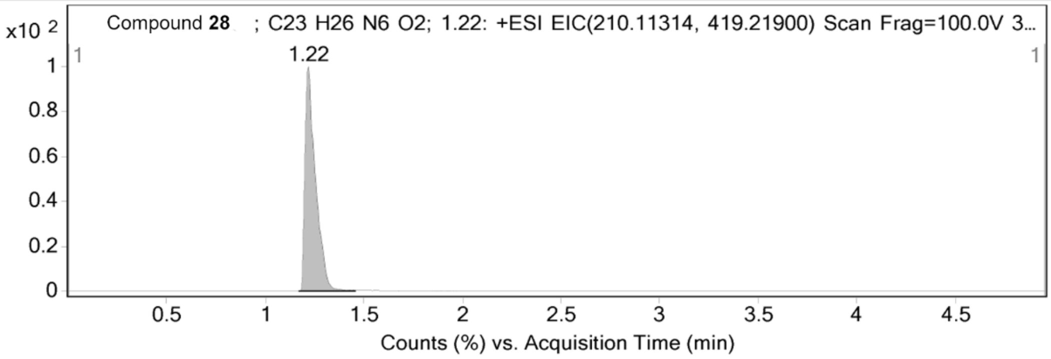

MS Spectrum

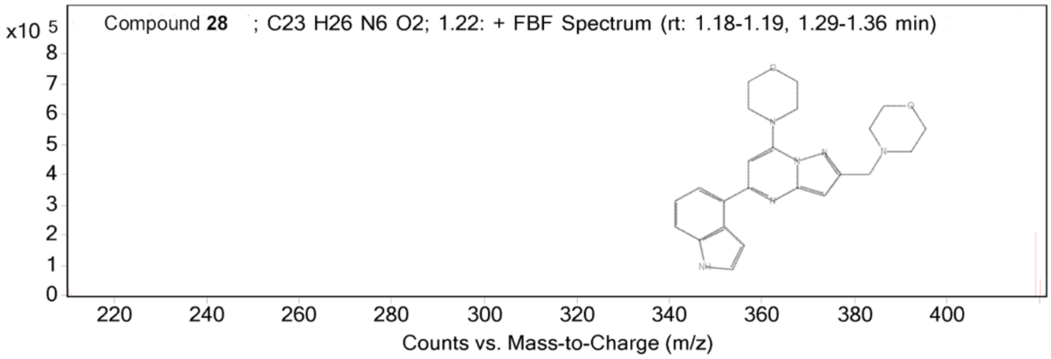

MS Zoomed Spectrum

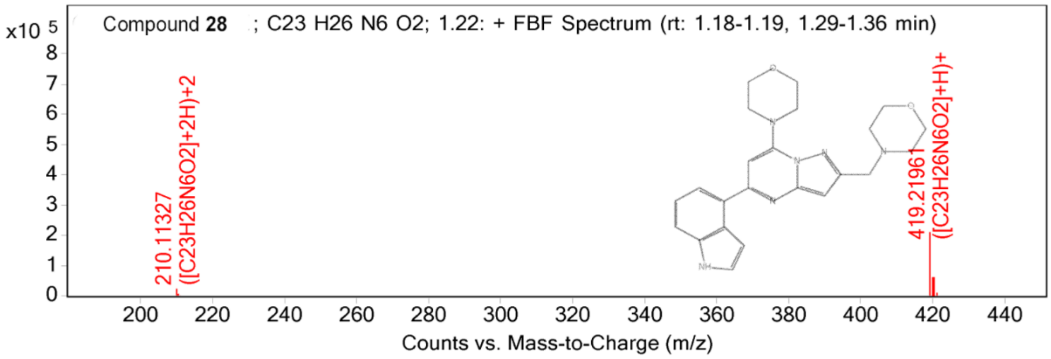

MS Spectrum Peak List

m/z	z	Abund	Formula	Ion
210.11327	2	21627.81	C23H26N6O2	(M+2H)+2
210.6148	2	6590.59	C23H26N6O2	(M+2H)+2
211.11612	2	1038.7	C23H26N6O2	(M+2H)+2
419.21961	1	208915.36	C23H26N6O2	(M+H)+
420.22254	1	53420.48	C23H26N6O2	(M+H)+

Compound 29

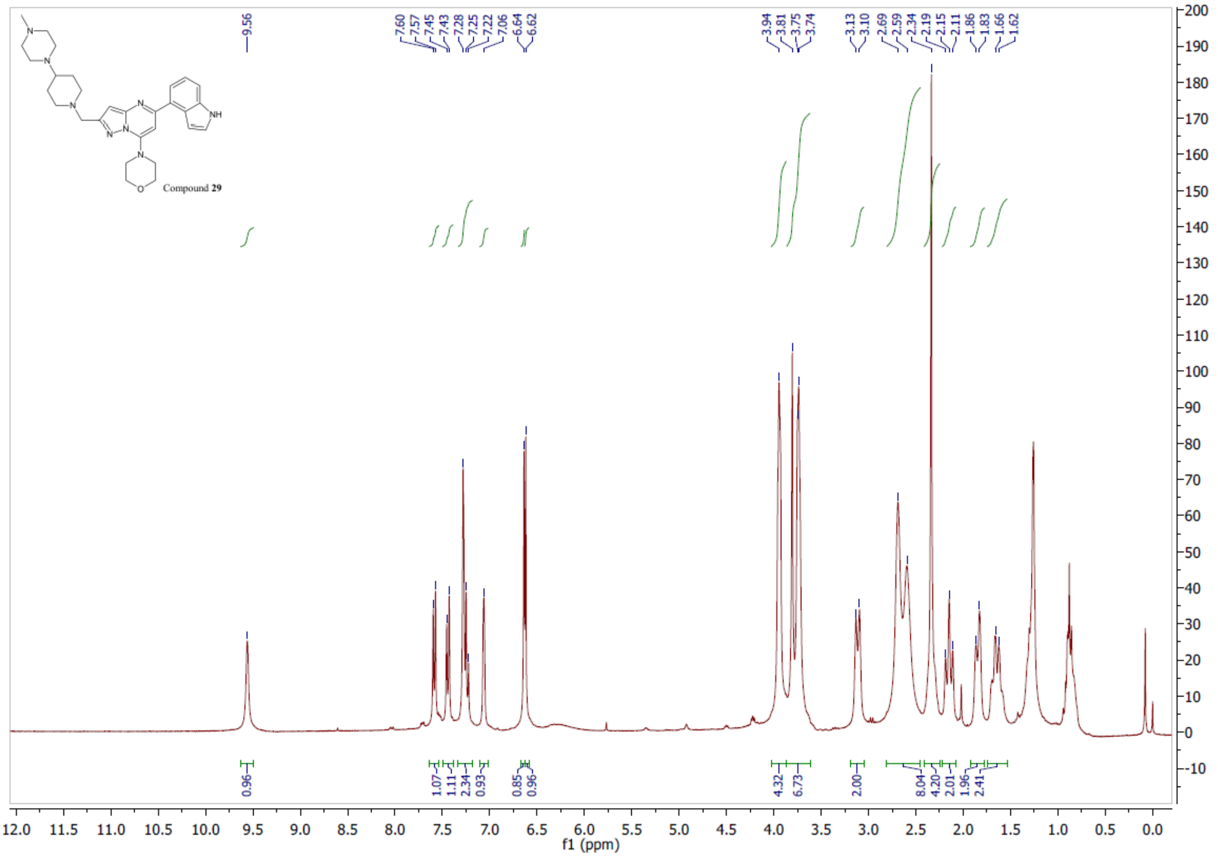
Compound 29
9.56
7.60
7.57
7.45
7.43
7.28
7.25
7.22
7.06
6.64
6.62
3.94
3.81
3.75
3.74
3.13
3.10
2.69
2.59
2.34
2.19
2.15
2.11
1.86
1.83
1.66
1.62
0.96
1.07
1.11
2.34
0.93
0.85
0.96
4.32
6.73
2.00
8.04
4.20
2.01
1.96
2.41
f1 (ppm)

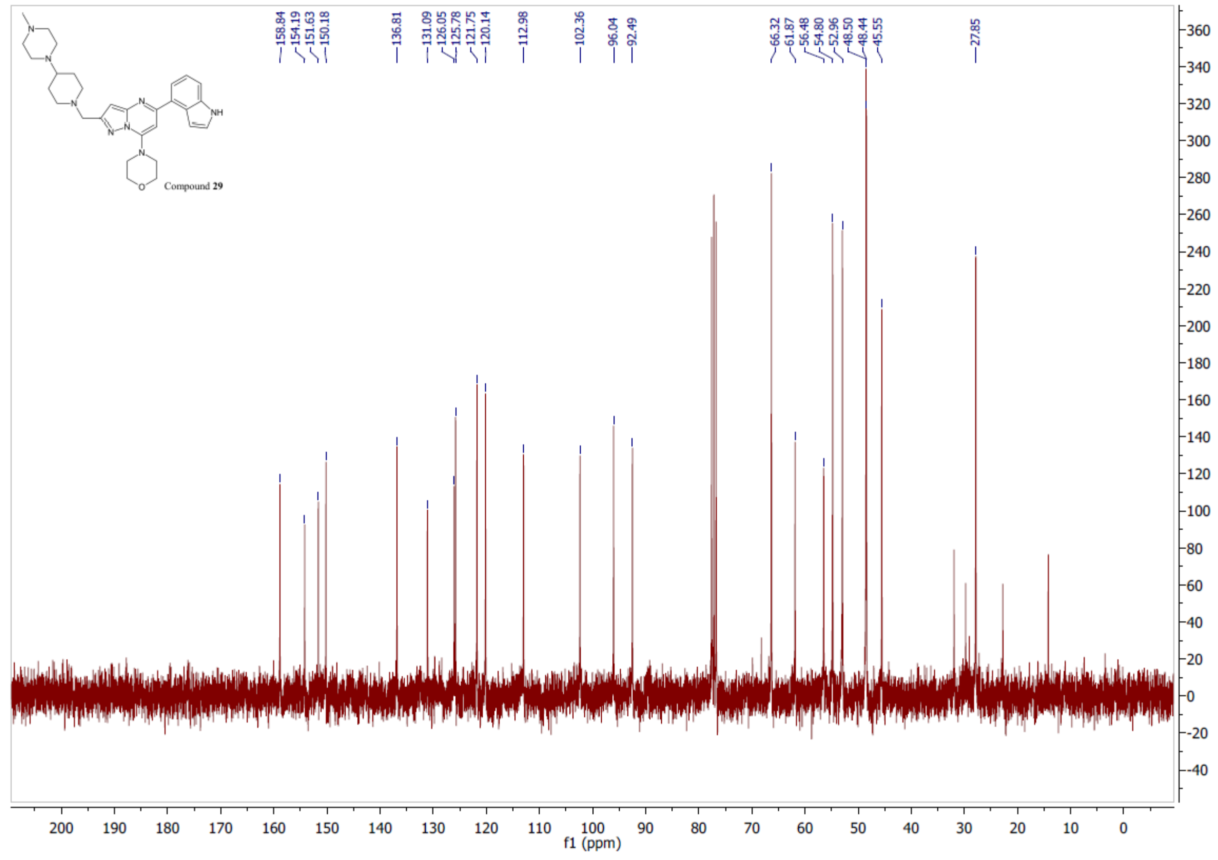
Compound 29
158.84
154.19
151.63
150.18
136.81
131.09
126.05
125.78
121.75
120.14
112.98
102.36
96.04
92.49
66.32
61.87
56.48
54.80
52.96
48.50
48.44
45.55
27.85
f1 (ppm)

Compound 29

Name	*Obs. m/z*	Obs. RT	Obs. Mass	Tgt Formula	Tgt Mass	Tgt Mass Error	Find Cpds Algorith
Compound **29**	258.16623	0.91	514.31764	C29 H38 N8 O	514.31686	1.51	Find by Formula

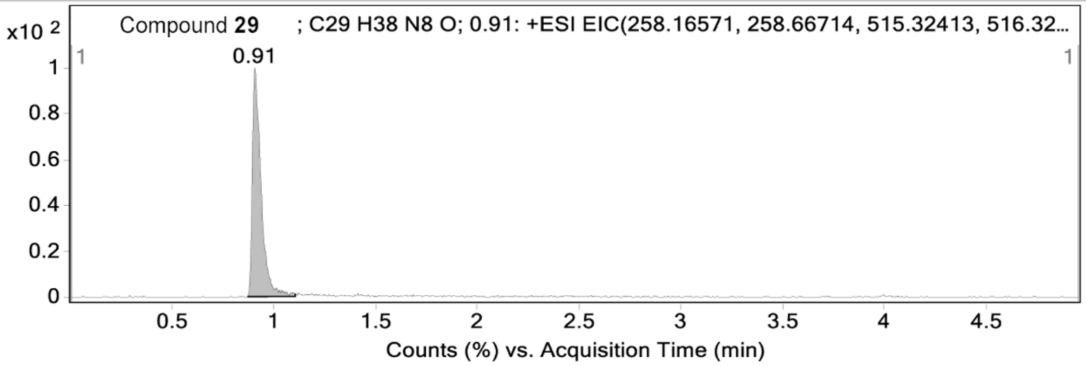

MS Zoomed Spectrum

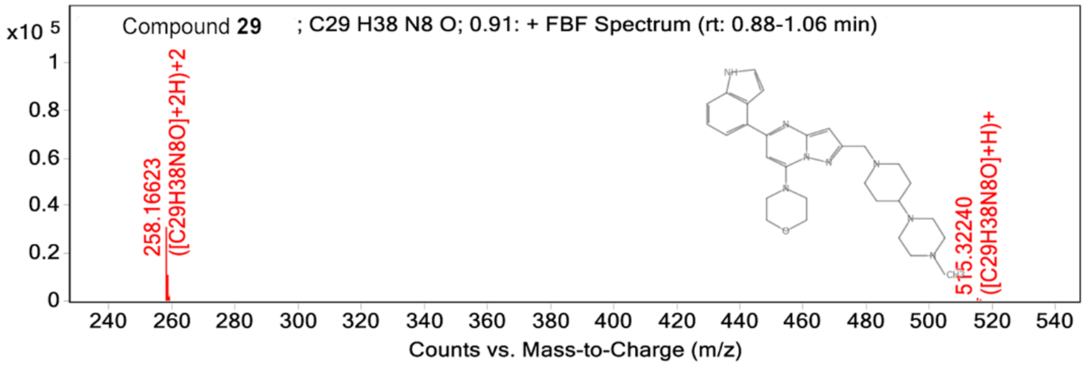

MS Zoomed Spectrum

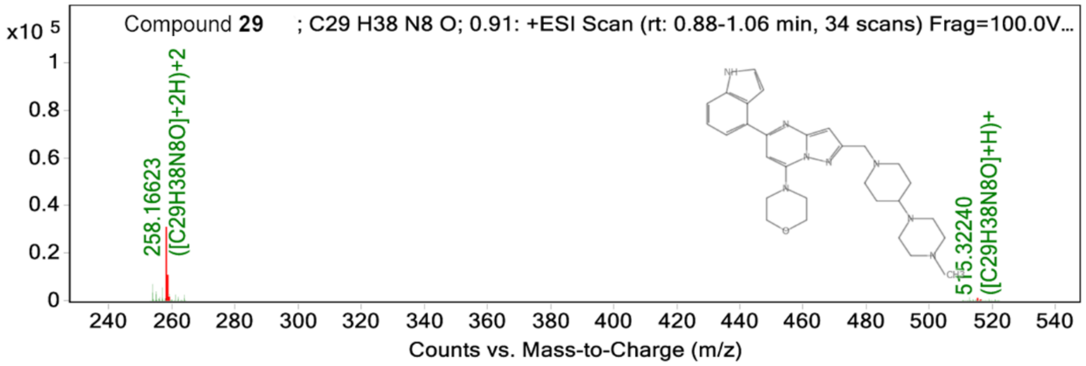

MS Spectrum Peak List

Obs. m/z	Charge	Abund	Formula	Ion/Isotope	Tgt Mass Error (ppm)
258.16623	2	30690.7	C29H38N8O	(M+2H)+2	
258.66762	2	10530.33	C29H38N8O	(M+2H)+2	
259.16777	2	2840.49	C29H38N8O	(M+2H)+2	
259.66954	2	271.58	C29H38N8O	(M+2H)+2	
515.3224	1	978.89	C29H38N8O	(M+H)+	
516.32472	1	341.54	C29H38N8O	(M+H)+	
517.32756	1	45.86	C29H38N8O	(M+H)+	
258.16623	2	30690.7	C29H38N8O	(M+2H)+2	2.05
258.66762	2	10530.33	C29H38N8O	(M+2H)+2	1.87
259.16777	2	2840.49	C29H38N8O	(M+2H)+2	-2.93
259.66954	2	271.58	C29H38N8O	(M+2H)+2	-1.38
515.3224	1	978.89	C29H38N8O	(M+H)+	-3.37
516.32472	1	341.54	C29H38N8O	(M+H)+	-4.42
517.32756	1	45.86	C29H38N8O	(M+H)+	-4.31

--- End Of Report ---

Compound 30

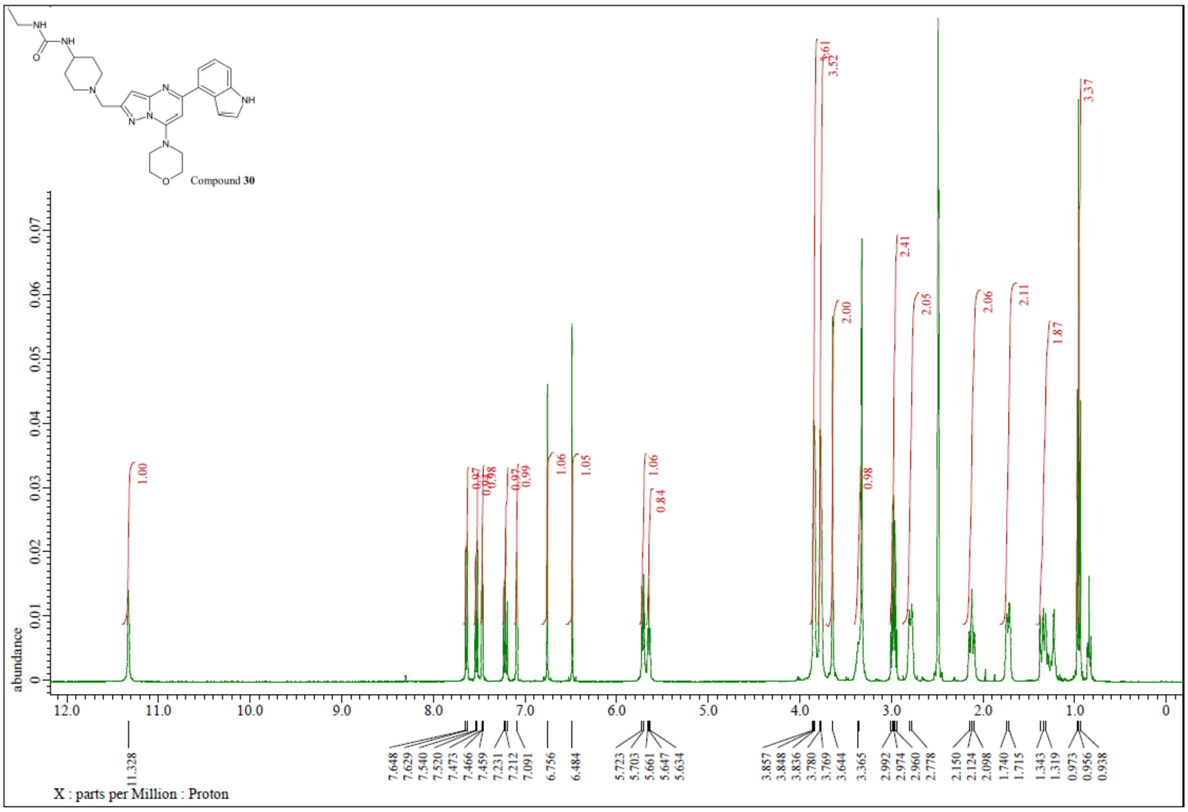
Compound 30
abundance
X : parts per Million : Proton

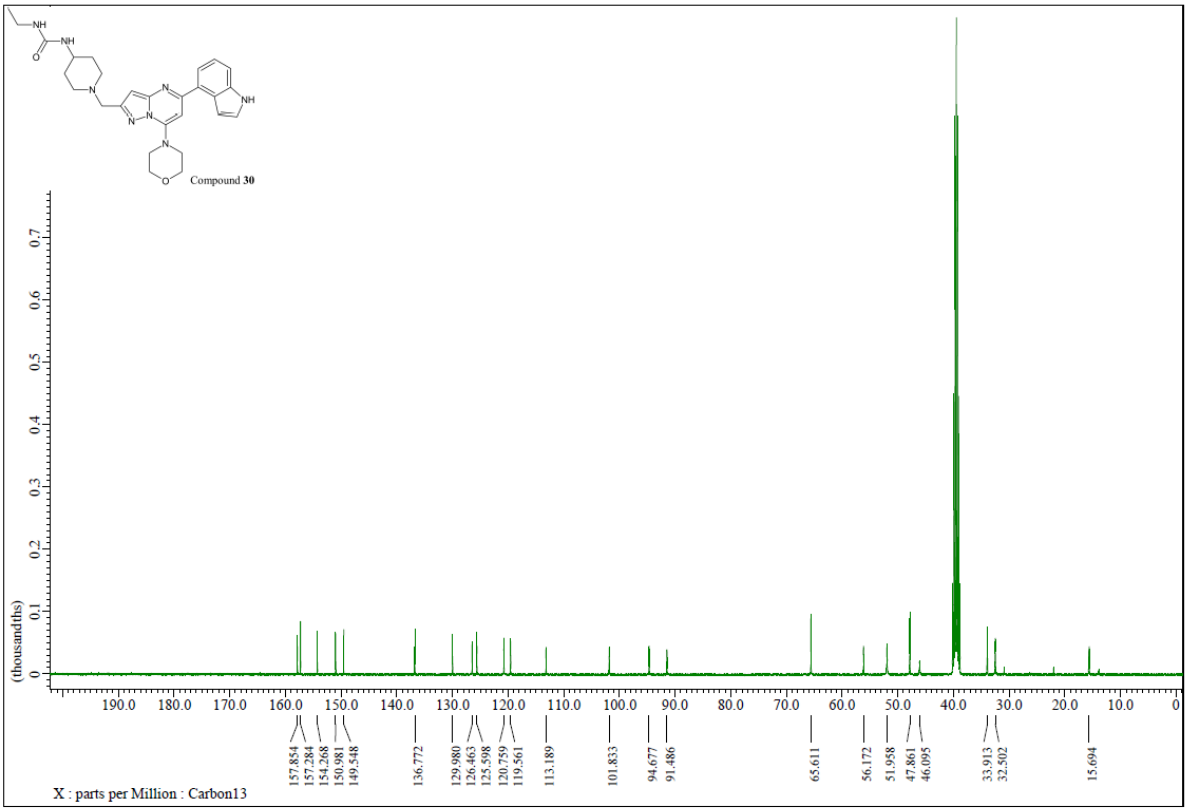
Compound 30
(thousandths)
X : parts per Million : Carbon13

Compound 30

Name	*Obs. m/z*	Obs. RT	Obs. Mass	Tgt Formula	Tgt Mass	Tgt Mass Error	Find Cpds Algorith
Compound **30**	503.28816	1.12	502.28111	C27 H34 N8 O2	502.28047	1.26	Find by Formula

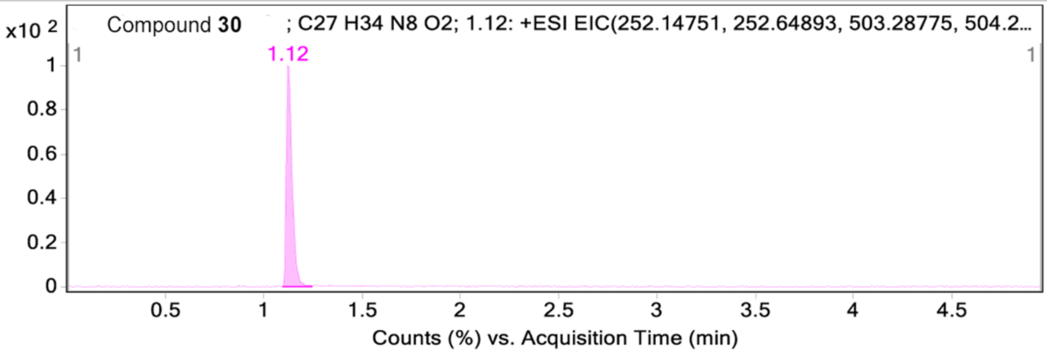

MS Zoomed Spectrum

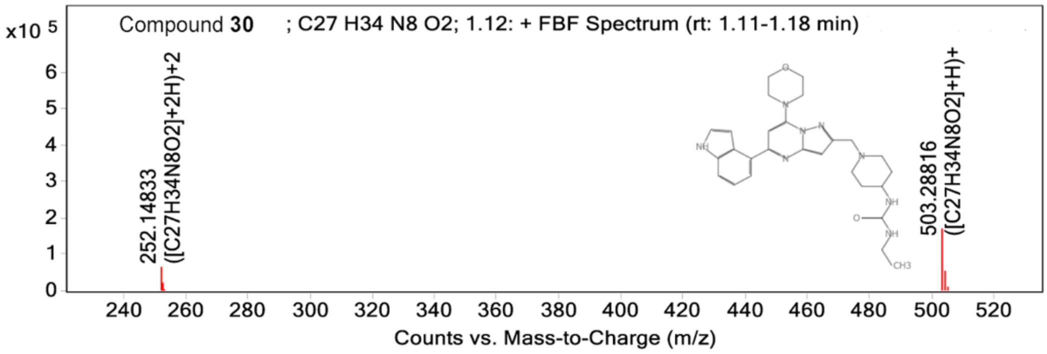

MS Zoomed Spectrum

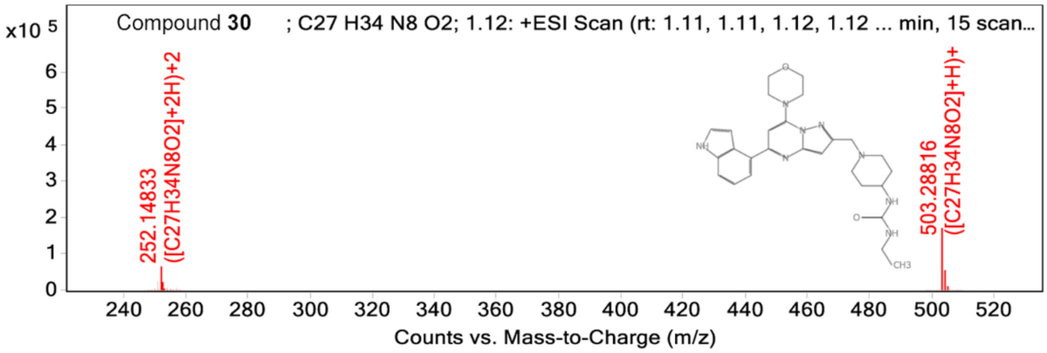

MS Spectrum Peak List

Obs. m/z	Charge	Abund	Formula	Ion/Isotope	Tgt Mass Error (ppm)
252.14833	2	64928.3	C27H34N8O2	(M+2H)+2	
252.64947	2	21703.73	C27H34N8O2	(M+2H)+2	
253.14989	2	4265.57	C27H34N8O2	(M+2H)+2	
253.6516	2	378.26	C27H34N8O2	(M+2H)+2	
503.28816	1	170039.05	C27H34N8O2	(M+H)+	
504.29073	1	51172.18	C27H34N8O2	(M+H)+	
505.29311	1	8059.46	C27H34N8O2	(M+H)+	
252.14833	2	64928.3	C27H34N8O2	(M+2H)+2	3.26
252.64947	2	21703.73	C27H34N8O2	(M+2H)+2	2.17
253.14989	2	4265.57	C27H34N8O2	(M+2H)+2	-1.55
253.6516	2	378.26	C27H34N8O2	(M+2H)+2	0.07
503.28816	1	170039.05	C27H34N8O2	(M+H)+	0.81
504.29073	1	51172.18	C27H34N8O2	(M+H)+	0.3
505.29311	1	8059.46	C27H34N8O2	(M+H)+	-0.34

--- End Of Report ---

Compound 31

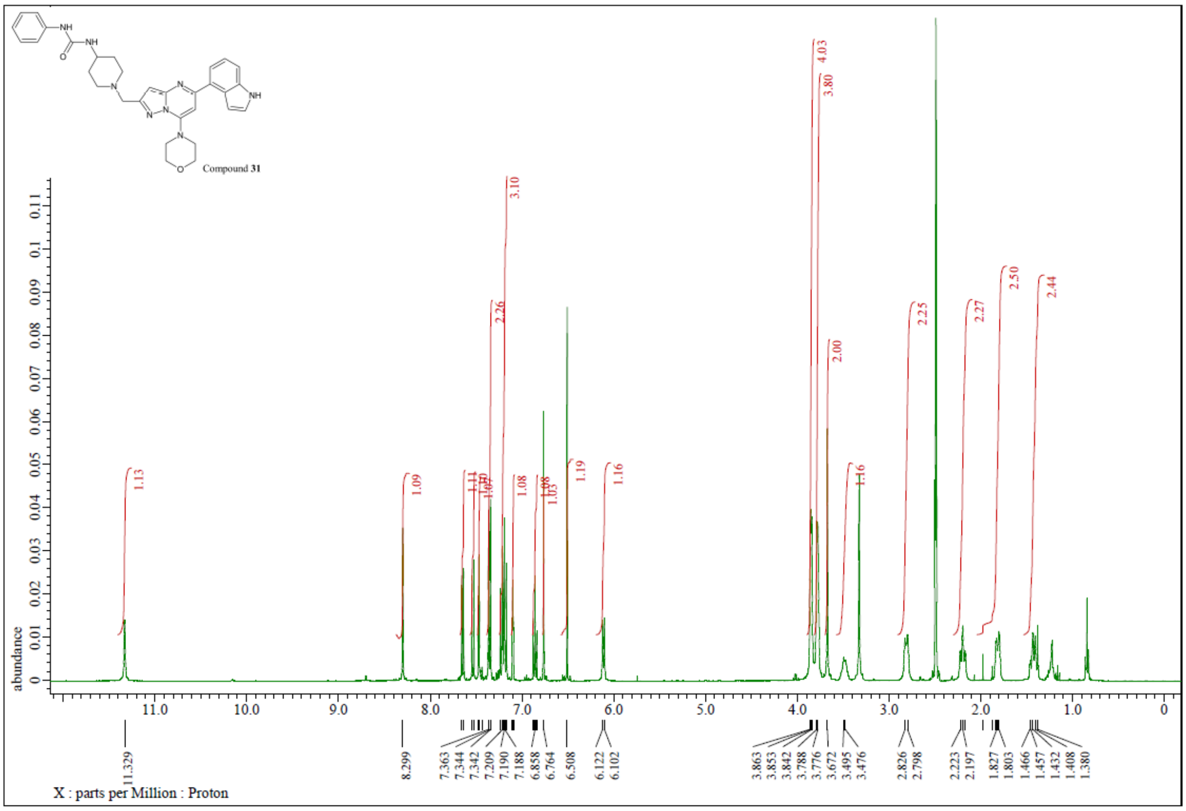

Compound 31
abundance
X : parts per Million : Proton

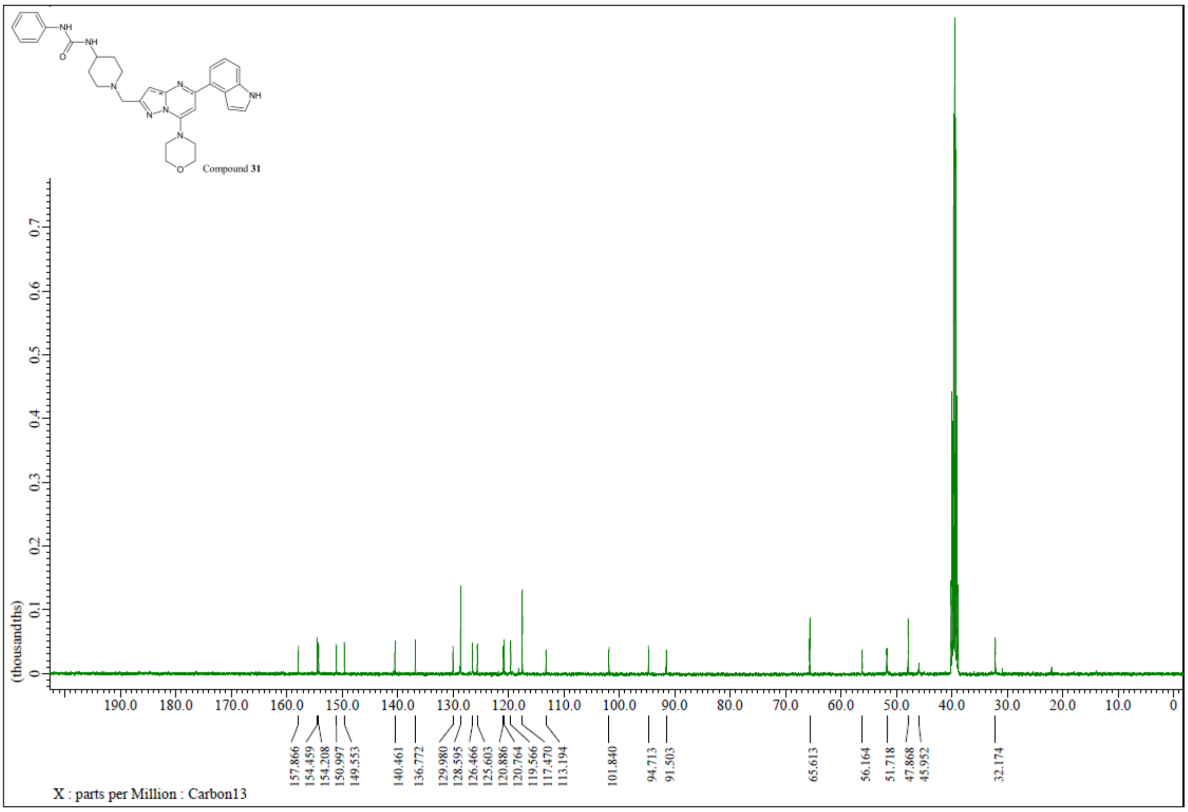

Compound 31
(thousandths)
X : parts per Million : Carbon13

Compound 31

Name	*Obs. m/z*	Obs. RT	Obs. Mass	Tgt Formula	Tgt Mass	Tgt Mass Error	Find Cpds Algorith
Compound **31**	551.28797	1.37	550.28084	C31 H34 N8 O2	550.28047	0.66	Find by Formula

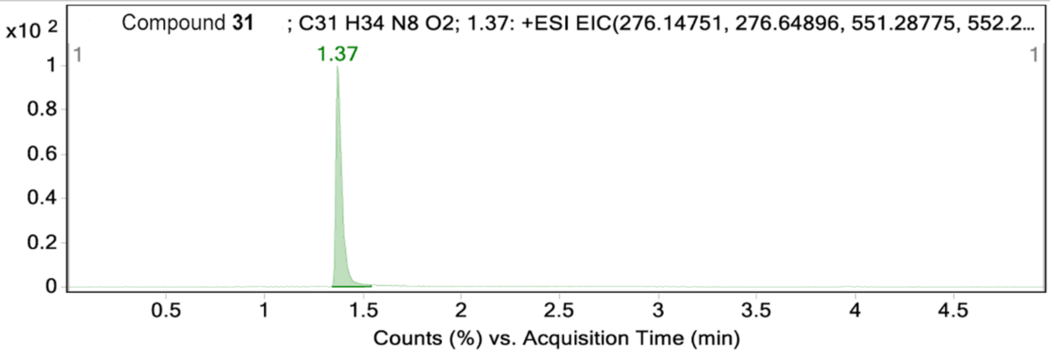

MS Zoomed Spectrum

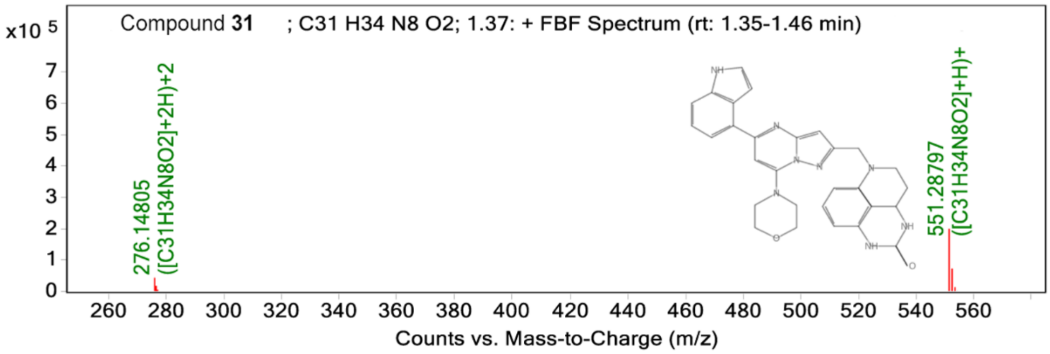

MS Zoomed Spectrum

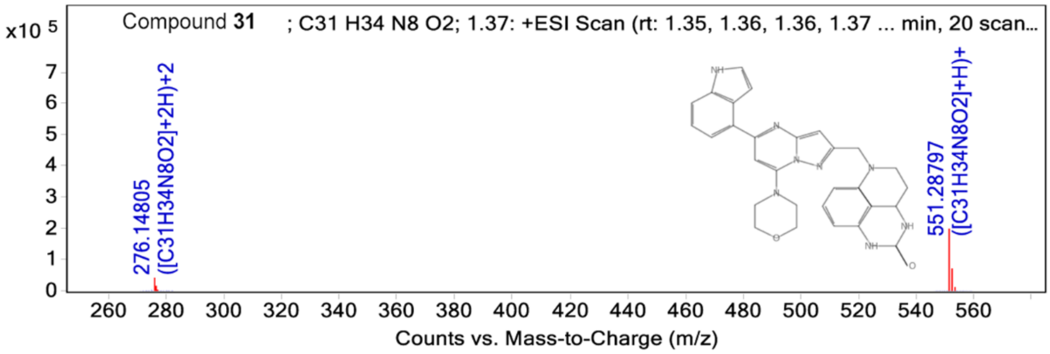

MS Spectrum Peak List

Obs. m/z	Charge	Abund	Formula	Ion/Isotope	Tgt Mass Error (ppm)
276.14805	2	40949.66	C31H34N8O2	(M+2H)+2	
276.64942	2	15629.86	C31H34N8O2	(M+2H)+2	
277.15073	2	3435.34	C31H34N8O2	(M+2H)+2	
277.65147	2	351.9	C31H34N8O2	(M+2H)+2	
551.28797	1	197852.61	C31H34N8O2	(M+H)+	
552.29099	1	69000.93	C31H34N8O2	(M+H)+	
553.29311	1	12470.17	C31H34N8O2	(M+H)+	
554.29584	1	1658.46	C31H34N8O2	(M+H)+	
276.14805	2	40949.66	C31H34N8O2	(M+2H)+2	1.94
276.64942	2	15629.86	C31H34N8O2	(M+2H)+2	1.67
277.15073	2	3435.34	C31H34N8O2	(M+2H)+2	1.39
277.65147	2	351.9	C31H34N8O2	(M+2H)+2	-0.83
551.28797	1	197852.61	C31H34N8O2	(M+H)+	0.4
552.29099	1	69000.93	C31H34N8O2	(M+H)+	0.64
553.29311	1	12470.17	C31H34N8O2	(M+H)+	-0.57
554.29584	1	1658.46	C31H34N8O2	(M+H)+	-0.52

--- End Of Report ---

Compound 32

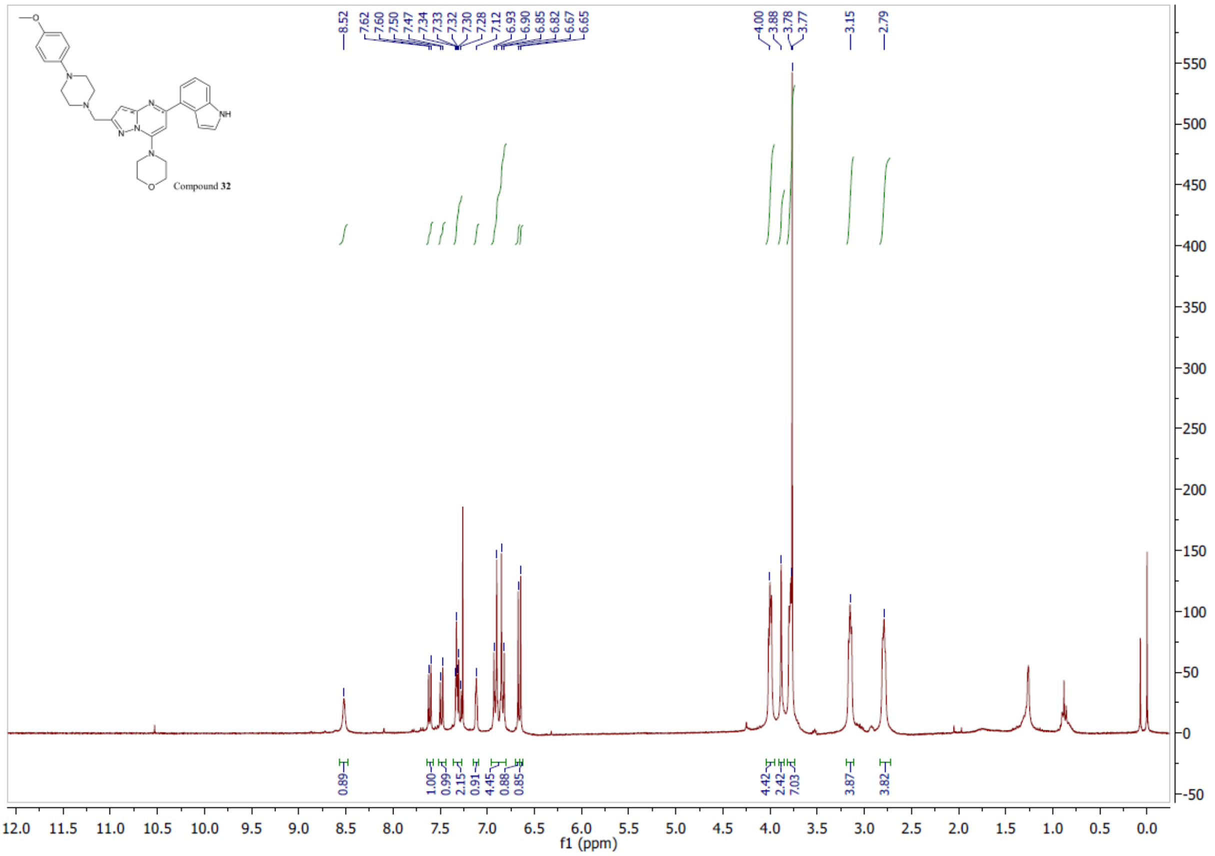

Compound 32
8.52
7.62
7.60
7.50
7.47
7.34
7.33
7.32
7.30
7.28
7.12
6.93
6.90
6.85
6.82
6.67
6.65
4.00
3.88
3.78
3.77
3.15
2.79
0.89
1.00
0.99
2.15
0.91
4.45
0.88
0.85
4.42
2.42
7.03
3.87
3.82
f1 (ppm)

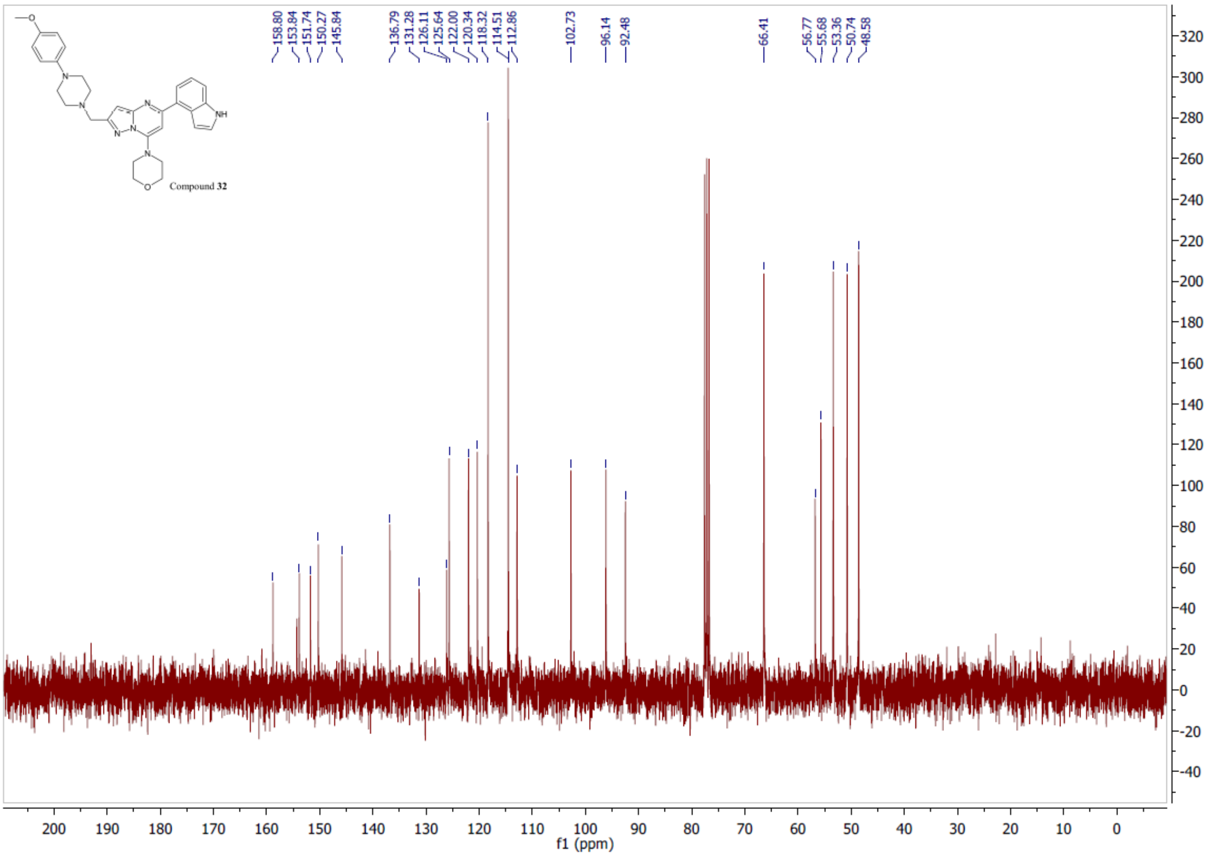

Compound 32
158.80
153.84
151.74
150.27
145.84
136.79
131.28
126.11
125.64
122.00
120.34
118.32
114.51
112.86
102.73
96.14
92.48
66.41
56.77
55.68
53.36
50.74
48.58
f1 (ppm)

Compound 32

Name	*Obs. m/z*	Obs. RT	Obs. Mass	Tgt Formula	Tgt Mass	Tgt Mass Error	Find Cpds Algorith
Compound **32**	524.27699	1.45	523.26996	C30 H33 N7 O2	523.26957	0.75	Find by Formula

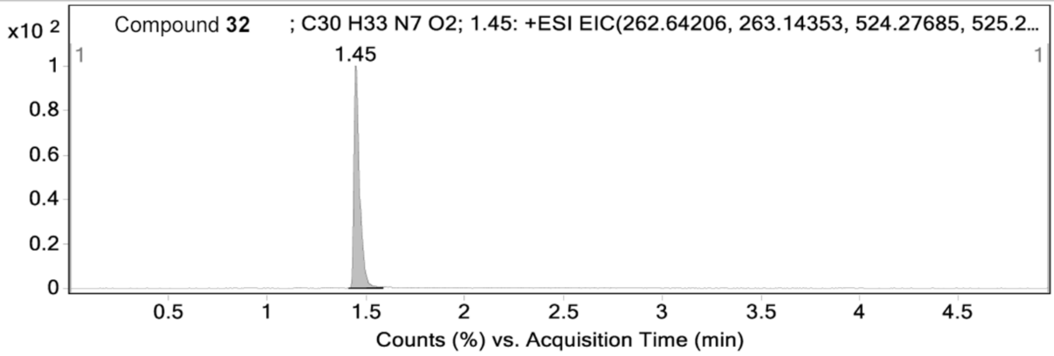

MS Zoomed Spectrum

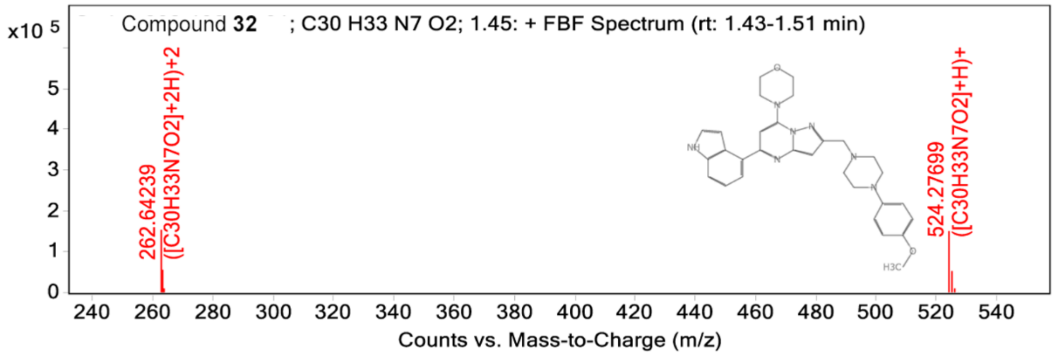

MS Zoomed Spectrum

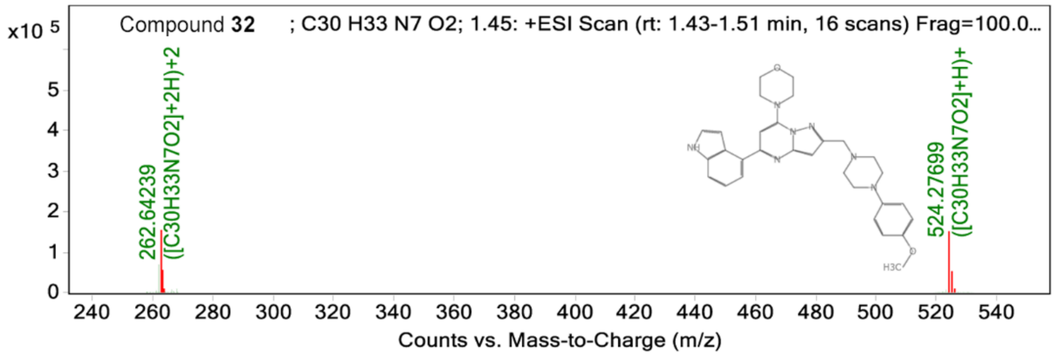

MS Spectrum Peak List

Obs. m/z	Charge	Abund	Formula	Ion/Isotope	Tgt Mass Error (ppm)
262.64239	2	153431.98	C30H33N7O2	(M+2H)+2	
263.14403	2	49281.49	C30H33N7O2	(M+2H)+2	
263.64525	2	8827.74	C30H33N7O2	(M+2H)+2	
524.27699	1	149768.55	C30H33N7O2	(M+H)+	
525.27966	1	50773.94	C30H33N7O2	(M+H)+	
526.28195	1	8617.05	C30H33N7O2	(M+H)+	
527.28537	1	1117.81	C30H33N7O2	(M+H)+	
262.64239	2	153431.98	C30H33N7O2	(M+2H)+2	1.25
263.14403	2	49281.49	C30H33N7O2	(M+2H)+2	1.89
263.64525	2	8827.74	C30H33N7O2	(M+2H)+2	1.18
524.27699	1	149768.55	C30H33N7O2	(M+H)+	0.28
525.27966	1	50773.94	C30H33N7O2	(M+H)+	-0.24
526.28195	1	8617.05	C30H33N7O2	(M+H)+	-1.24
527.28537	1	1117.81	C30H33N7O2	(M+H)+	0.08

--- End Of Report ---

Compound 33

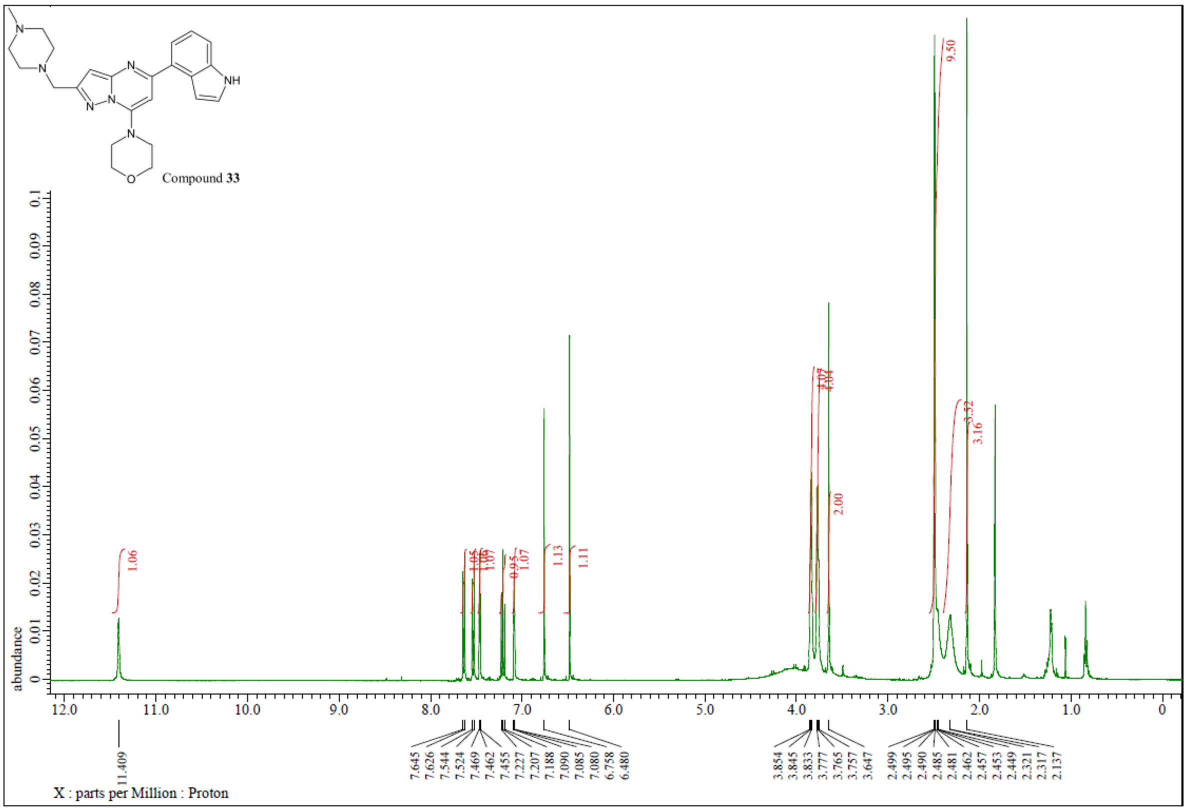

Compound 33
abundance
X : parts per Million : Proton

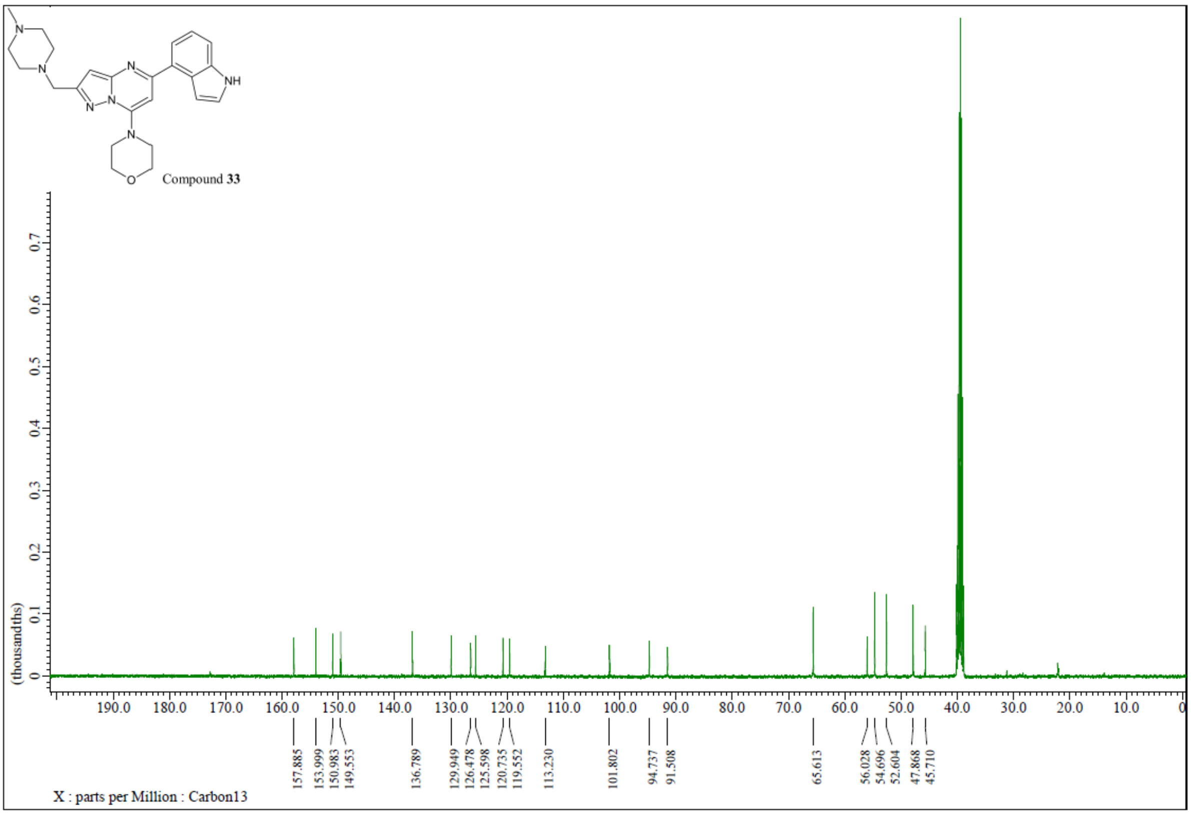

Compound 33
(thousandths)
X : parts per Million : Carbon13

Compound 33

Name	*Obs. m/z*	Obs. RT	Obs. Mass	Tgt Formula	Tgt Mass	Tgt Mass Error	Find Cpds Algorith
Compound **33**	432.25113	0.98	431.24397	C24 H29 N7 O	431.24336	1.43	Find by Formula

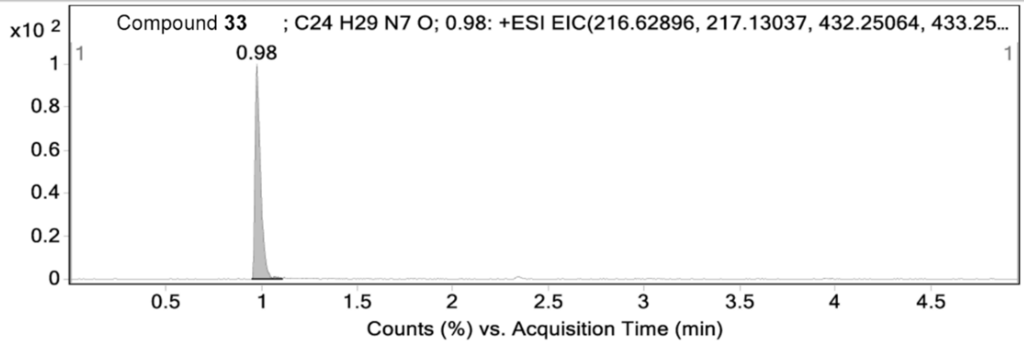

MS Zoomed Spectrum

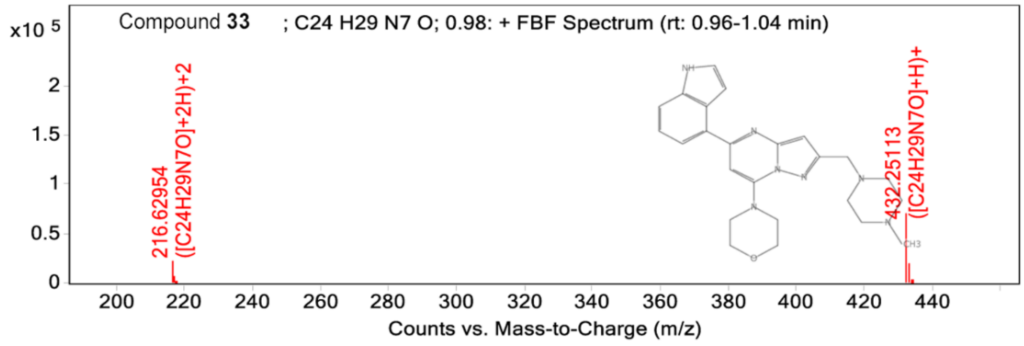

MS Zoomed Spectrum

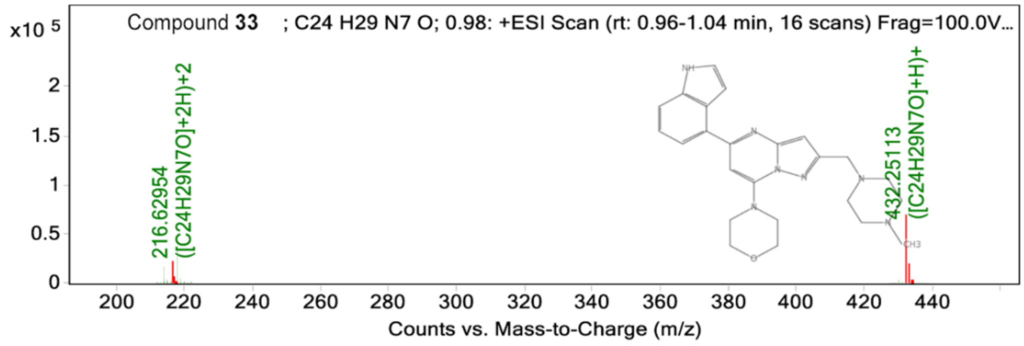

MS Spectrum Peak List

Obs. m/z	Charge	Abund	Formula	Ion/Isotope	Tgt Mass Error (ppm)
216.62954	2	21711.37	C24H29N7O	(M+2H)+2	
217.13078	2	6384.77	C24H29N7O	(M+2H)+2	
217.63214	2	939.34	C24H29N7O	(M+2H)+2	
432.25113	1	69948.29	C24H29N7O	(M+H)+	
433.25391	1	19650.1	C24H29N7O	(M+H)+	
434.2564	1	2730.67	C24H29N7O	(M+H)+	
435.25842	1	318.43	C24H29N7O	(M+H)+	
216.62954	2	21711.37	C24H29N7O	(M+2H)+2	2.7
217.13078	2	6384.77	C24H29N7O	(M+2H)+2	1.86
217.63214	2	939.34	C24H29N7O	(M+2H)+2	1.83
432.25113	1	69948.29	C24H29N7O	(M+H)+	1.14
433.25391	1	19650.1	C24H29N7O	(M+H)+	1.02
434.2564	1	2730.67	C24H29N7O	(M+H)+	0.44
435.25842	1	318.43	C24H29N7O	(M+H)+	-1.03

--- End Of Report ---

Compound 34

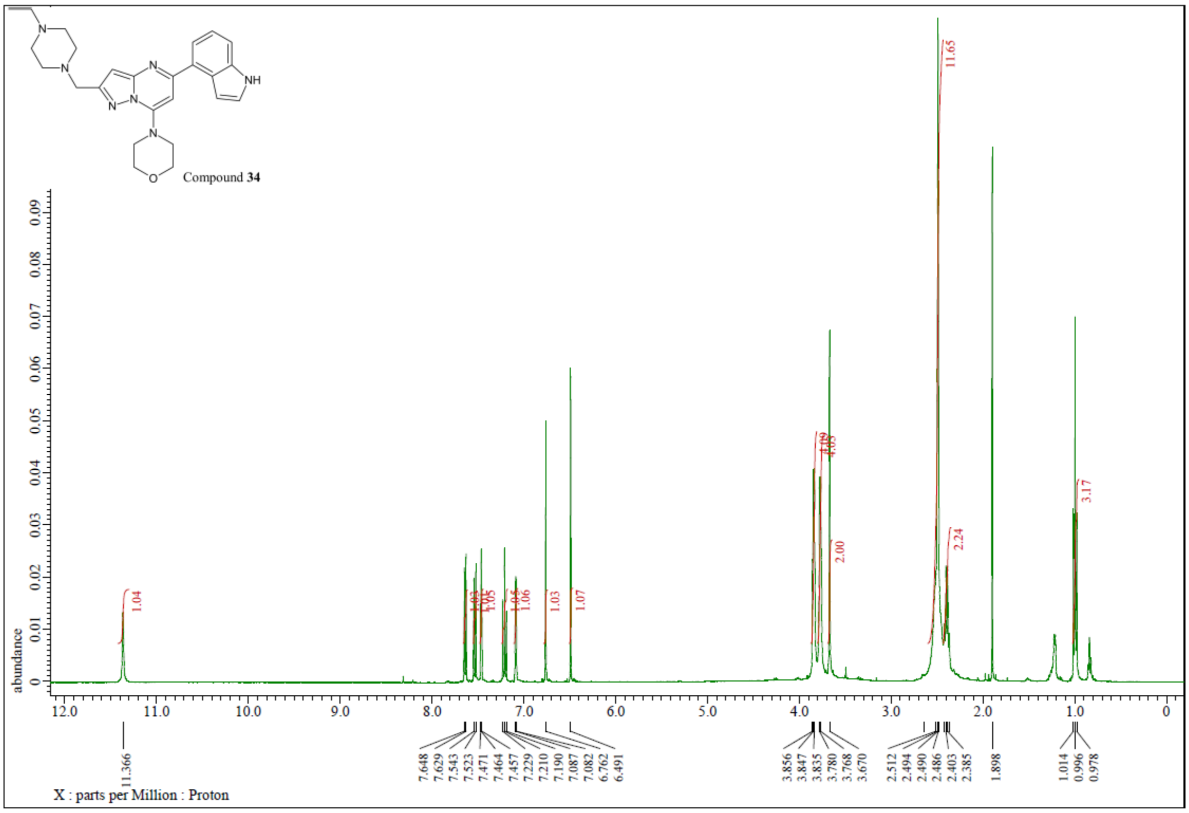

Compound 34
abundance
X : parts per Million : Proton

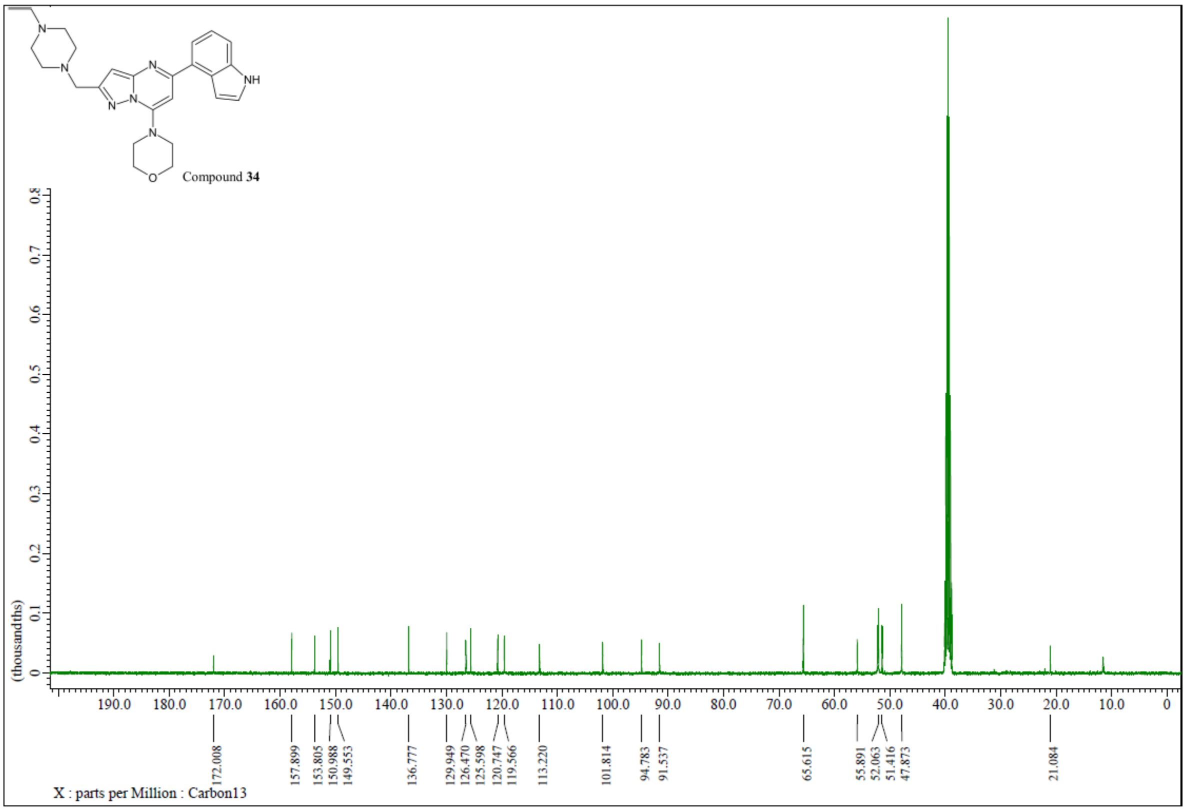

Compound 34
(thousandths)
X : parts per Million : Carbon13

Compound 34

Name	*Obs. m/z*	Obs. RT	Obs. Mass	Tgt Formula	Tgt Mass	Tgt Mass Error	Find Cpds Algorith
Compound **34**	446.26661	0.99	445.25954	C25 H31 N7 O	445.25901	1.19	Find by Formula

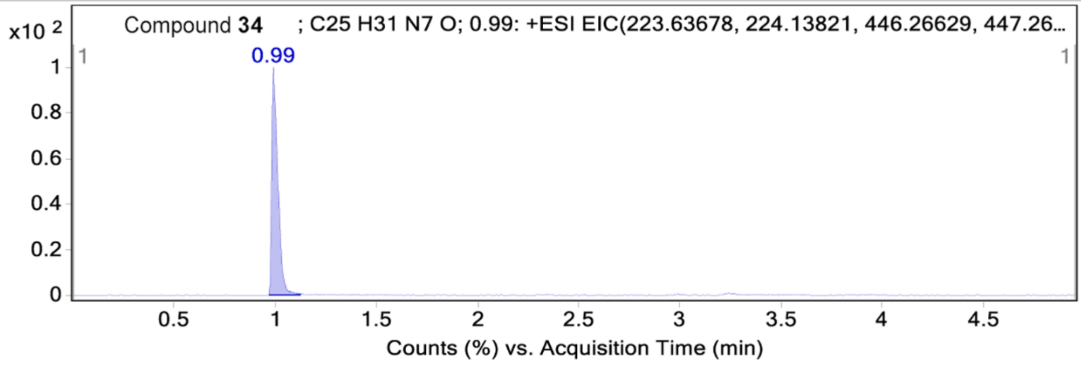

MS Zoomed Spectrum

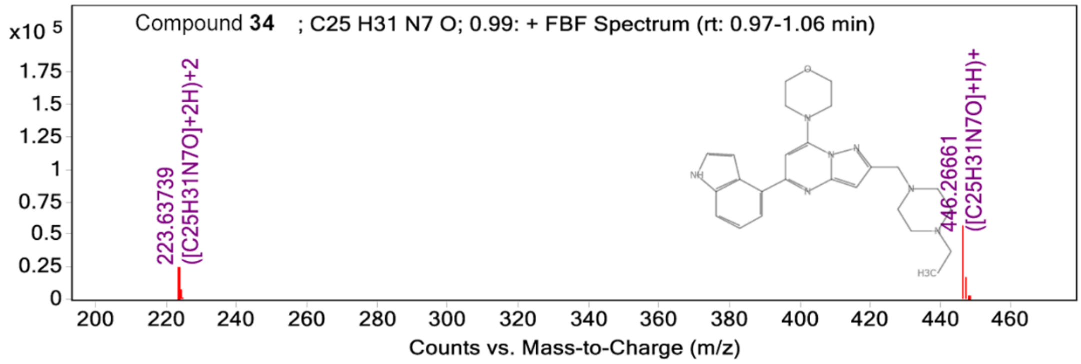

MS Zoomed Spectrum

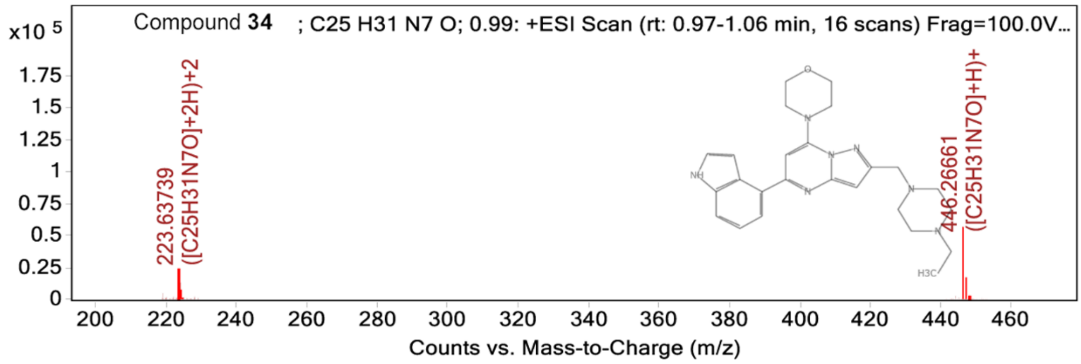

MS Spectrum Peak List

Obs. m/z	Charge	Abund	Formula	Ion/Isotope	Tgt Mass Error (ppm)
223.63739	2	23763.25	C25H31N7O	(M+2H)+2	
224.6402	2	1095.98	C25H31N7O	(M+2H)+2	
446.26661	1	56774.48	C25H31N7O	(M+H)+	
447.26924	1	15807.67	C25H31N7O	(M+H)+	
448.27271	1	2984.79	C25H31N7O	(M+H)+	
223.63739	2	23763.25	C25H31N7O	(M+2H)+2	2.74
224.6402	2	1095.98	C25H31N7O	(M+2H)+2	2.7
446.26661	1	56774.48	C25H31N7O	(M+H)+	0.74
447.26924	1	15807.67	C25H31N7O	(M+H)+	0.22
448.27271	1	2984.79	C25H31N7O	(M+H)+	1.8

--- End Of Report ---

Compound 35

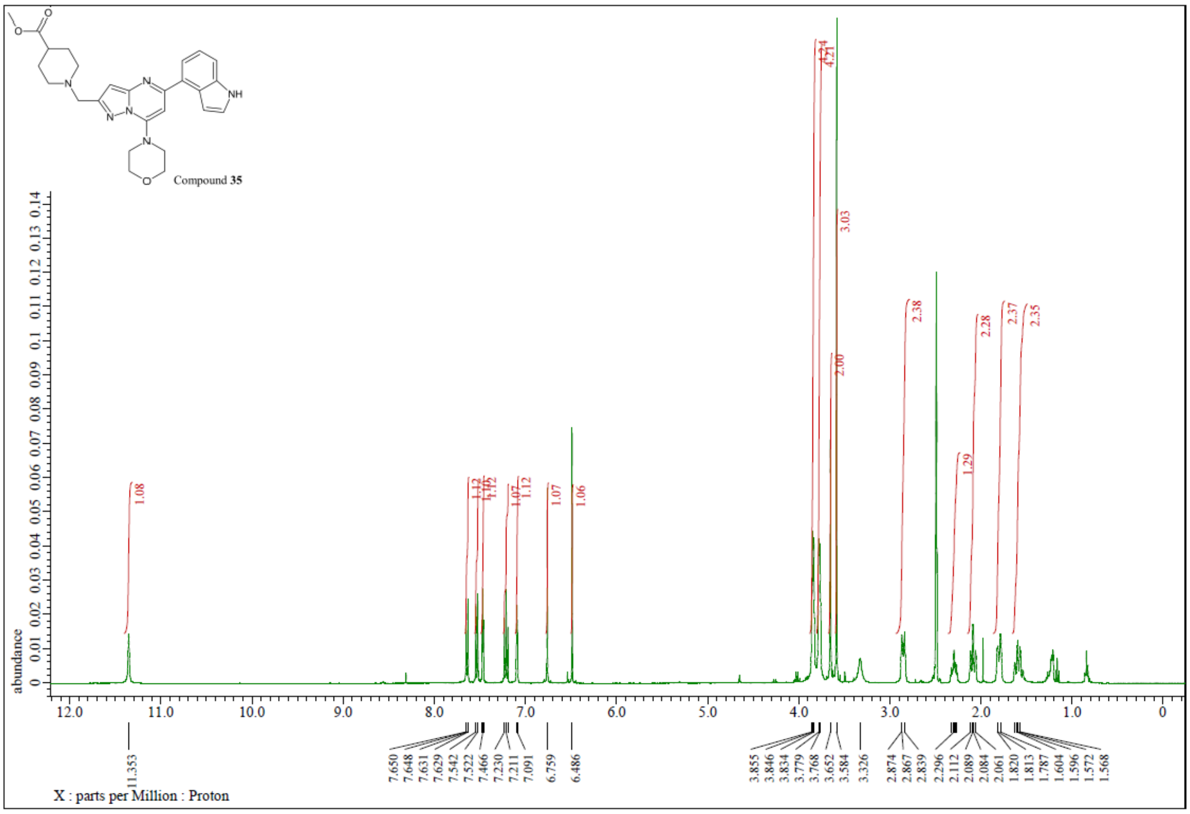
NH
N
O
Compound 35
abundance
X : parts per Million : Proton
11.353
7.650
7.648
7.631
7.629
7.542
7.522
7.466
7.230
7.211
7.091
6.759
6.486
3.855
3.846
3.834
3.779
3.768
3.652
3.584
3.326
2.874
2.867
2.839
2.296
2.112
2.089
2.084
2.061
1.820
1.813
1.787
1.604
1.596
1.572
1.568

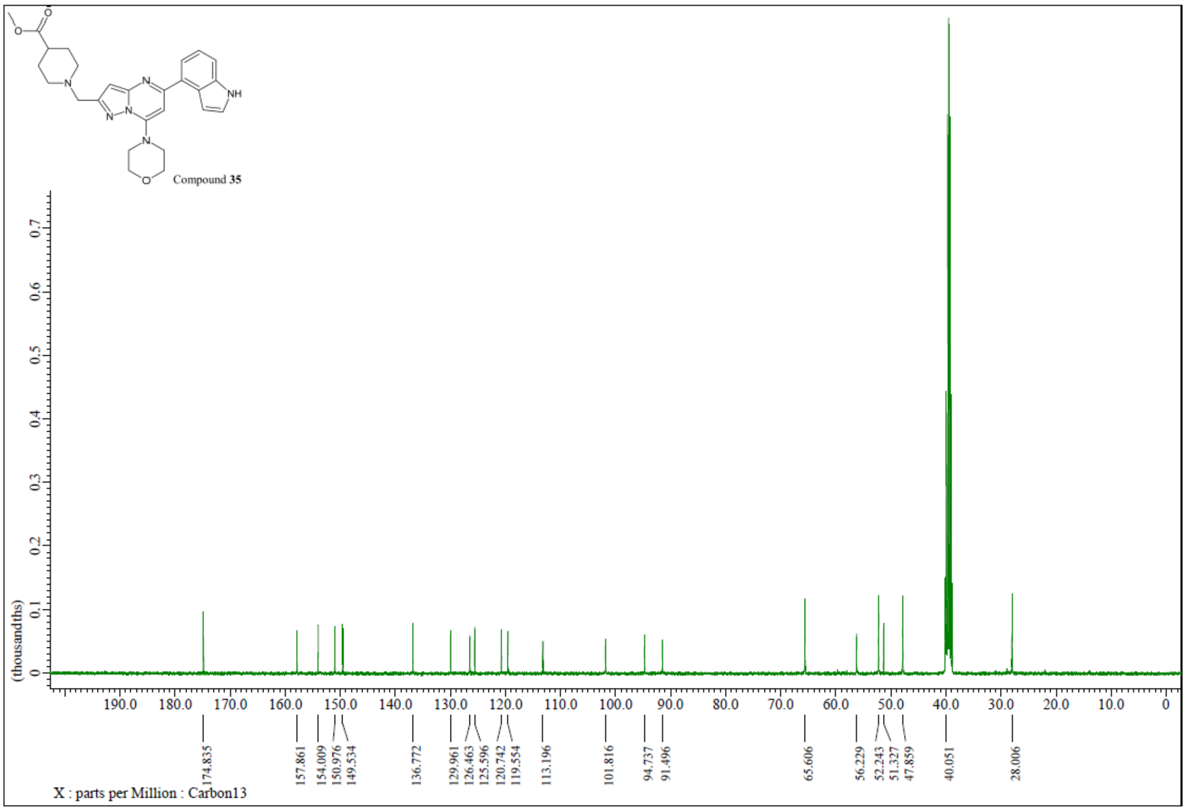
NH
N
O
Compound 35
(thousandths)
X : parts per Million : Carbon13
174.835
157.861
154.009
150.976
149.534
136.772
129.961
126.463
125.596
120.742
119.554
113.196
101.816
94.737
91.496
65.606
56.229
52.243
51.327
47.859
40.051
28.006

Compound 35

Name	*Obs. m/z*	Obs. RT	Obs. Mass	Tgt Formula	Tgt Mass	Tgt Mass Error	Find Cpds Algorith
Compound **35**	475.24578	1.18	474.23875	C26 H30 N6 O3	474.23794	1.71	Find by Formula

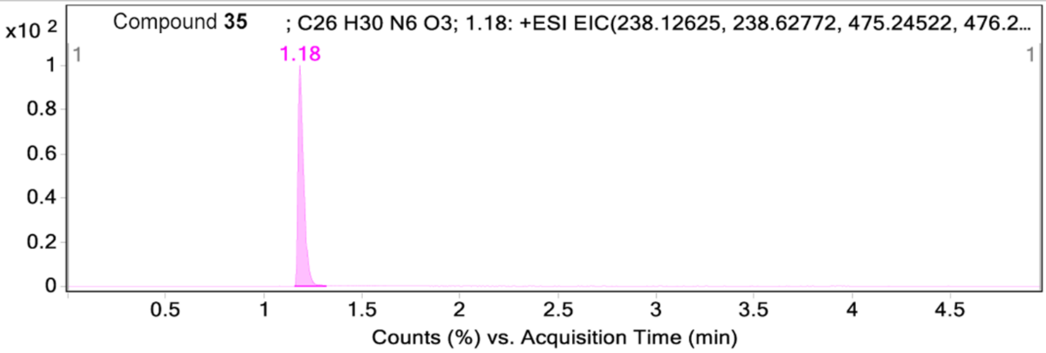

MS Zoomed Spectrum

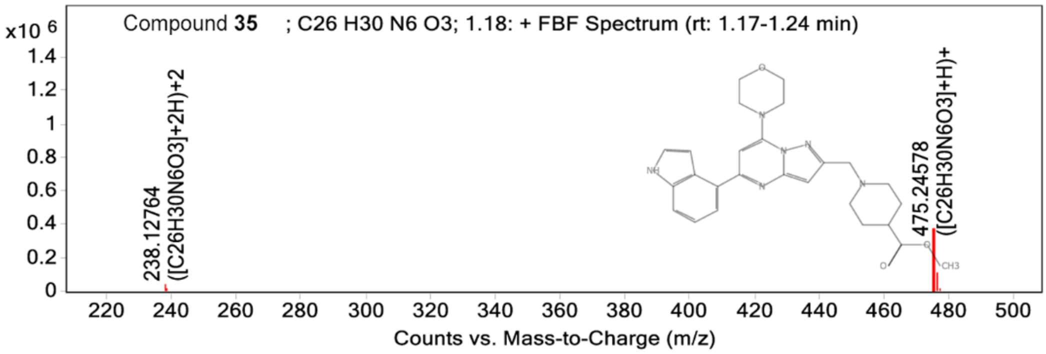

MS Zoomed Spectrum

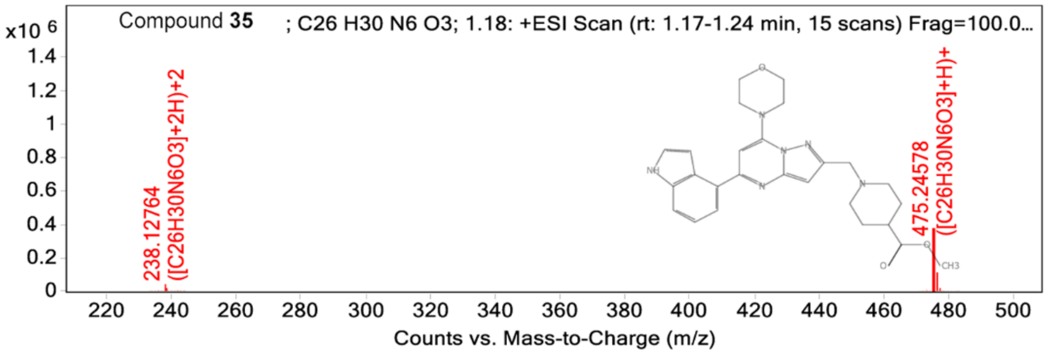

MS Spectrum Peak List

Obs. m/z	Charge	Abund	Formula	Ion/Isotope	Tgt Mass Error (ppm)
238.12764	2	39287.81	C26H30N6O3	(M+2H)+2	
238.62889	2	12270.96	C26H30N6O3	(M+2H)+2	
239.13084	2	1943.22	C26H30N6O3	(M+2H)+2	
239.62934	2	274.5	C26H30N6O3	(M+2H)+2	
475.24578	1	370502.28	C26H30N6O3	(M+H)+	
476.24896	1	106459.38	C26H30N6O3	(M+H)+	
477.25109	1	17094.23	C26H30N6O3	(M+H)+	
478.25404	1	2155.16	C26H30N6O3	(M+H)+	
238.12764	2	39287.81	C26H30N6O3	(M+2H)+2	5.87
238.62889	2	12270.96	C26H30N6O3	(M+2H)+2	4.91
239.13084	2	1943.22	C26H30N6O3	(M+2H)+2	7.36
239.62934	2	274.5	C26H30N6O3	(M+2H)+2	-4.42
475.24578	1	370502.28	C26H30N6O3	(M+H)+	1.19
476.24896	1	106459.38	C26H30N6O3	(M+H)+	1.69
477.25109	1	17094.23	C26H30N6O3	(M+H)+	0.41
478.25404	1	2155.16	C26H30N6O3	(M+H)+	1.11

--- End Of Report ---

Compound 36

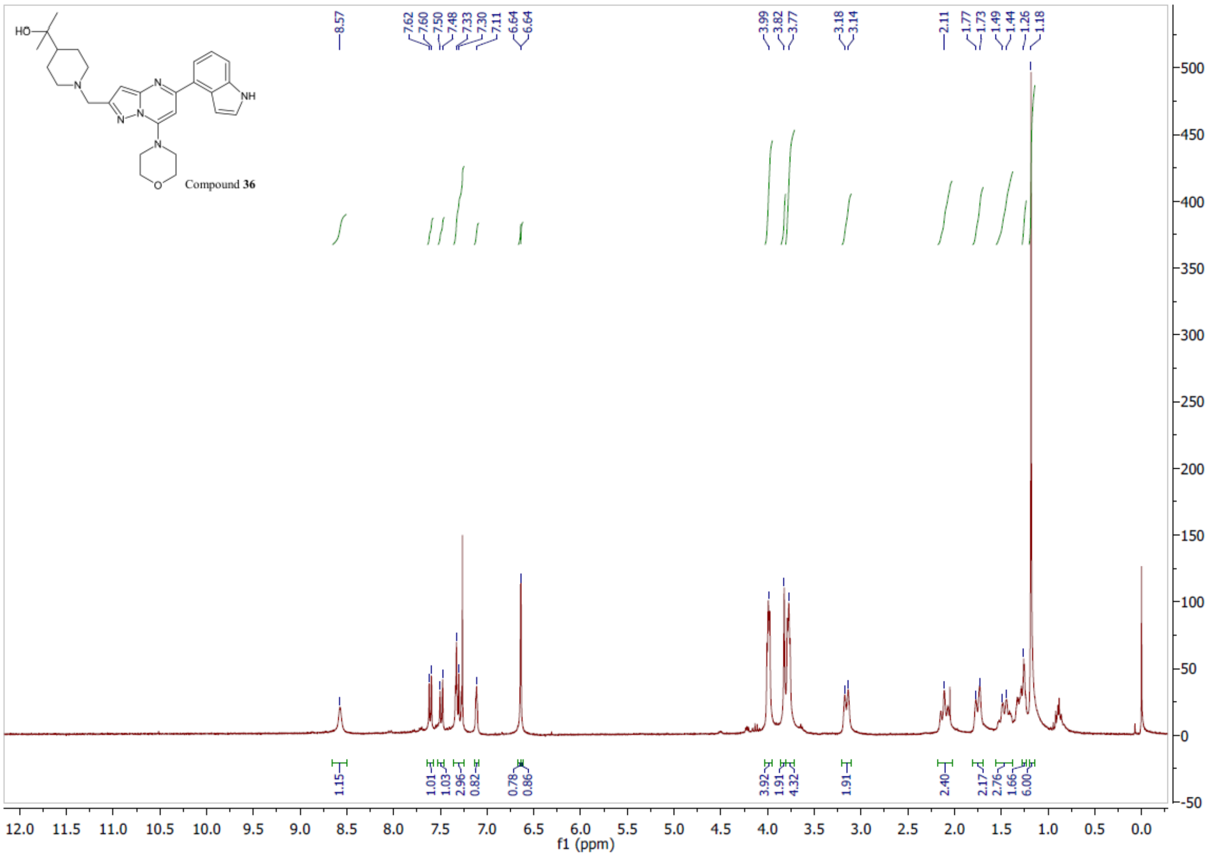

HO
N
N
N
N
NH
N
O
Compound 36
8.57
7.62
7.60
7.50
7.48
7.33
7.30
7.11
6.64
6.64
3.99
3.82
3.77
3.18
3.14
2.11
1.77
1.73
1.49
1.44
1.26
1.18
1.15
1.01
1.03
2.96
0.82
0.78
0.86
3.92
1.91
4.32
1.91
2.40
2.17
2.76
1.66
6.00
f1 (ppm)

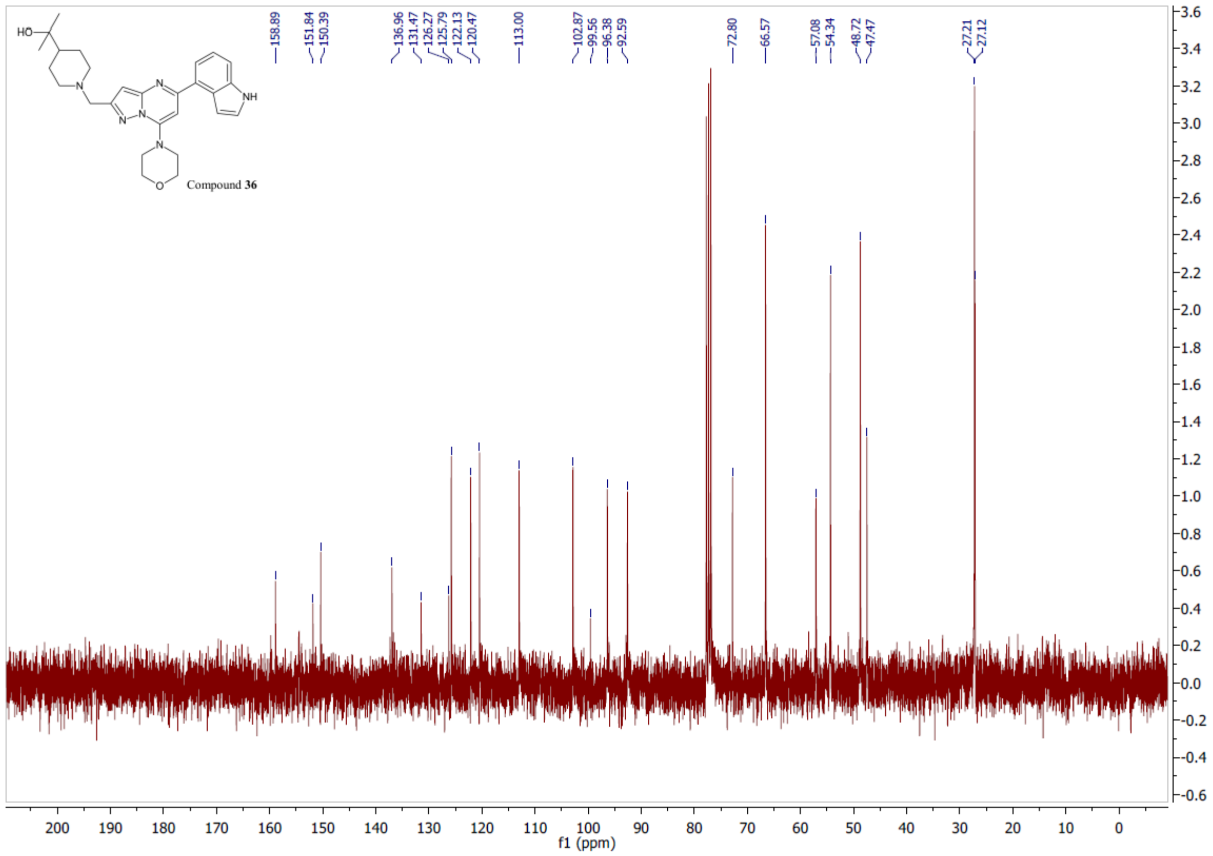

HO
N
N
N
N
NH
N
O
Compound 36
158.89
151.84
150.39
136.96
131.47
126.27
125.79
122.13
120.47
113.00
102.87
99.56
96.38
92.59
72.80
66.57
57.08
54.34
48.72
47.47
27.21
27.12
f1 (ppm)

Compound 36

Name	Obs. m/z	Obs. RT	Obs. Mass	Tgt Formula	Tgt Mass	Tgt Mass Error	Find Cpds Algorith
Compound **36**	475.2815	1.15	474.2752	C27 H34 N6 O2	474.2743	1.85	Find by Formula

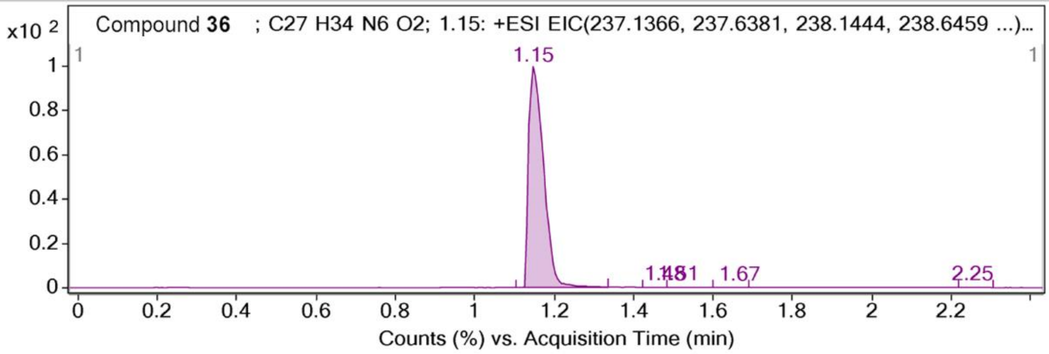

MS Zoomed Spectrum

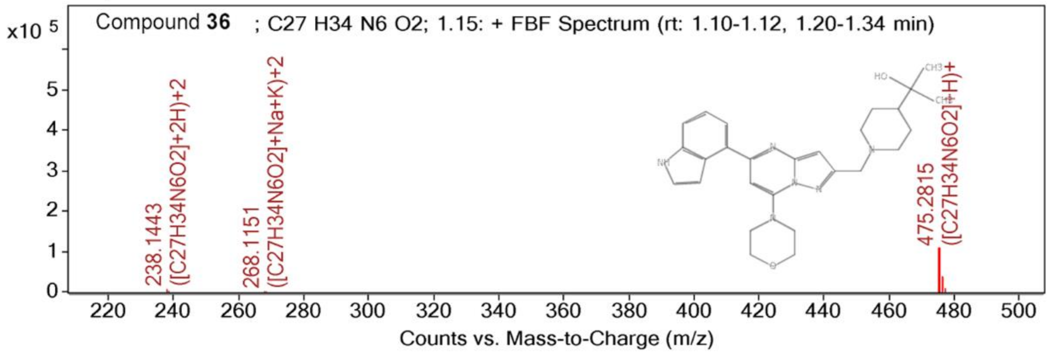

MS Zoomed Spectrum

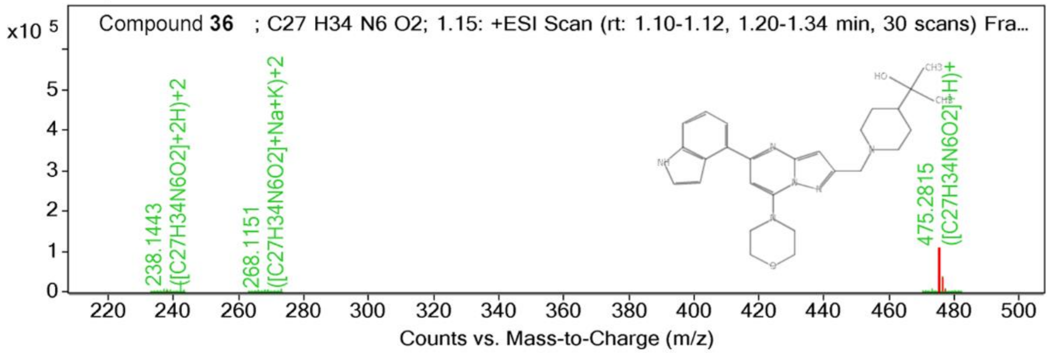

MS Spectrum Peak List

Obs. m/z	Charge	Abund	Formula	Ion/Isotope	Tgt Mass Error (ppm)
238.1443	2	4850.05	C27H34N6O2	(M+2H)+2	
238.6462	2	1098.49	C27H34N6O2	(M+2H)+2	
268.1151	2	173.99	C27H34N6O2	(M+Na+K)+2	
268.6137	2	18.68	C27H34N6O2	(M+Na+K)+2	
475.2815	1	104772.1	C27H34N6O2	(M+H)+	
476.288	1	36473.09	C27H34N6O2	(M+H)+	
477.29	1	7399.36	C27H34N6O2	(M+H)+	
238.1443	2	4850.05	C27H34N6O2	(M+2H)+2	-0.54
238.6462	2	1098.49	C27H34N6O2	(M+2H)+2	1.15
268.1151	2	173.99	C27H34N6O2	(M+Na+K)+2	6.37
268.6137	2	18.68	C27H34N6O2	(M+Na+K)+2	-4.18
475.2815	1	104772.1	C27H34N6O2	(M+H)+	-0.18
476.288	1	36473.09	C27H34N6O2	(M+H)+	7.21
477.29	1	7399.36	C27H34N6O2	(M+H)+	5.4

--- End Of Report ---

Compound 37

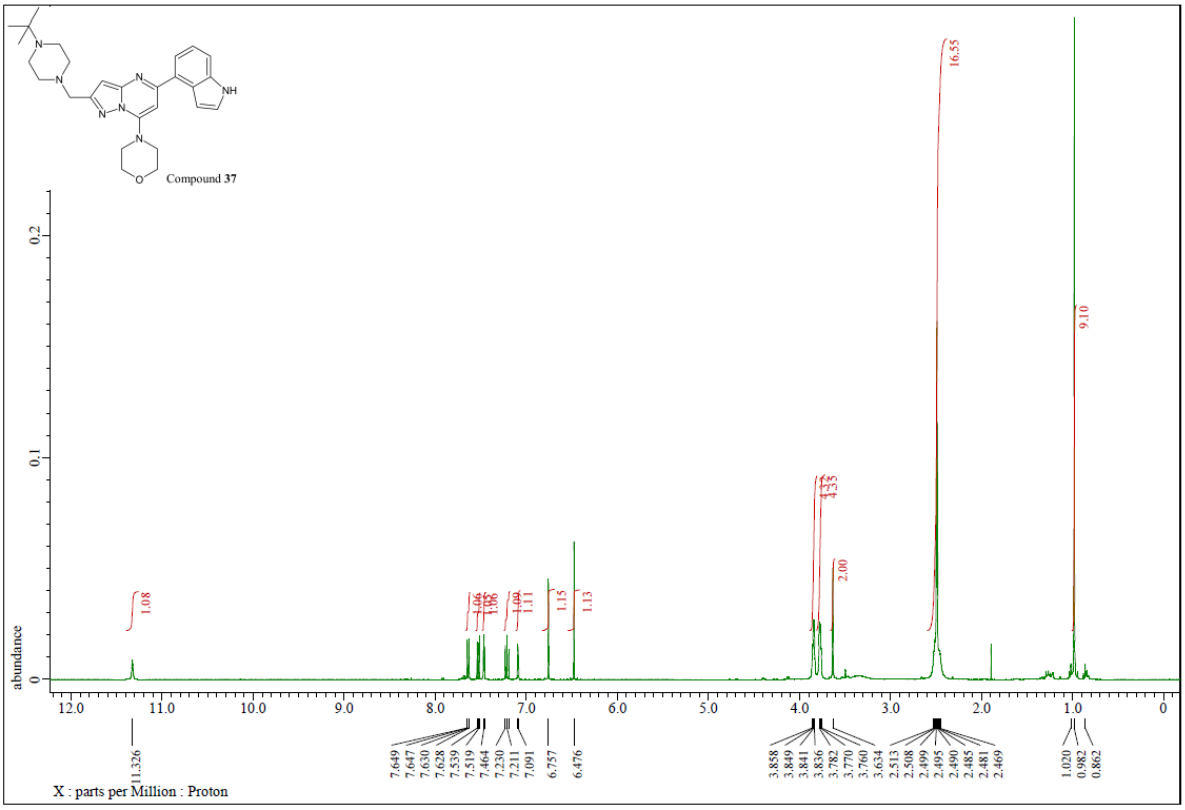

Compound 37
abundance
X : parts per Million : Proton

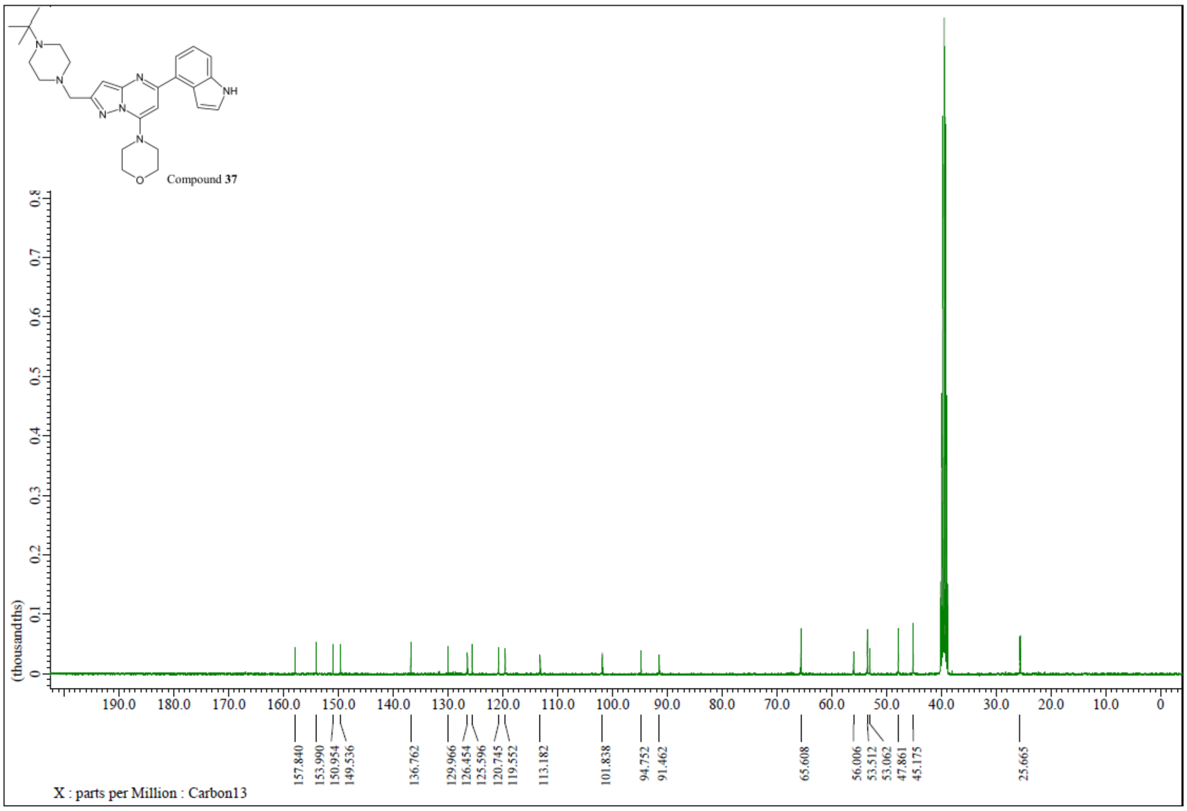

Compound 37
(thousandths)
X : parts per Million : Carbon13

Compound 37

Name	*Obs. m/z*	Obs. RT	Obs. Mass	Tgt Formula	Tgt Mass	Tgt Mass Error	Find Cpds Algorith
Compound **37**	474.2976	1.12	473.2904	C27 H35 N7 O	473.2903	0.1	Find by Formula

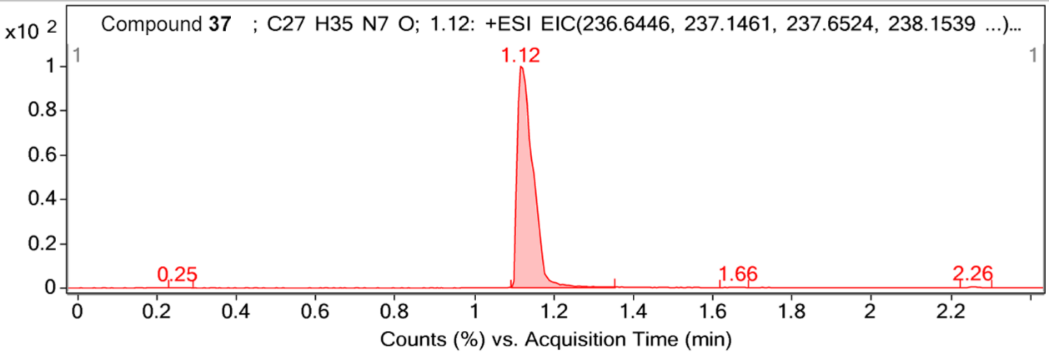

MS Zoomed Spectrum

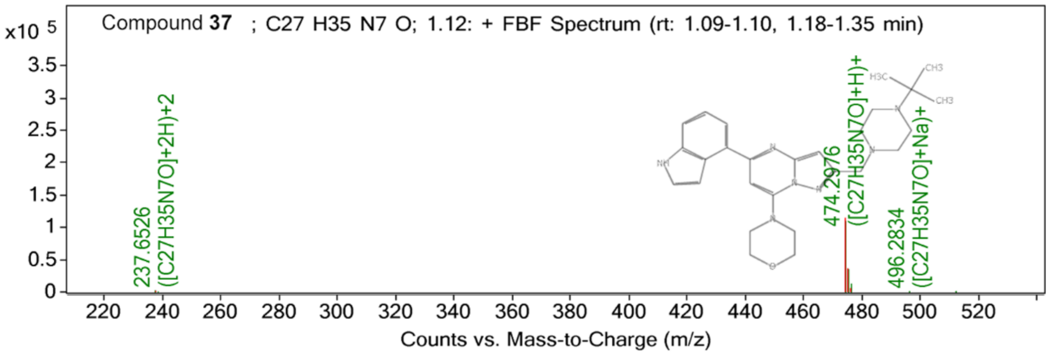

MS Zoomed Spectrum

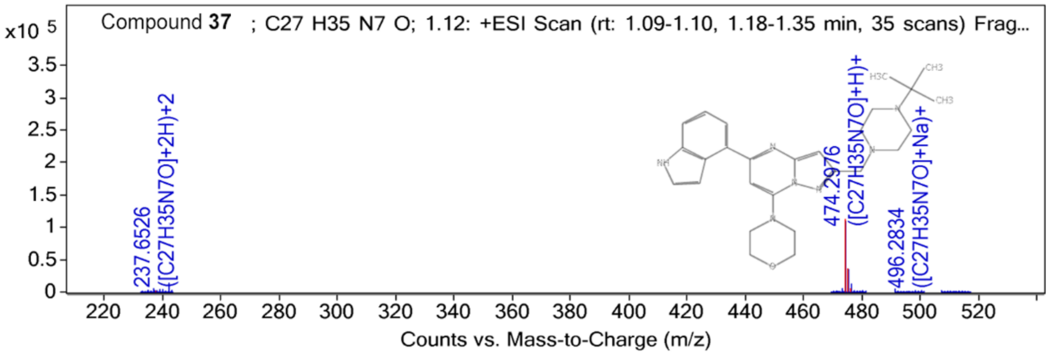

MS Spectrum Peak List

Obs. m/z	Charge	Abund	Formula	Ion/Isotope	Tgt Mass Error (ppm)
237.6526	2	1976.24	C27H35N7O	(M+2H)+2	
238.655	2	91.48	C27H35N7O	(M+2H)+2	
474.2976	1	109334.64	C27H35N7O	(M+H)+	
475.3002	1	34766.21	C27H35N7O	(M+H)+	
476.3044	1	12225.21	C27H35N7O	(M+H)+	
496.2834	1	461.95	C27H35N7O	(M+Na)+	
512.2544	1	408.88	C27H35N7O	(M+K)+	
237.6526	2	1976.24	C27H35N7O	(M+2H)+2	0.59
238.655	2	91.48	C27H35N7O	(M+2H)+2	-1
474.2976	1	109334.64	C27H35N7O	(M+H)+	0.02
475.3002	1	34766.21	C27H35N7O	(M+H)+	-0.58
476.3044	1	12225.21	C27H35N7O	(M+H)+	2.33
496.2834	1	461.95	C27H35N7O	(M+Na)+	7.8
512.2544	1	408.88	C27H35N7O	(M+K)+	1.88

--- End Of Report ---

Compound 38

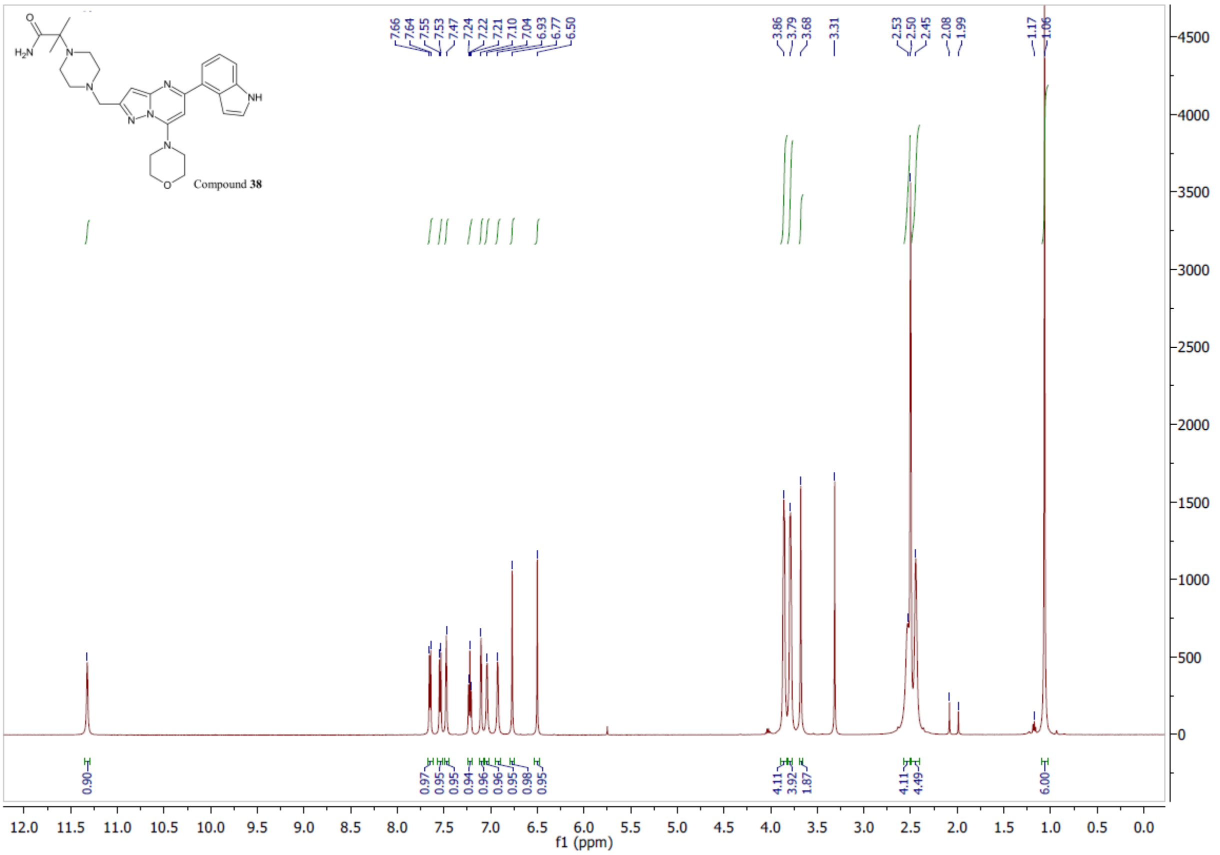
Compound 38
7.66 7.64 7.55 7.53 7.47 7.24 7.22 7.21 7.10 7.04 6.93 6.77 6.50
3.86 3.79 3.68 3.31 2.53 2.50 2.45 2.08 1.99 1.17 1.06
0.90 0.97 0.95 0.95 0.95 0.94 0.96 0.96 0.95 0.98 0.95 4.11 3.92 1.87 4.11 4.49 6.00
f1 (ppm)

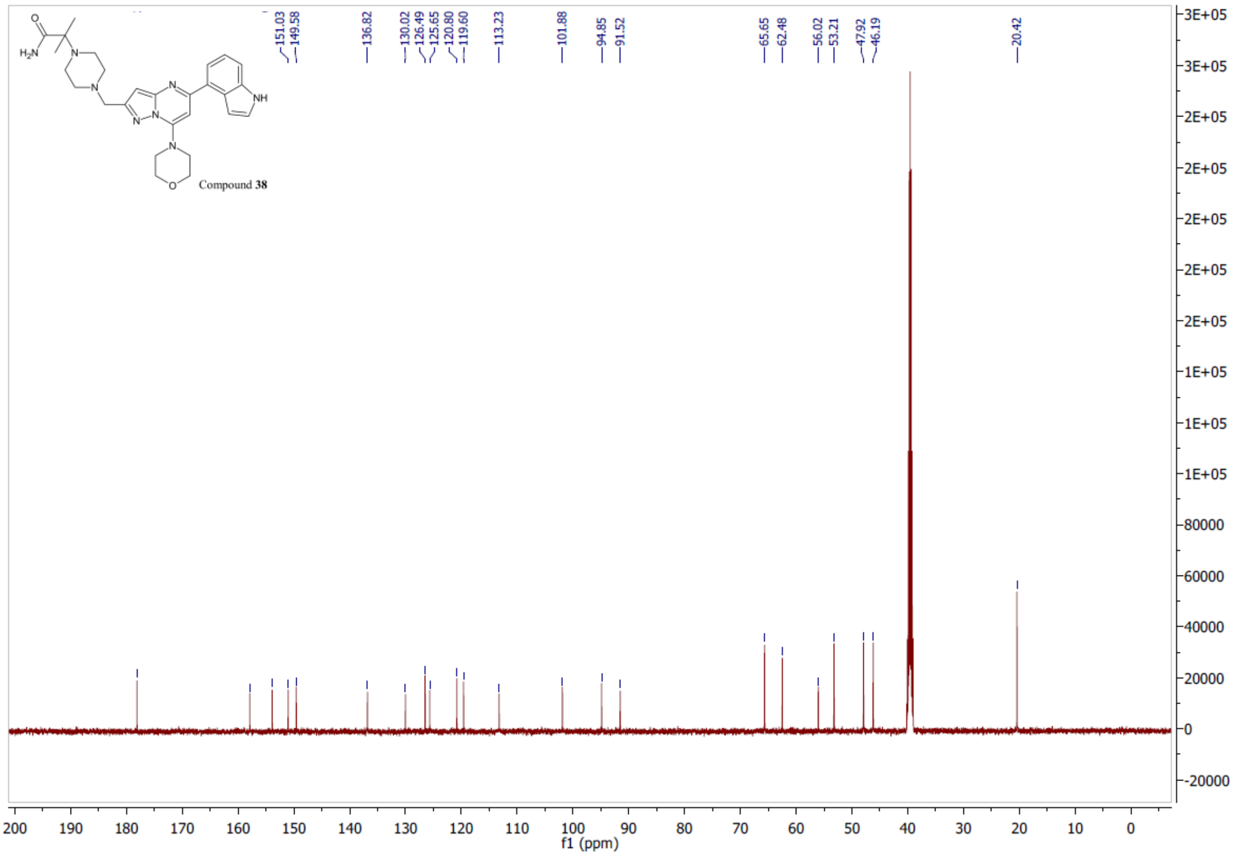
Compound 38
151.03 149.58 136.82 130.02 126.49 125.65 120.80 119.60 113.23 101.88 94.85 91.52
65.65 62.48 56.02 53.21 47.92 46.19 20.42
f1 (ppm)

Compound 38

Name	*Obs. m/z*	Obs. RT	Obs. Mass	Tgt Formula	Tgt Mass	Tgt Mass Error	Find Cpds Algorith
Compound **38**	525.2722	1.11	502.2828	C27 H34 N8 O2	502.2805	4.69	Find by Formula

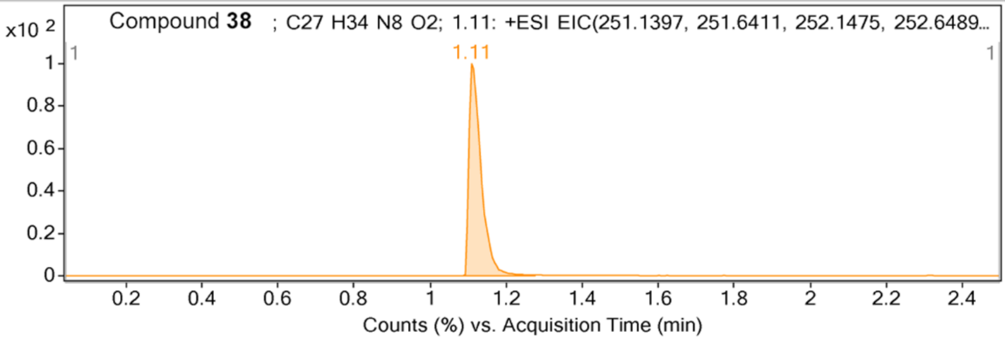

MS Zoomed Spectrum

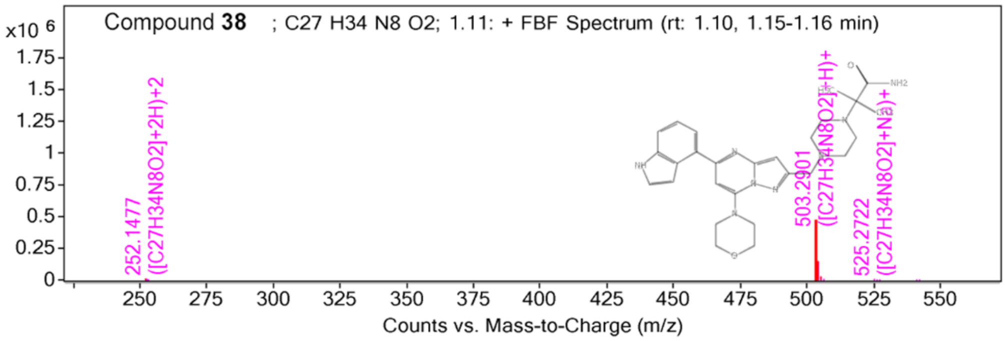

MS Zoomed Spectrum

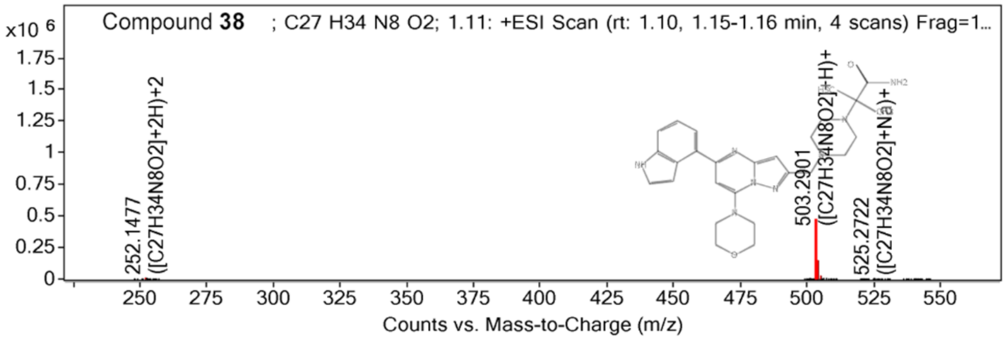

MS Spectrum Peak List

Obs. m/z	Charge	Abund	Formula	Ion/Isotope	Tgt Mass Error (ppm)
503.2901	1	469024.59	C27H34N8O2	(M+H)+	
503.2901	1	469024.59	C27H34N8O2	(M+H)+	4.59

--- End Of Report ---

Compound 39

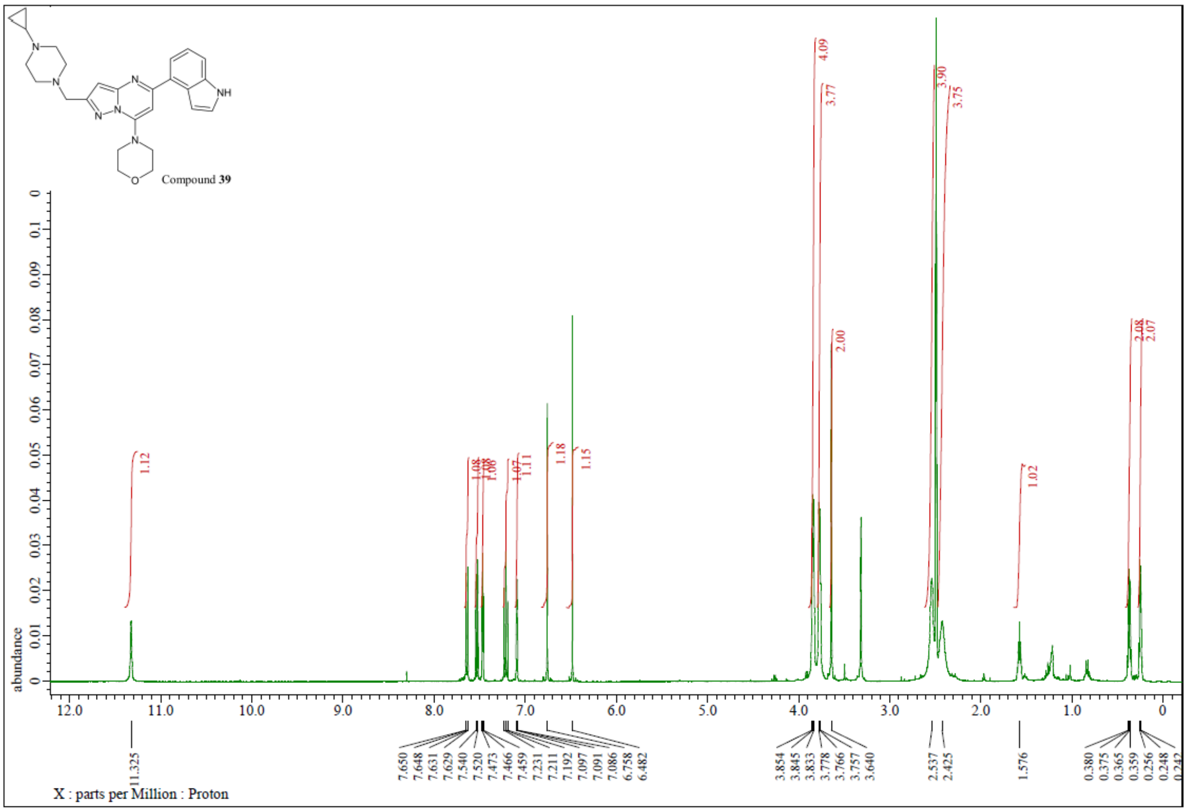

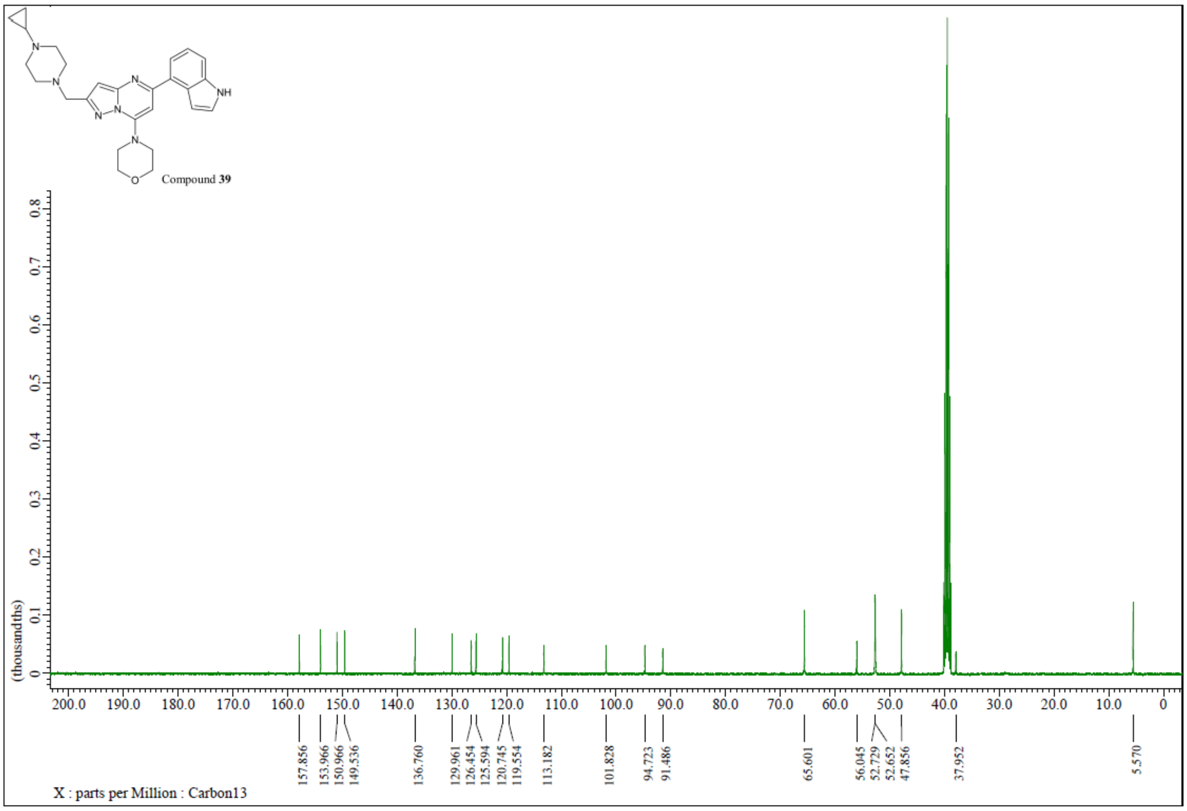

Compound 39

Name	*Obs. m/z*	Obs. RT	Obs. Mass	Tgt Formula	Tgt Mass	Tgt Mass Error	Find Cpds Algorith
Compound **39**	458.26656	1.12	457.2599	C26 H31 N7 O	457.25901	1.94	Find by Formula

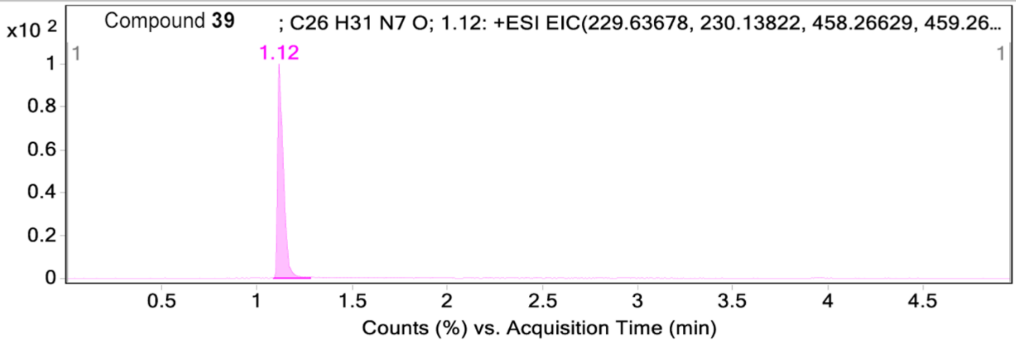

MS Zoomed Spectrum

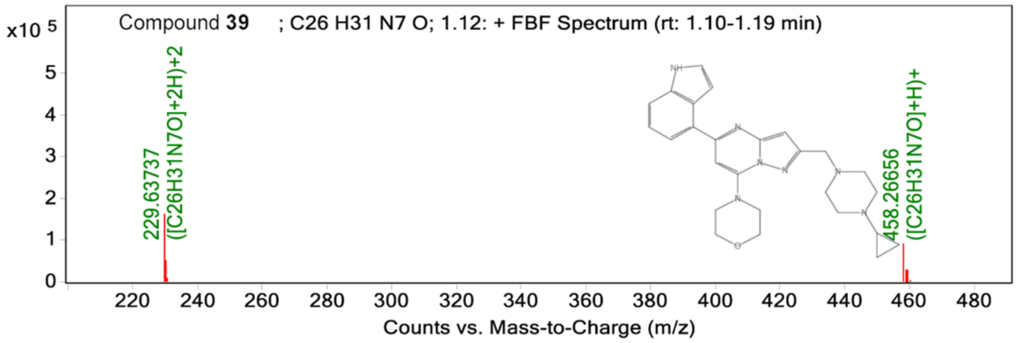

MS Zoomed Spectrum

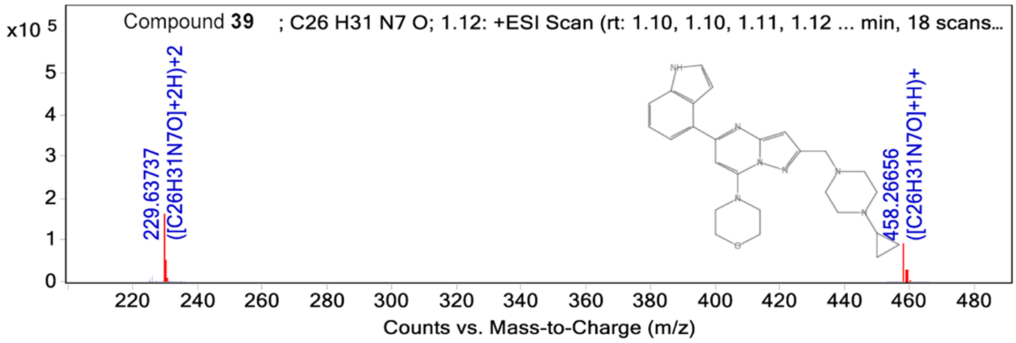

MS Spectrum Peak List

Obs. m/z	Charge	Abund	Formula	Ion/Isotope	Tgt Mass Error (ppm)
229.63737	2	163876.17	C26H31N7O	(M+2H)+2	
230.139	2	49603.69	C26H31N7O	(M+2H)+2	
230.64031	2	7768.41	C26H31N7O	(M+2H)+2	
231.13724	2	993.14	C26H31N7O	(M+2H)+2	
458.26656	1	90851.98	C26H31N7O	(M+H)+	
459.26935	1	28360.6	C26H31N7O	(M+H)+	
460.27201	1	4396.89	C26H31N7O	(M+H)+	
461.27623	1	511.48	C26H31N7O	(M+H)+	
229.63737	2	163876.17	C26H31N7O	(M+2H)+2	2.58
230.139	2	49603.69	C26H31N7O	(M+2H)+2	3.41
230.64031	2	7768.41	C26H31N7O	(M+2H)+2	3.05
231.13724	2	993.14	C26H31N7O	(M+2H)+2	-16.14
458.26656	1	90851.98	C26H31N7O	(M+H)+	0.6
459.26935	1	28360.6	C26H31N7O	(M+H)+	0.41
460.27201	1	4396.89	C26H31N7O	(M+H)+	0.14
461.27623	1	511.48	C26H31N7O	(M+H)+	3.4

--- End Of Report ---

Compound 40

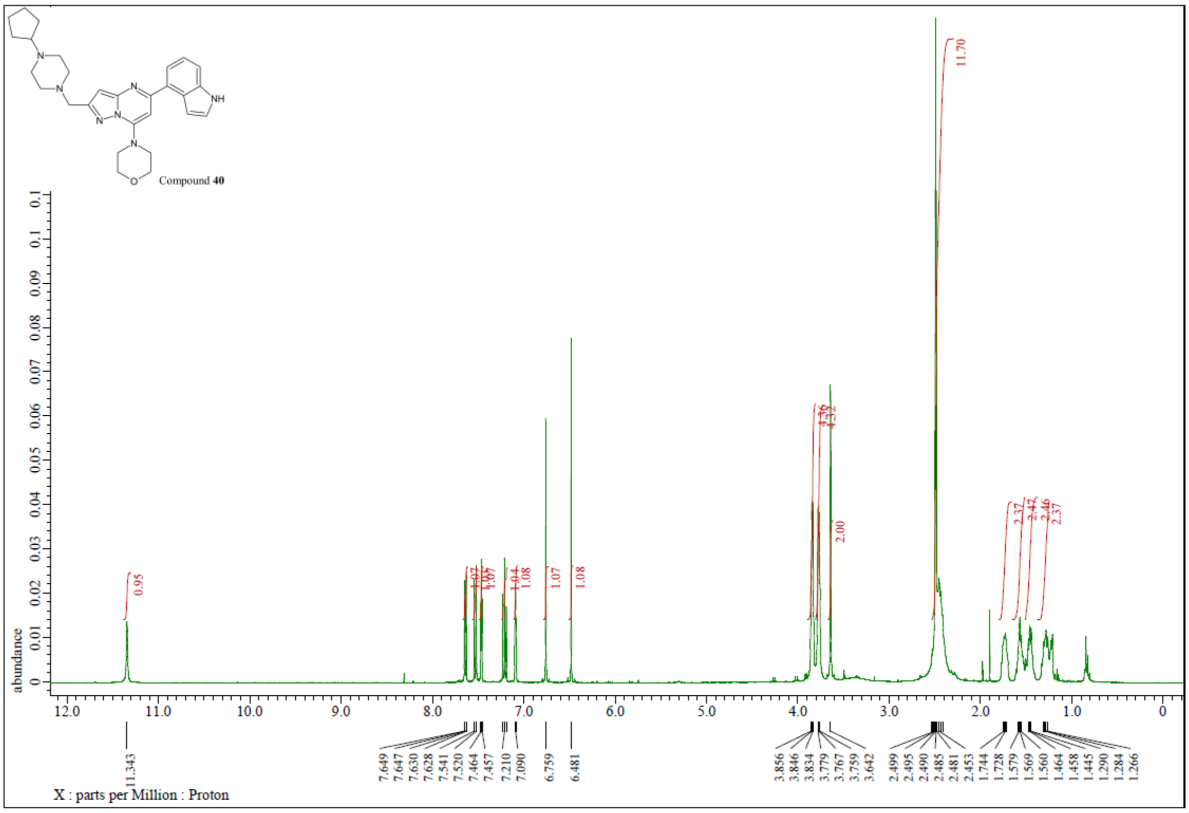

Compound 40
abundance
X : parts per Million : Proton
11.343
7.649
7.647
7.630
7.628
7.541
7.520
7.464
7.457
7.210
7.090
6.759
6.481
3.856
3.846
3.834
3.779
3.767
3.759
3.642
2.499
2.495
2.490
2.485
2.481
2.453
1.744
1.728
1.579
1.569
1.560
1.464
1.458
1.445
1.290
1.284
1.266

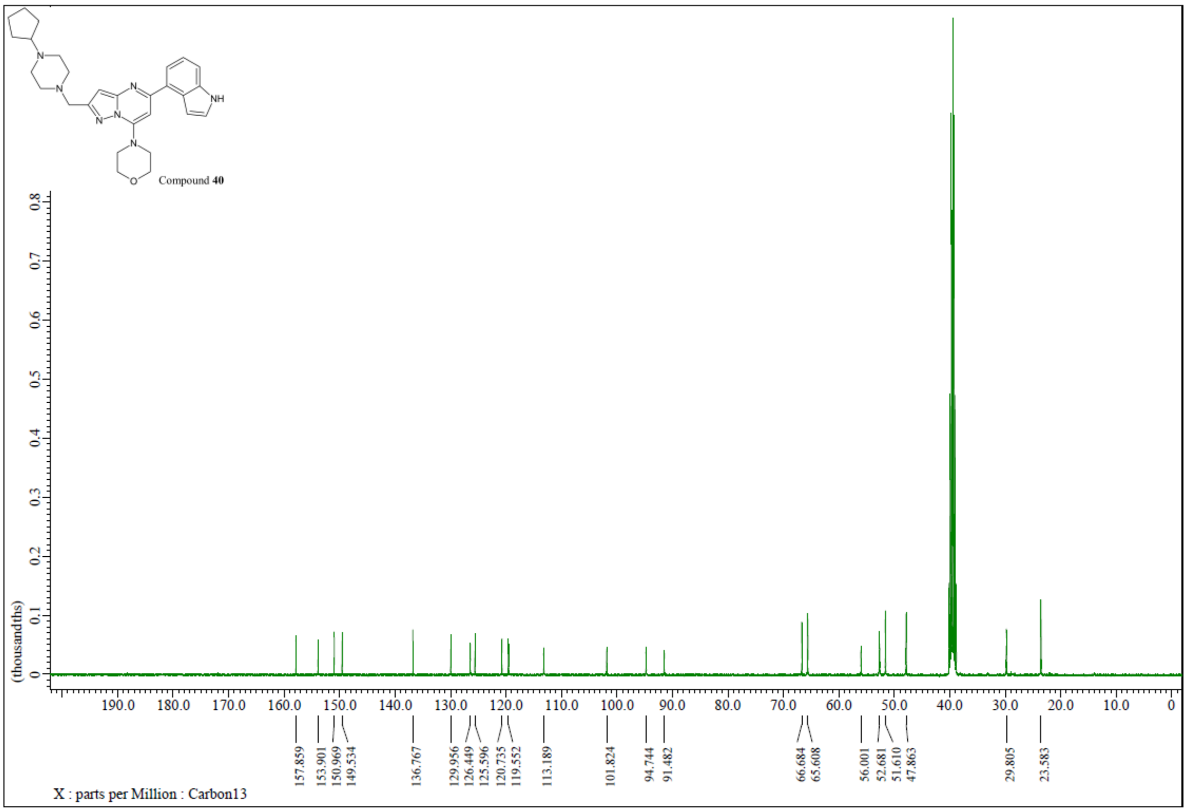

Compound 40
(thousandths)
X : parts per Million : Carbon13
157.859
153.901
150.969
149.534
136.767
129.956
126.449
125.596
120.735
119.552
113.189
101.824
94.744
91.482
66.684
65.608
56.001
52.681
51.610
47.863
29.805
23.583

Compound 40

Name	*Obs. m/z*	Obs. RT	Obs. Mass	Tgt Formula	Tgt Mass	Tgt Mass Error	Find Cpds Algorith
Compound **40**	486.29731	1.14	485.29115	C28 H35 N7 O	485.29031	1.73	Find by Formula

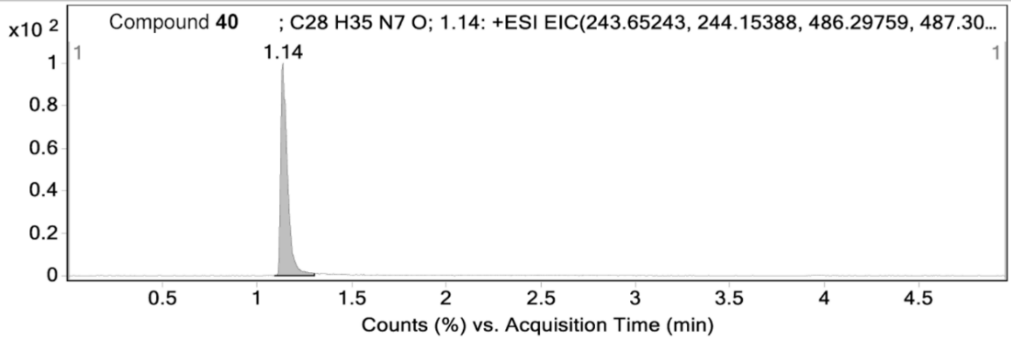

MS Zoomed Spectrum

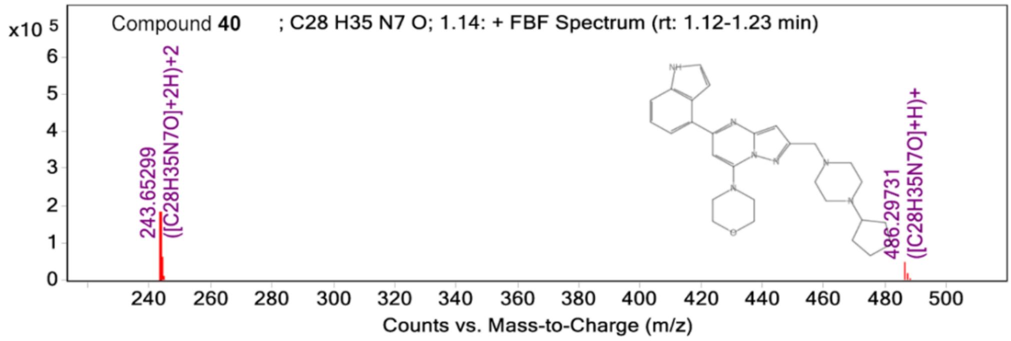

MS Zoomed Spectrum

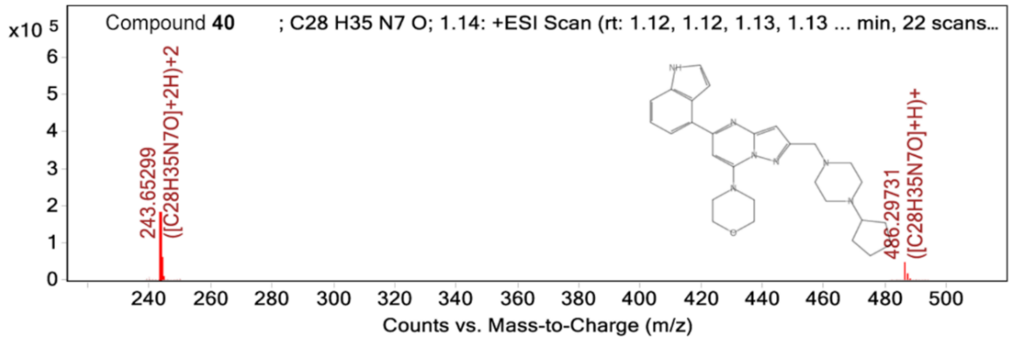

MS Spectrum Peak List

Obs. m/z	Charge	Abund	Formula	Ion/Isotope	Tgt Mass Error (ppm)
243.65299	2	183132.14	C28H35N7O	(M+2H)+2	
244.1546	2	57179.7	C28H35N7O	(M+2H)+2	
244.65581	2	9451.18	C28H35N7O	(M+2H)+2	
245.1542	2	1692.28	C28H35N7O	(M+2H)+2	
486.29731	1	47318.36	C28H35N7O	(M+H)+	
487.30016	1	15779.74	C28H35N7O	(M+H)+	
488.30307	1	2791.22	C28H35N7O	(M+H)+	
489.30436	1	349.8	C28H35N7O	(M+H)+	
243.65299	2	183132.14	C28H35N7O	(M+2H)+2	2.28
244.1546	2	57179.7	C28H35N7O	(M+2H)+2	2.95
244.65581	2	9451.18	C28H35N7O	(M+2H)+2	2.07
245.1542	2	1692.28	C28H35N7O	(M+2H)+2	-10.14
486.29731	1	47318.36	C28H35N7O	(M+H)+	-0.57
487.30016	1	15779.74	C28H35N7O	(M+H)+	-0.68
488.30307	1	2791.22	C28H35N7O	(M+H)+	-0.53
489.30436	1	349.8	C28H35N7O	(M+H)+	-3.52

--- End Of Report ---

Compound 41

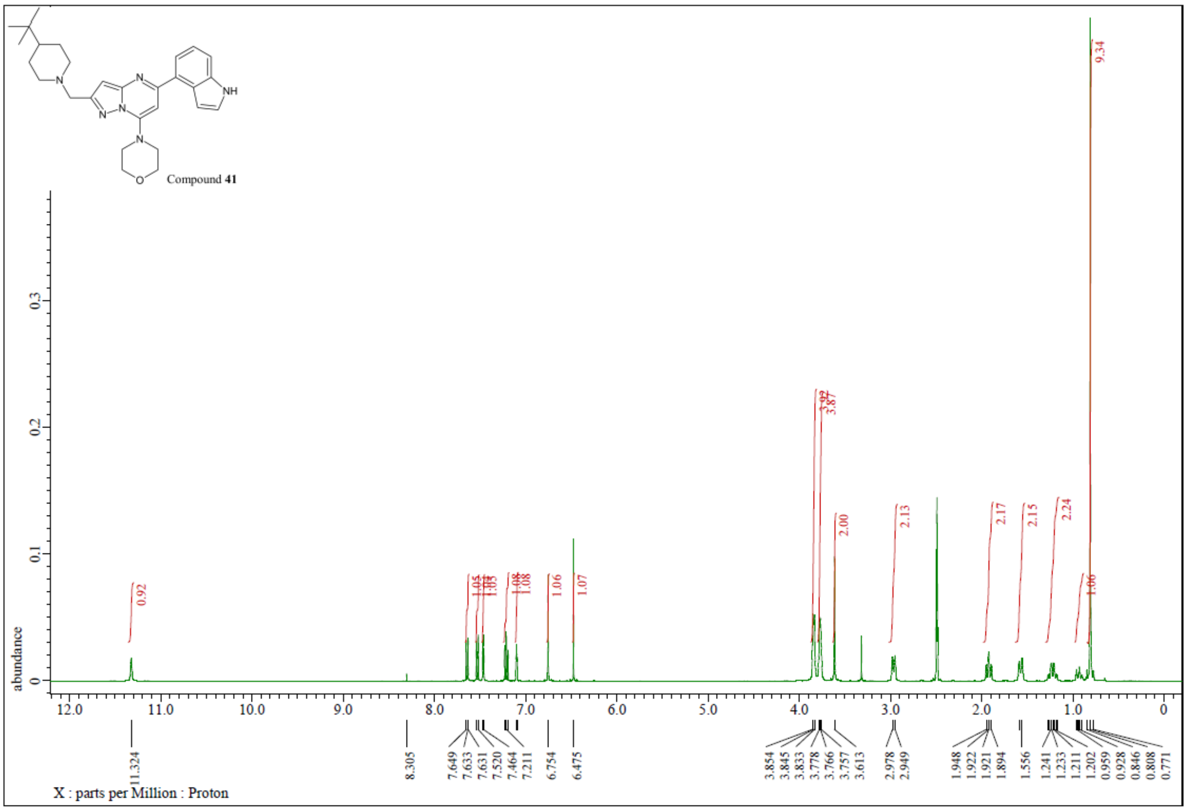

Compound 41
abundance
X : parts per Million : Proton
11.324
8.305
7.649
7.633
7.631
7.520
7.464
7.211
6.754
6.475
3.854
3.845
3.833
3.778
3.766
3.757
3.613
2.978
2.949
1.948
1.922
1.921
1.894
1.556
1.241
1.233
1.211
1.202
0.959
0.928
0.846
0.808
0.771
9.34

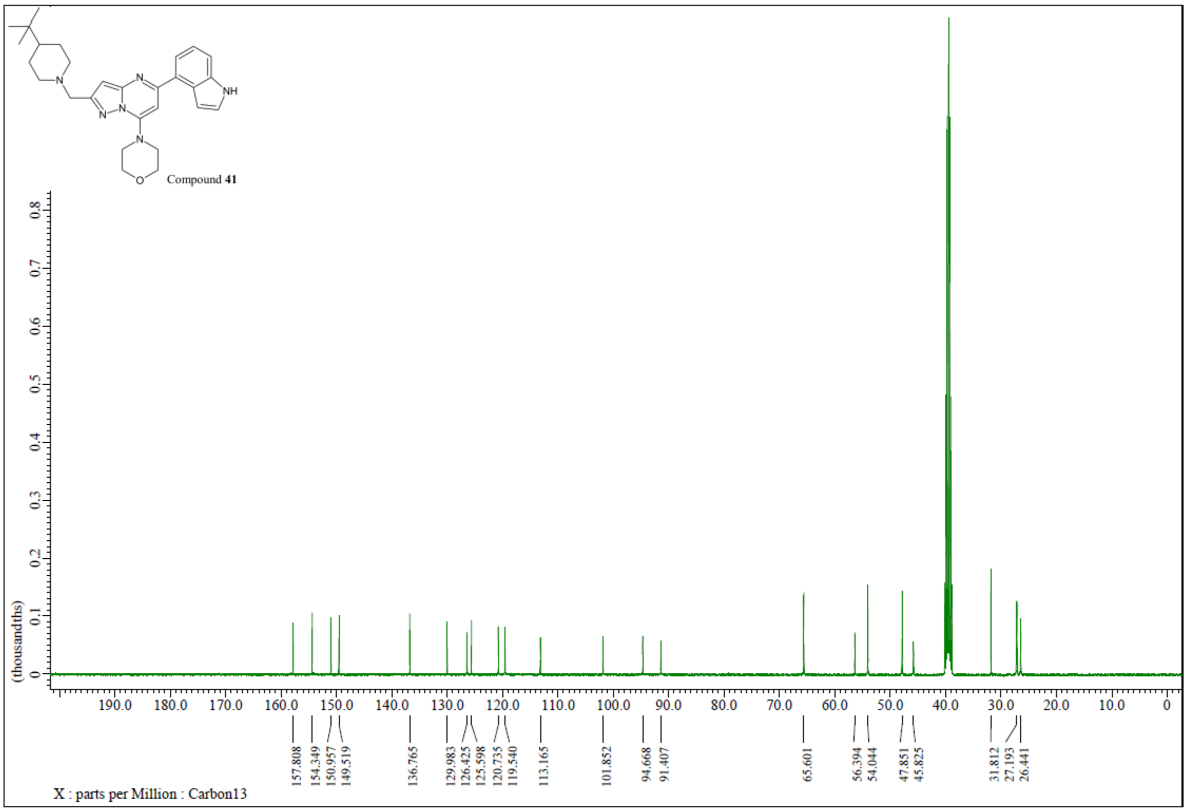

Compound 41
(thousandths)
X : parts per Million : Carbon13
157.808
154.349
150.957
149.519
136.765
129.983
126.425
125.598
120.735
119.540
113.165
101.852
94.668
91.407
65.601
56.394
54.044
47.851
45.825
31.812
27.193
26.441

Compound 41

Name	Obs. m/z	Obs. RT	Obs. Mass	Tgt Formula	Tgt Mass	Tgt Mass Error	Find Cpds Algorith
Compound **41**	237.15538	1.58	472.29562	C28 H36 N6 O	472.29506	1.18	Find by Formula

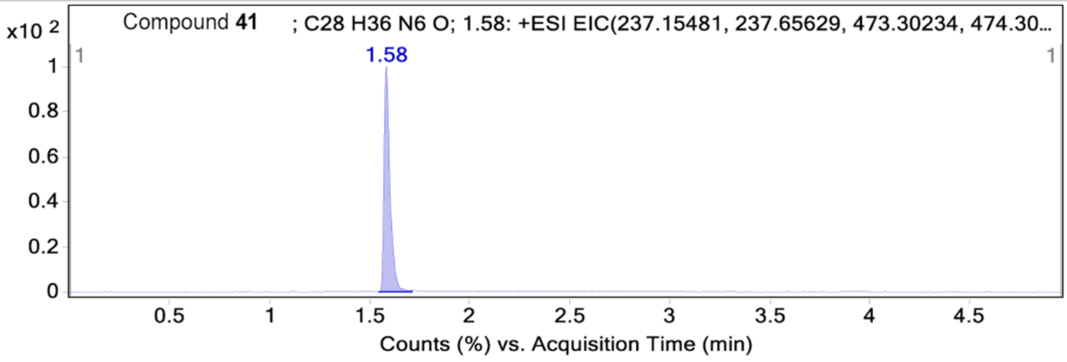

MS Zoomed Spectrum

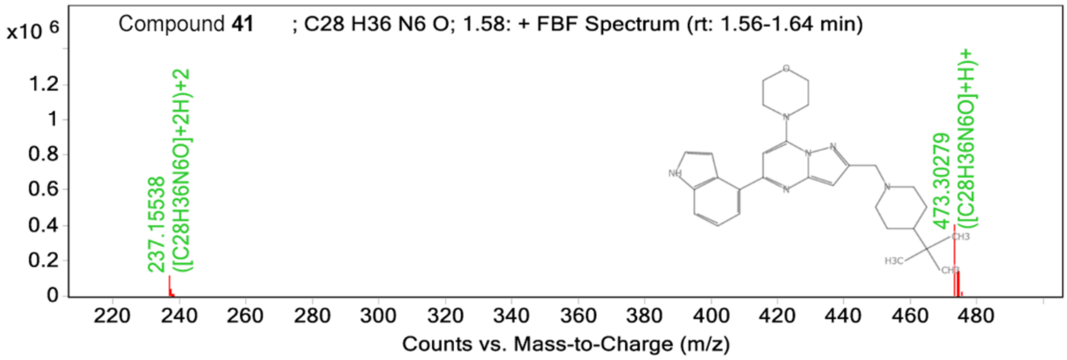

MS Zoomed Spectrum

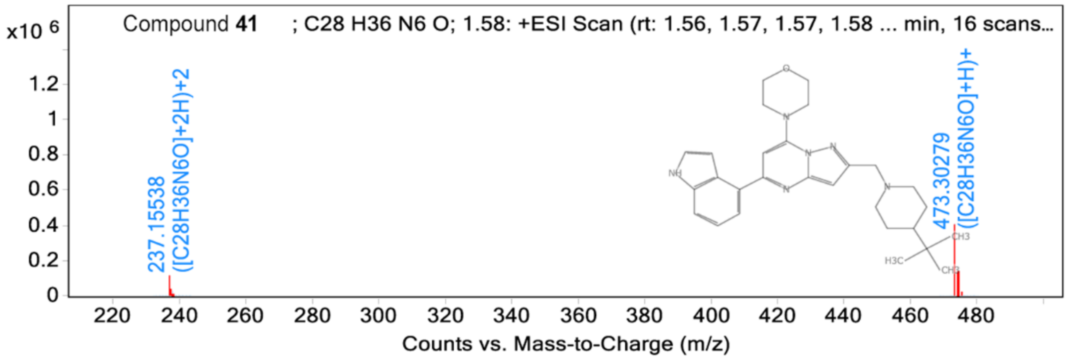

MS Spectrum Peak List

Obs. m/z	Charge	Abund	Formula	Ion/Isotope	Tgt Mass Error (ppm)
237.15538	2	111387.02	C28H36N6O	(M+2H)+2	
237.65676	2	36742.8	C28H36N6O	(M+2H)+2	
238.15755	2	6752.31	C28H36N6O	(M+2H)+2	
238.65771	2	829.94	C28H36N6O	(M+2H)+2	
473.30279	1	407145.97	C28H36N6O	(M+H)+	
474.30575	1	121042.59	C28H36N6O	(M+H)+	
475.30801	1	19658.06	C28H36N6O	(M+H)+	
237.15538	2	111387.02	C28H36N6O	(M+2H)+2	2.42
237.65676	2	36742.8	C28H36N6O	(M+2H)+2	1.97
238.15755	2	6752.31	C28H36N6O	(M+2H)+2	-0.8
238.65771	2	829.94	C28H36N6O	(M+2H)+2	-6.03
473.30279	1	407145.97	C28H36N6O	(M+H)+	0.96
474.30575	1	121042.59	C28H36N6O	(M+H)+	0.94
475.30801	1	19658.06	C28H36N6O	(M+H)+	-0.41

--- End Of Report ---

Compound 42

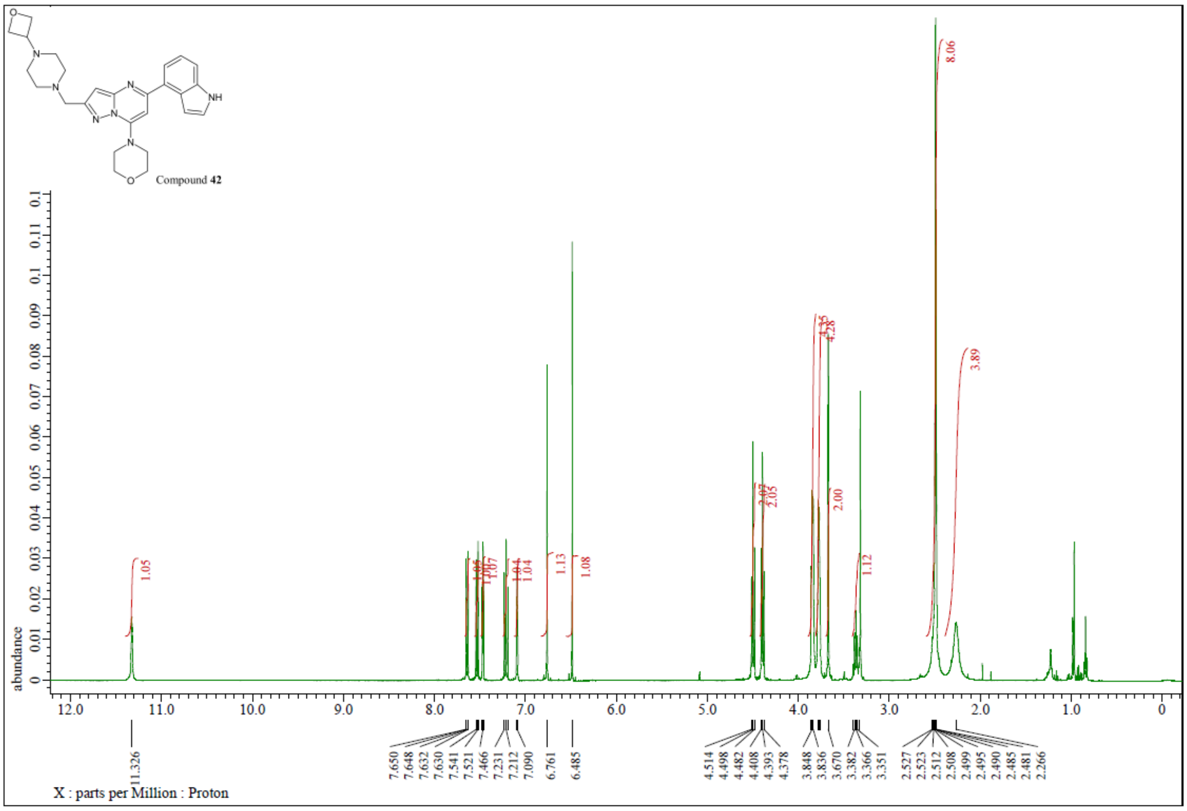

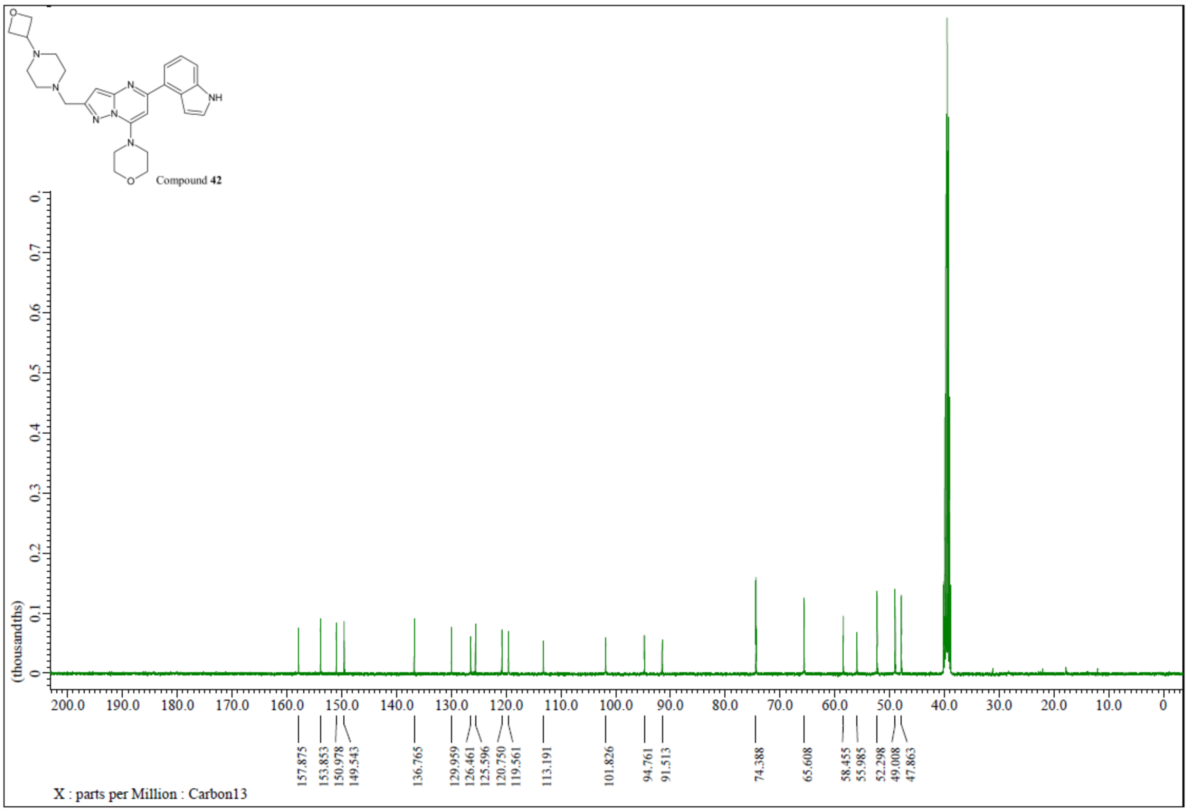

Compound 42

Name	*Obs. m/z*	Obs. RT	Obs. Mass	Tgt Formula	Tgt Mass	Tgt Mass Error	Find Cpds Algorith
Compound **42**	474.2616	1.06	473.25456	C26 H31 N7 O2	473.25392	1.35	Find by Formula

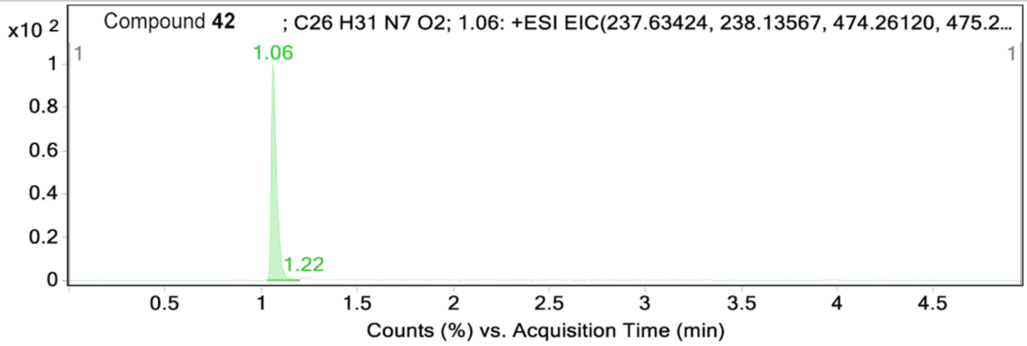

MS Zoomed Spectrum

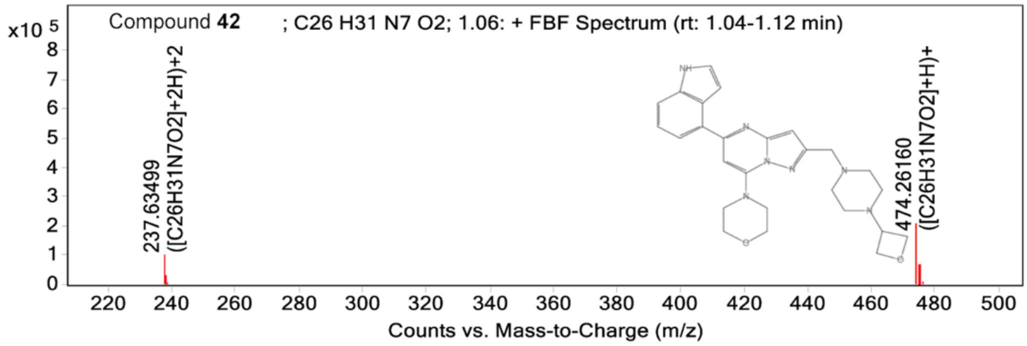

MS Zoomed Spectrum

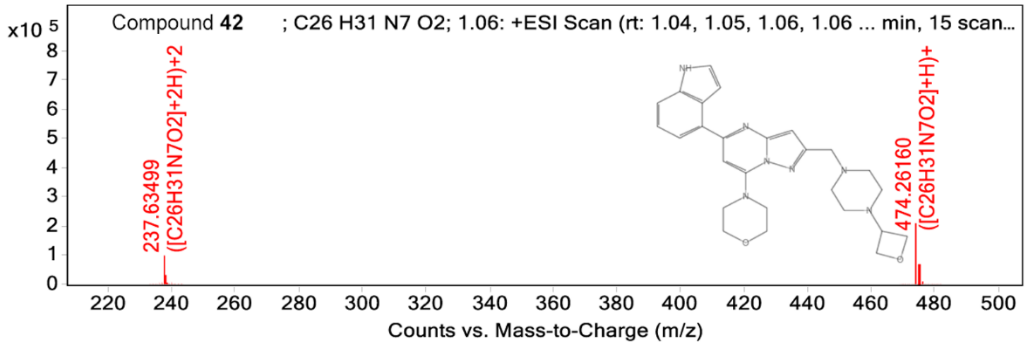

MS Spectrum Peak List

Obs. m/z	Charge	Abund	Formula	Ion/Isotope	Tgt Mass Error (ppm)
237.63499	2	99213.34	C26H31N7O2	(M+2H)+2	
238.13576	2	31857.8	C26H31N7O2	(M+2H)+2	
238.63738	2	4981.81	C26H31N7O2	(M+2H)+2	
474.2616	1	207817.77	C26H31N7O2	(M+H)+	
475.26443	1	63939.76	C26H31N7O2	(M+H)+	
476.267	1	9934.29	C26H31N7O2	(M+H)+	
477.27038	1	1159.53	C26H31N7O2	(M+H)+	
237.63499	2	99213.34	C26H31N7O2	(M+2H)+2	3.16
238.13576	2	31857.8	C26H31N7O2	(M+2H)+2	0.35
238.63738	2	4981.81	C26H31N7O2	(M+2H)+2	1.42
474.2616	1	207817.77	C26H31N7O2	(M+H)+	0.84
475.26443	1	63939.76	C26H31N7O2	(M+H)+	0.75
476.267	1	9934.29	C26H31N7O2	(M+H)+	0.42
477.27038	1	1159.53	C26H31N7O2	(M+H)+	1.96

--- End Of Report ---

Compound 43

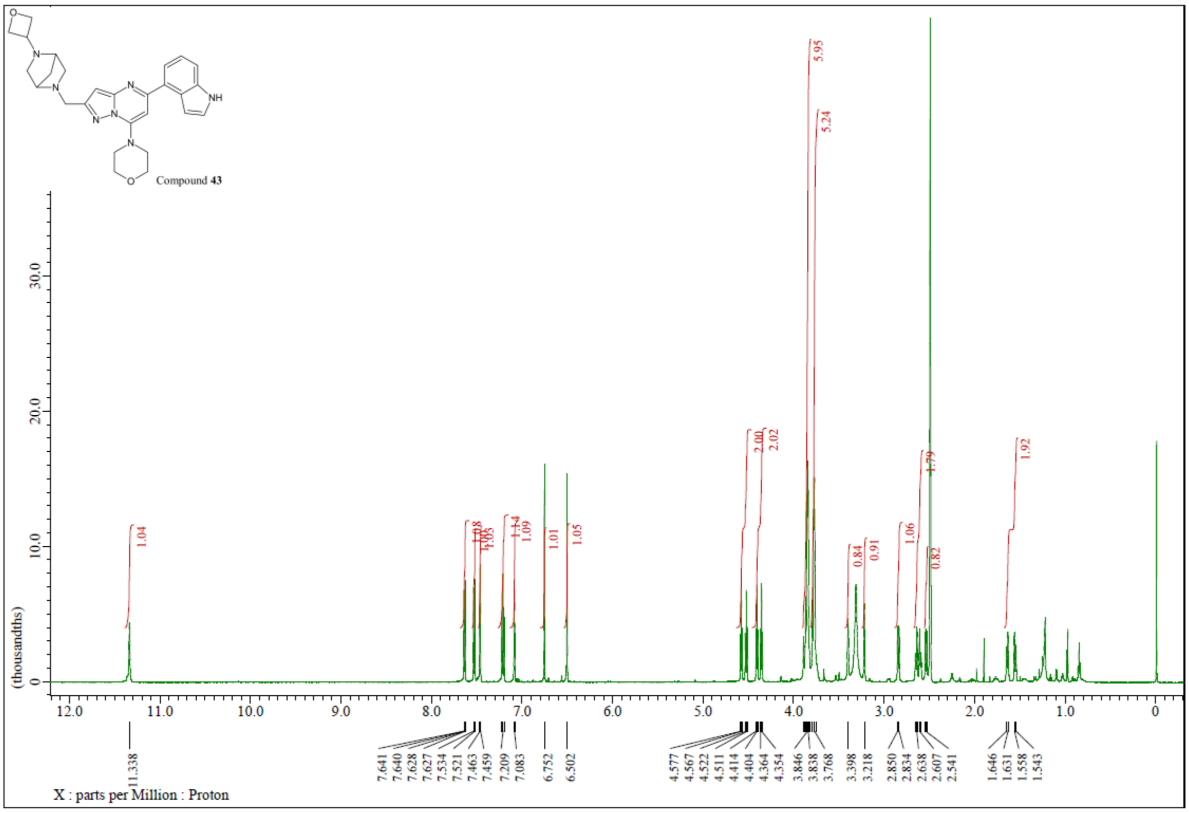
Compound 43
X : parts per Million : Proton
(thousandths)
11.338
7.641 7.640 7.628 7.627 7.534 7.521 7.463 7.459 7.209 7.083 6.752 6.502
4.577 4.567 4.522 4.511 4.414 4.404 4.364 4.354 3.846 3.838 3.768 3.398 3.218 2.850 2.834 2.638 2.607 2.541
1.646 1.631 1.558 1.543

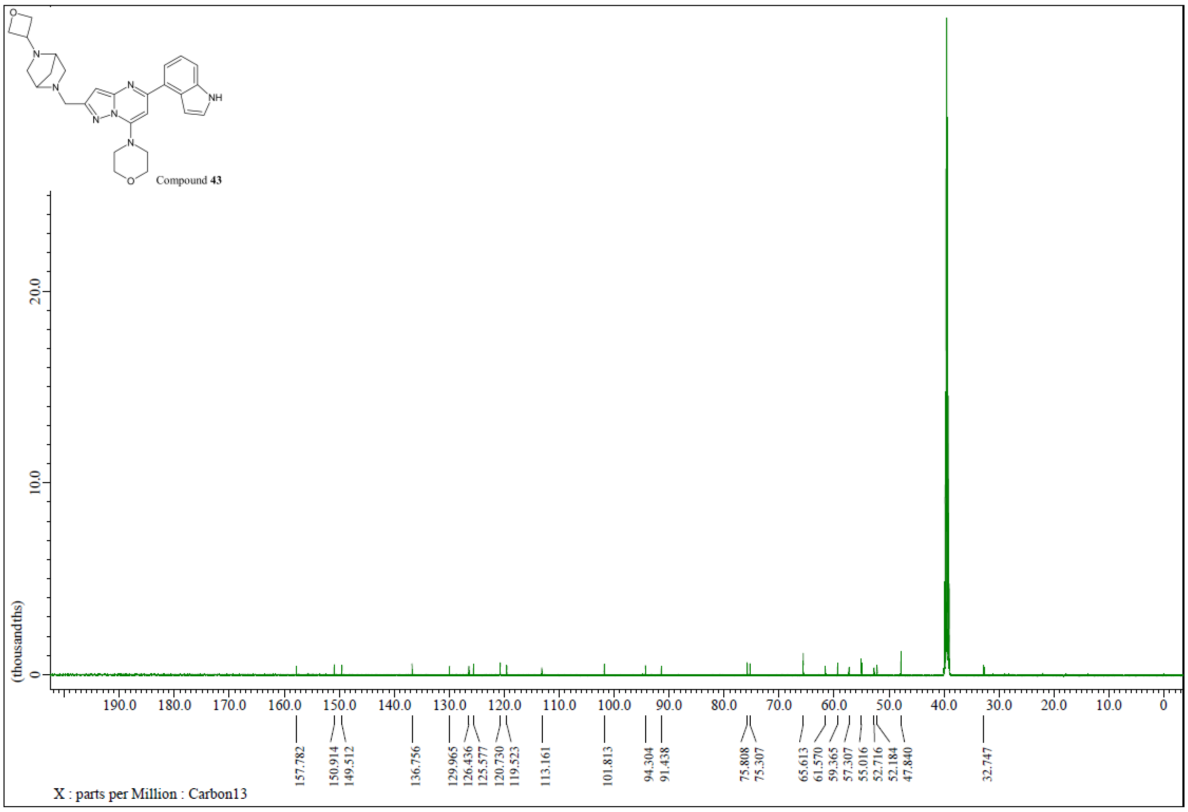
Compound 43
X : parts per Million : Carbon13
(thousandths)
157.782 150.914 149.512 136.756 129.965 126.436 125.577 120.730 119.523 113.161 101.813 94.304 91.438 75.808 75.307 65.613 61.570 59.365 57.307 55.016 52.716 52.184 47.840 32.747

Compound 43

Name	*Obs. m/z*	Obs. RT	Obs. Mass	Tgt Formula	Tgt Mass	Tgt Mass Error	Find Cpds Algorith
Compound **43**	486.26135	1.05	485.25477	C27 H31 N7 O2	485.25392	1.74	Find by Formula

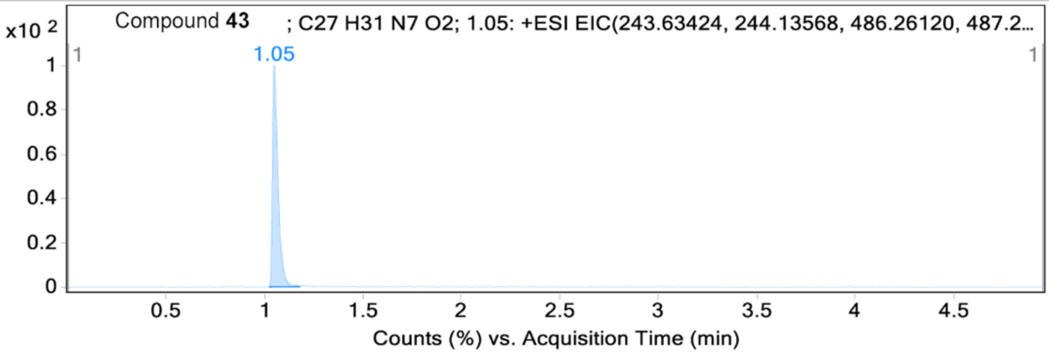

MS Zoomed Spectrum

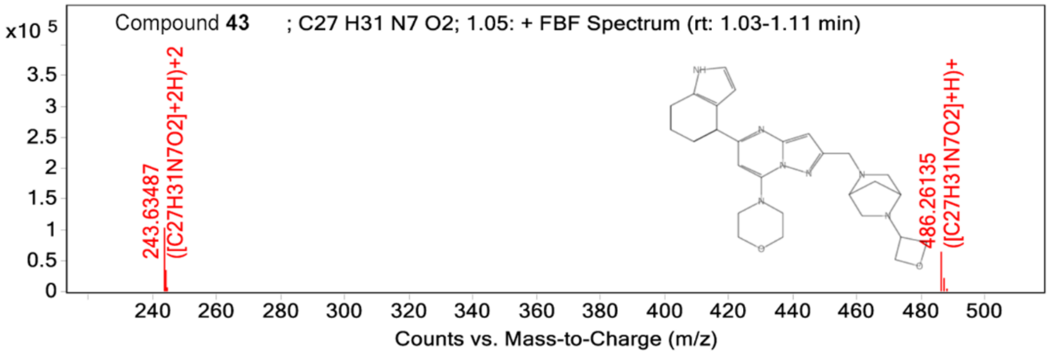

MS Zoomed Spectrum

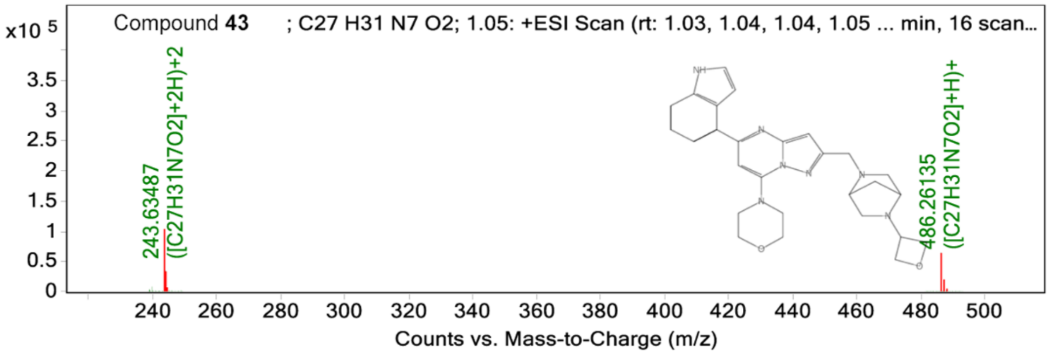

MS Spectrum Peak List

Obs. m/z	Charge	Abund	Formula	Ion/Isotope	Tgt Mass Error (ppm)
243.63487	2	102960.16	C27H31N7O2	(M+2H)+2	
244.13647	2	34634.86	C27H31N7O2	(M+2H)+2	
244.63749	2	5934.04	C27H31N7O2	(M+2H)+2	
486.26135	1	64416.76	C27H31N7O2	(M+H)+	
487.26383	1	19490.92	C27H31N7O2	(M+H)+	
488.2669	1	3146.05	C27H31N7O2	(M+H)+	
243.63487	2	102960.16	C27H31N7O2	(M+2H)+2	2.6
244.13647	2	34634.86	C27H31N7O2	(M+2H)+2	3.23
244.63749	2	5934.04	C27H31N7O2	(M+2H)+2	1.75
486.26135	1	64416.76	C27H31N7O2	(M+H)+	0.31
487.26383	1	19490.92	C27H31N7O2	(M+H)+	-0.53
488.2669	1	3146.05	C27H31N7O2	(M+H)+	0.12

--- End Of Report ---

Compound 44

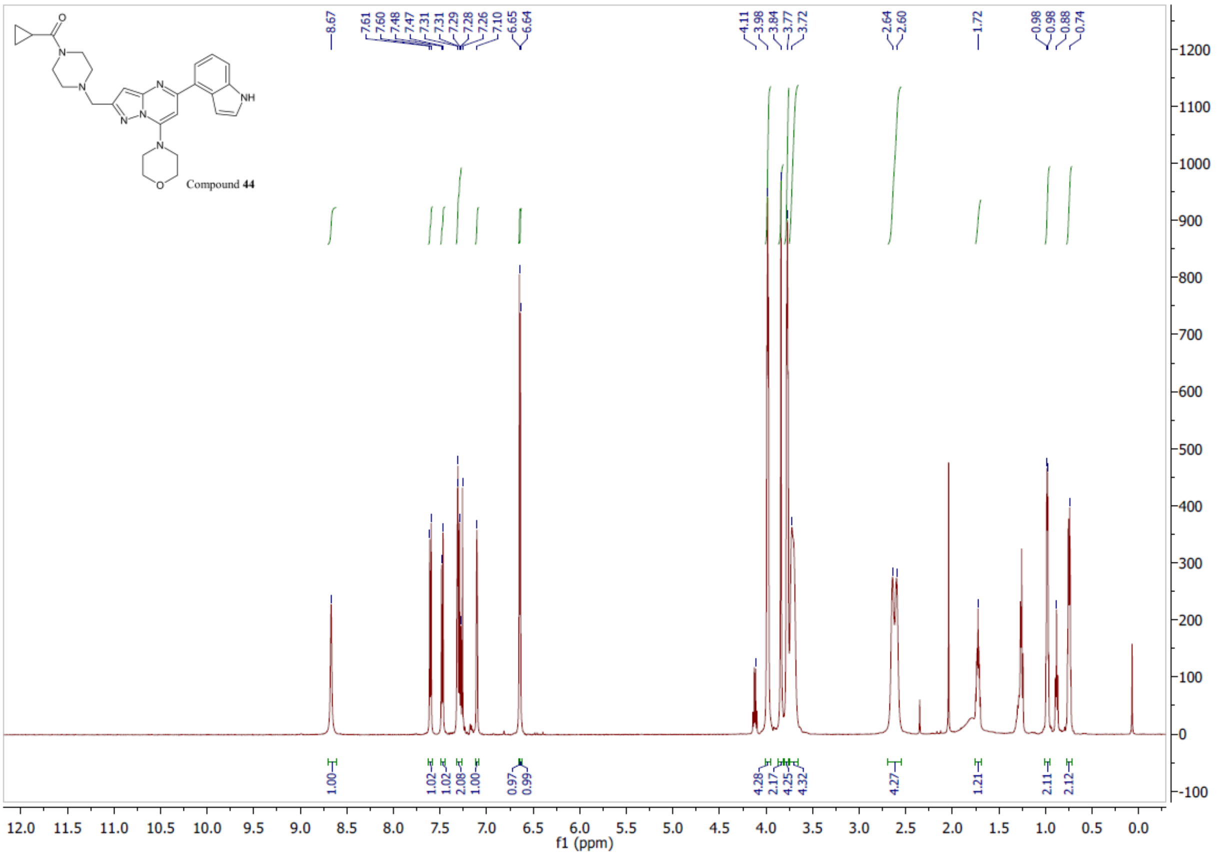

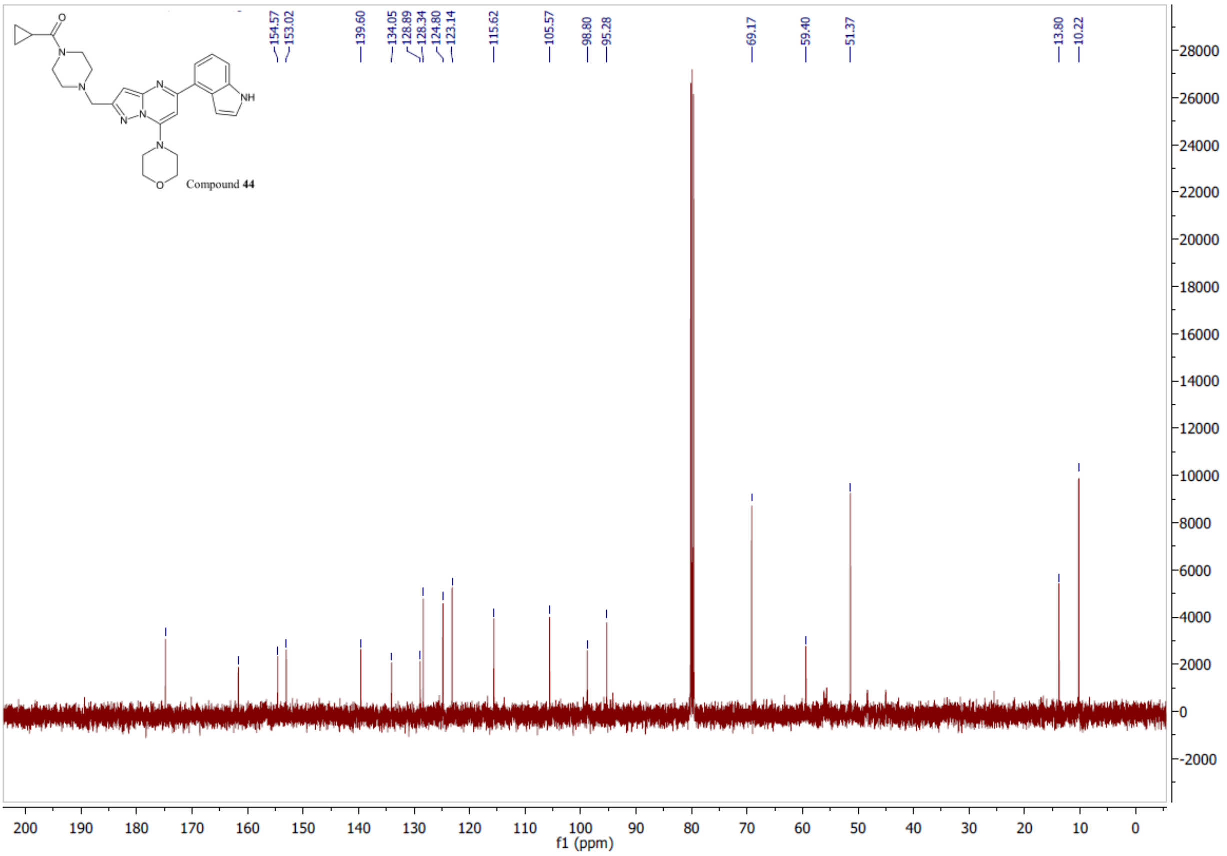

Compound 44

Compound Label	Name	*m/z*	RT	Algorithm	Mass
Compound **44**	Compound **44**	243.6344	1.3	Find by Formula	485.25457

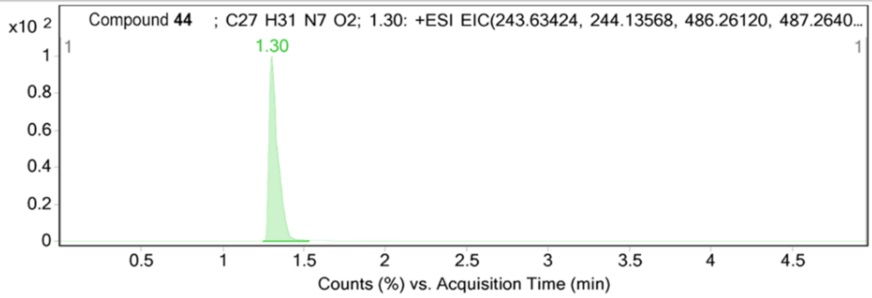

MS Zoomed Spectrum

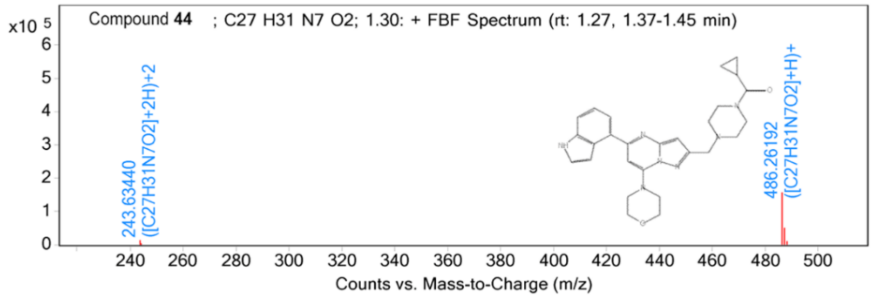

MS Spectrum Peak List

m/z	z	Abund	Formula	Ion
243.6344	2	12024.05	C27H31N7O2	(M+2H)+2
244.13608	2	3727.14	C27H31N7O2	(M+2H)+2
244.63743	2	671.81	C27H31N7O2	(M+2H)+2
486.26192	1	156276.28	C27H31N7O2	(M+H)+
487.26456	1	46874.78	C27H31N7O2	(M+H)+
488.26754	1	7930.26	C27H31N7O2	(M+H)+

MS Spectrum

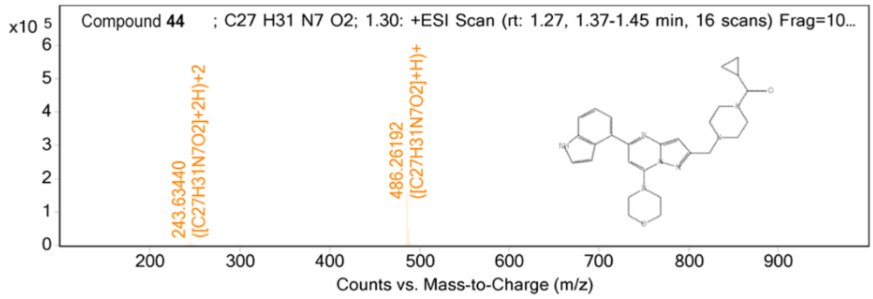

MS Zoomed Spectrum

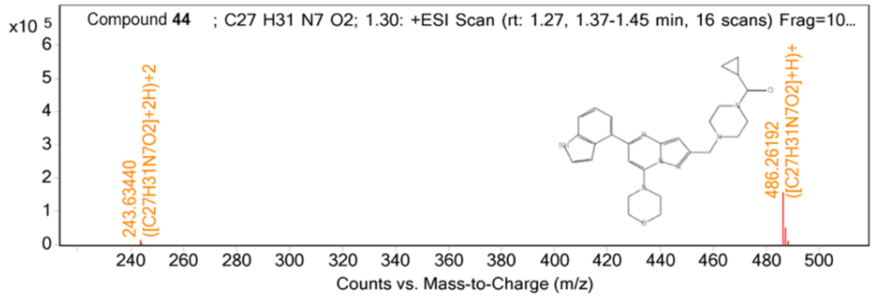

MS Spectrum Peak List

m/z	*Calc m/z*	Diff(ppm)	z	Abund	Formula	Ion
243.6344	243.63424	0.68	2	12024.05	C27H31N7O2	(M+2H)+2
244.13608	244.13568	1.62	2	3727.14	C27H31N7O2	(M+2H)+2
244.63743	244.63706	1.52	2	671.81	C27H31N7O2	(M+2H)+2
486.26192	486.2612	1.49	1	156276.28	C27H31N7O2	(M+H)+
487.26456	487.26409	0.97	1	46874.78	C27H31N7O2	(M+H)+
488.26754	488.26684	1.43	1	7930.26	C27H31N7O2	(M+H)+

--- End Of Report ---

Compound 49

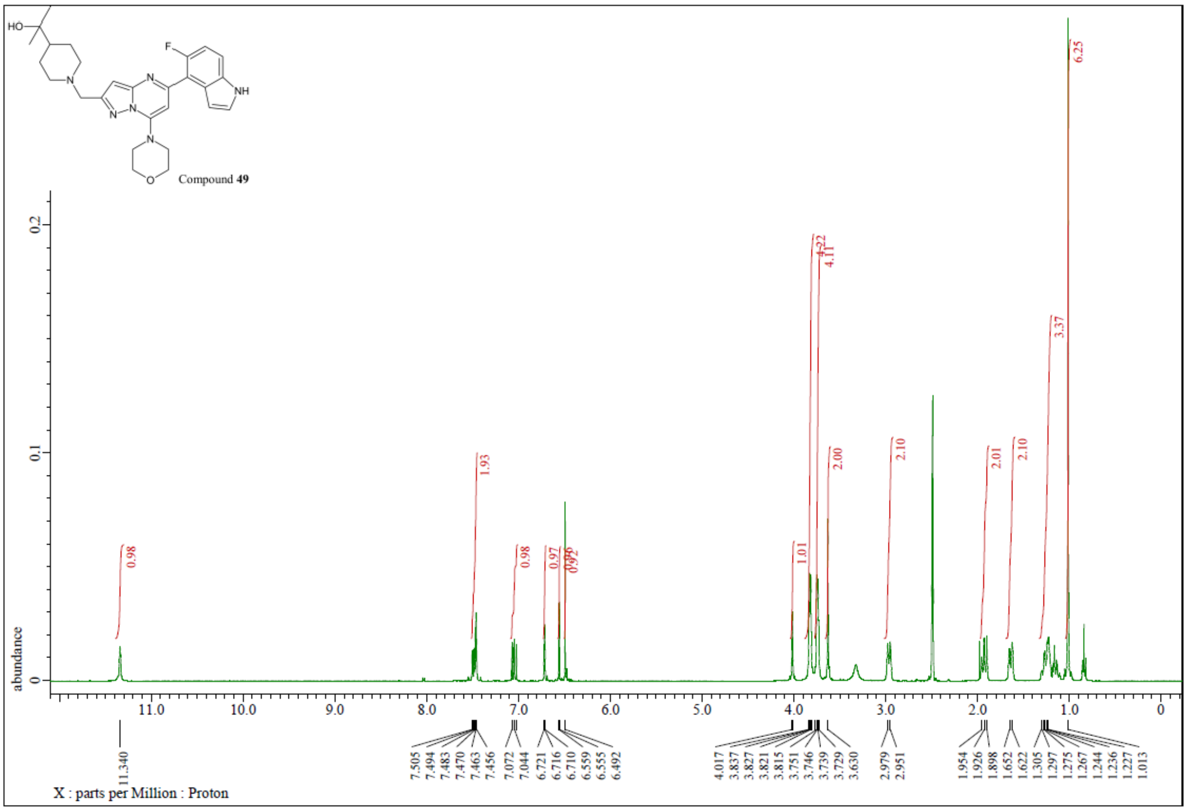
Compound 49
abundance
X : parts per Million : Proton

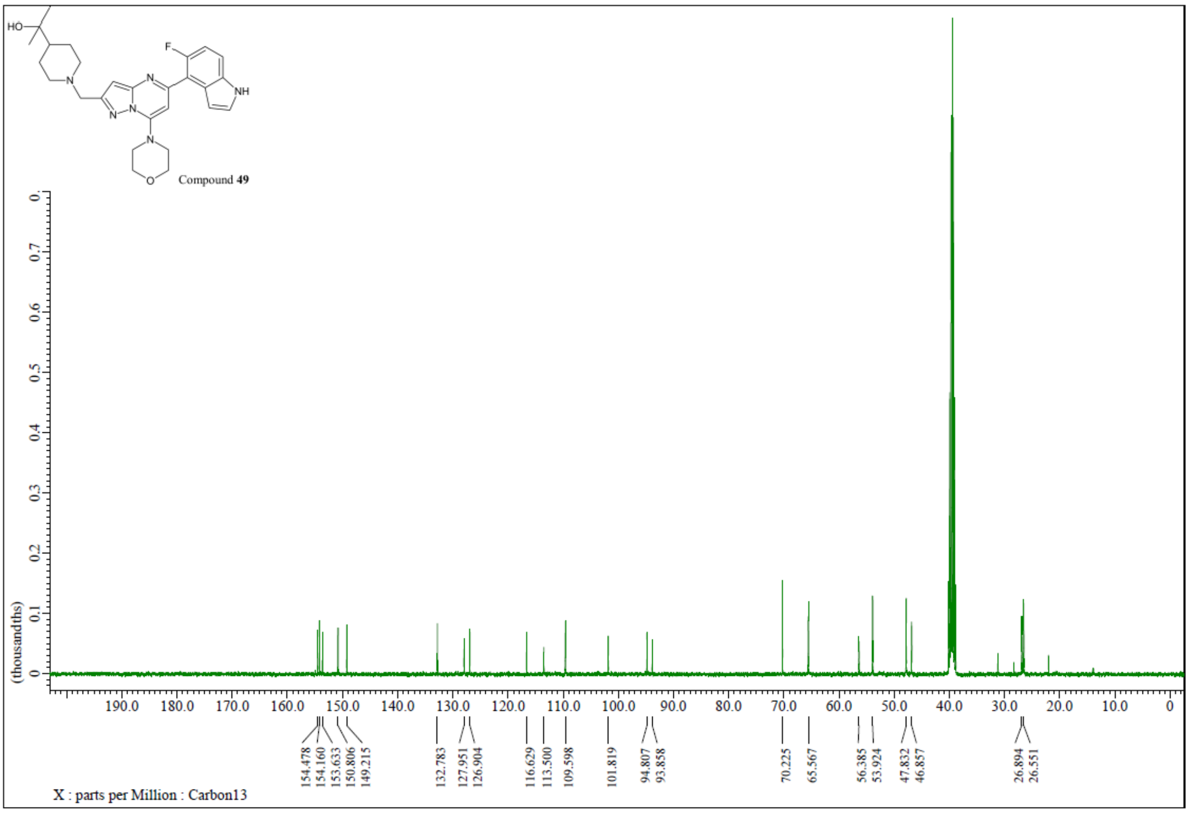
Compound 49
(thousandths)
X : parts per Million : Carbon13

Compound 49

Name	*Obs. m/z*	Obs. RT	Obs. Mass	Tgt Formula	Tgt Mass	Tgt Mass Error	Find Cpds Algorit
Compound **49**	493.27235	1.23	492.26532	C27 H33 F N6 O2	492.2649	0.86	Find by Formula

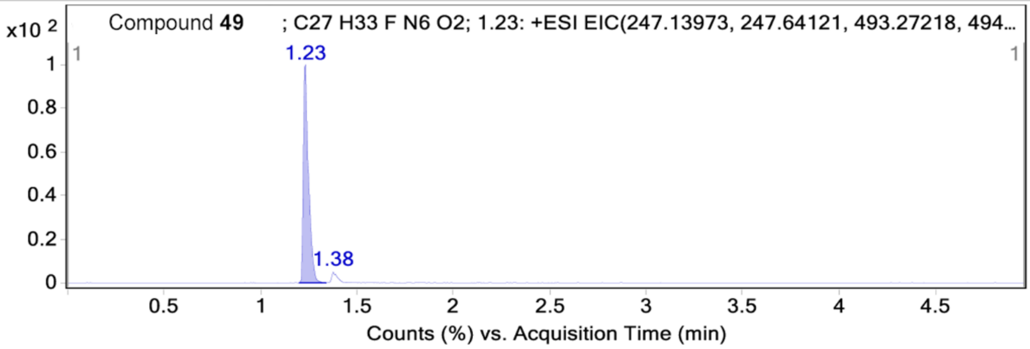

MS Zoomed Spectrum

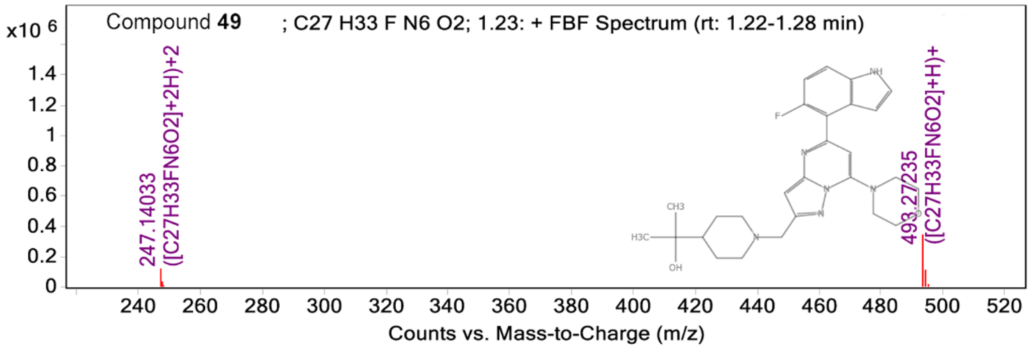

MS Zoomed Spectrum

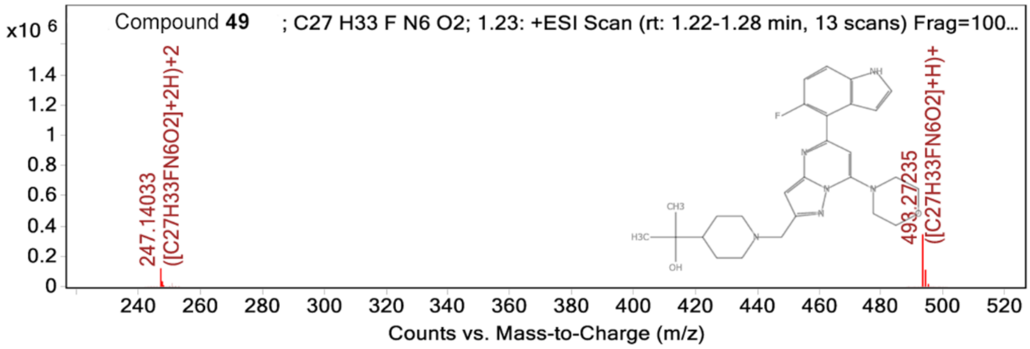

MS Spectrum Peak List

Obs. m/z	Charge	Abund	Formula	Ion/Isotope	Tgt Mass Error (ppm)
247.14033	2	115650.79	C27H33FN6O2	(M+2H)+2	
247.64167	2	39414.14	C27H33FN6O2	(M+2H)+2	
248.14313	2	6640.41	C27H33FN6O2	(M+2H)+2	
248.64393	2	647.28	C27H33FN6O2	(M+2H)+2	
493.27235	1	340789.47	C27H33FN6O2	(M+H)+	
494.27539	1	101668.37	C27H33FN6O2	(M+H)+	
495.27769	1	16018.31	C27H33FN6O2	(M+H)+	
496.28007	1	2014.64	C27H33FN6O2	(M+H)+	
247.14033	2	115650.79	C27H33FN6O2	(M+2H)+2	2.45
247.64167	2	39414.14	C27H33FN6O2	(M+2H)+2	1.86
248.14313	2	6640.41	C27H33FN6O2	(M+2H)+2	2.09
248.64393	2	647.28	C27H33FN6O2	(M+2H)+2	-0.15
493.27235	1	340789.47	C27H33FN6O2	(M+H)+	0.35
494.27539	1	101668.37	C27H33FN6O2	(M+H)+	0.51
495.27769	1	16018.31	C27H33FN6O2	(M+H)+	-0.52
496.28007	1	2014.64	C27H33FN6O2	(M+H)+	-1.18

Compound 50

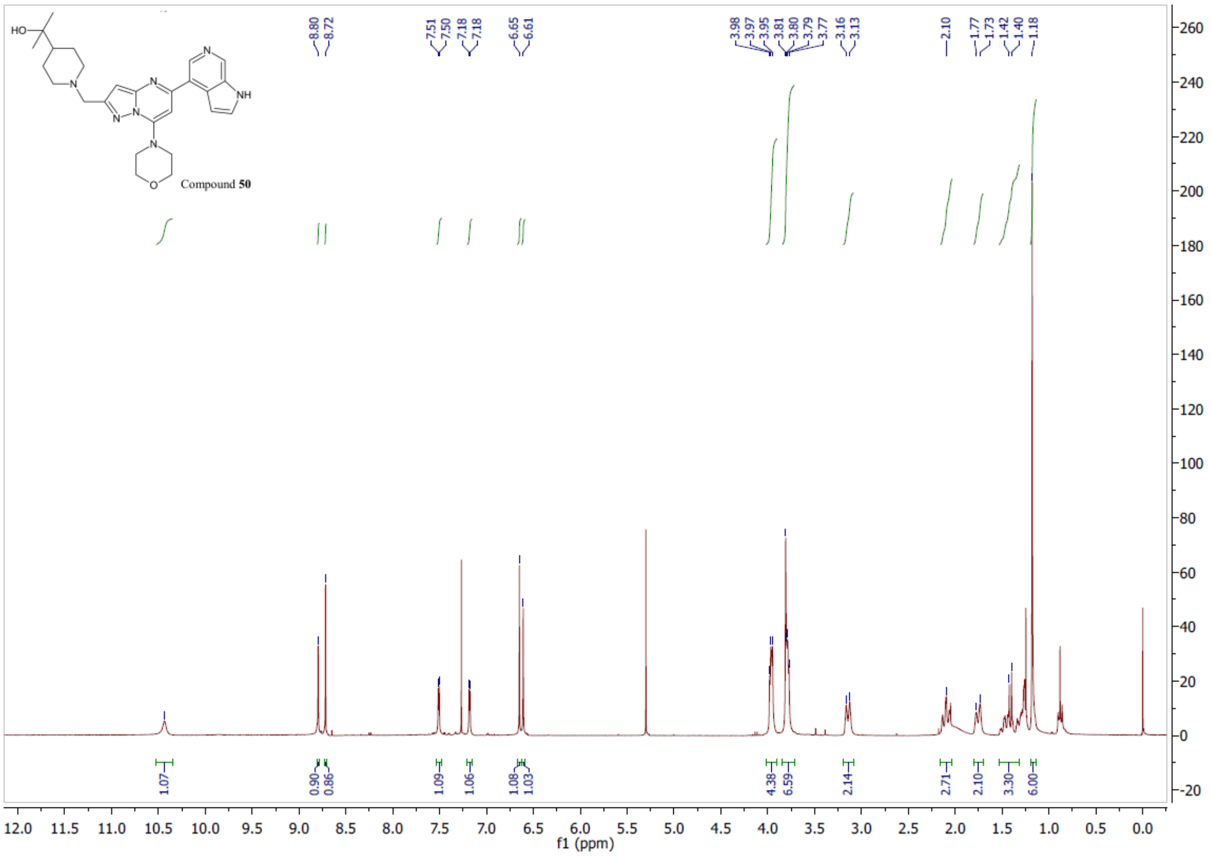

Compound 50
8.80
8.72
7.51
7.50
7.18
7.18
6.65
6.61
3.98
3.97
3.95
3.81
3.80
3.79
3.77
3.16
3.13
2.10
1.77
1.73
1.42
1.40
1.18
1.07
0.90
0.86
1.09
1.06
1.08
1.03
4.38
6.59
2.14
2.71
2.10
3.30
6.00
f1 (ppm)

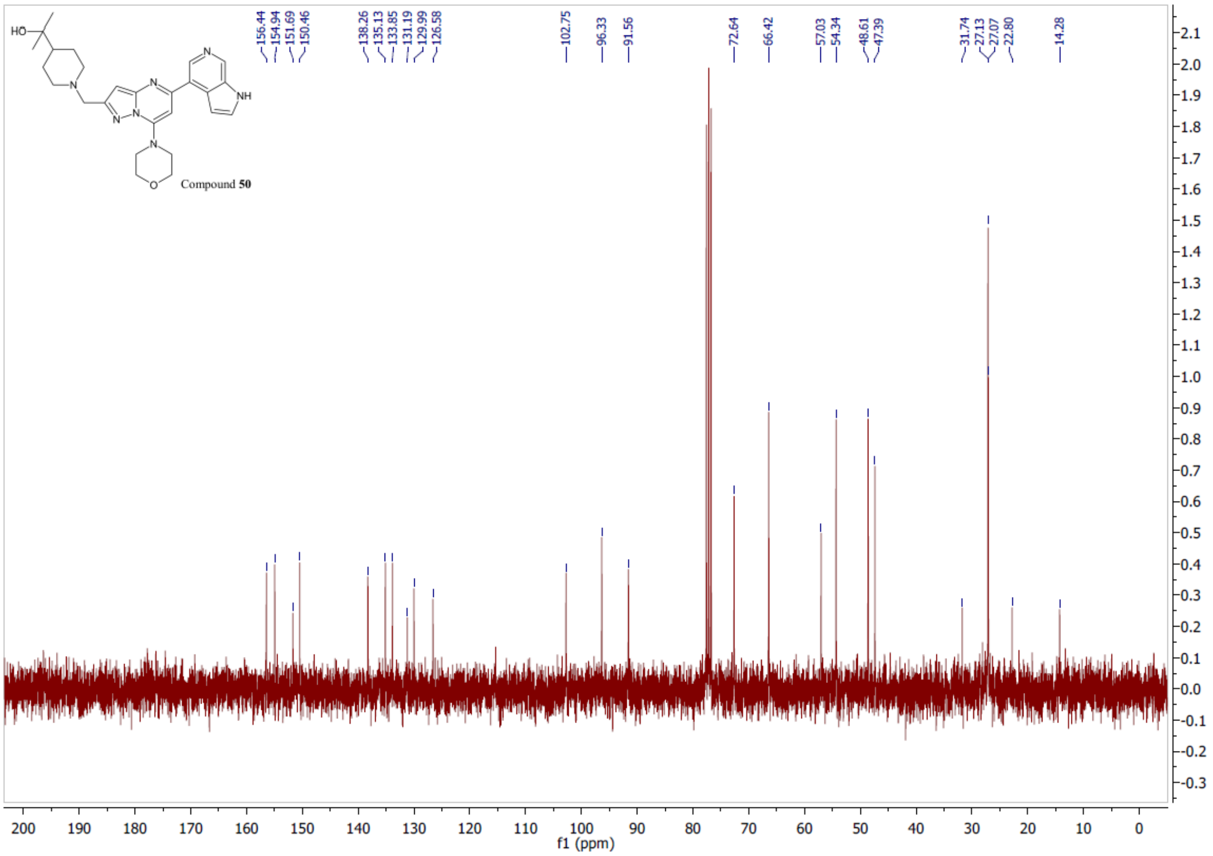

Compound 50
156.44
154.94
151.69
150.46
138.26
135.13
133.85
131.19
129.99
126.58
102.75
96.33
91.56
72.64
66.42
57.03
54.34
48.61
47.39
31.74
27.13
27.07
22.80
14.28
f1 (ppm)

Compound 50

Compound Label	Name	*m/z*	RT	Algorithm	Mass
Compound **50**	Compound **50**	476.2775	2.29	Find by Formula	475.27003

MS Zoomed Spectrum

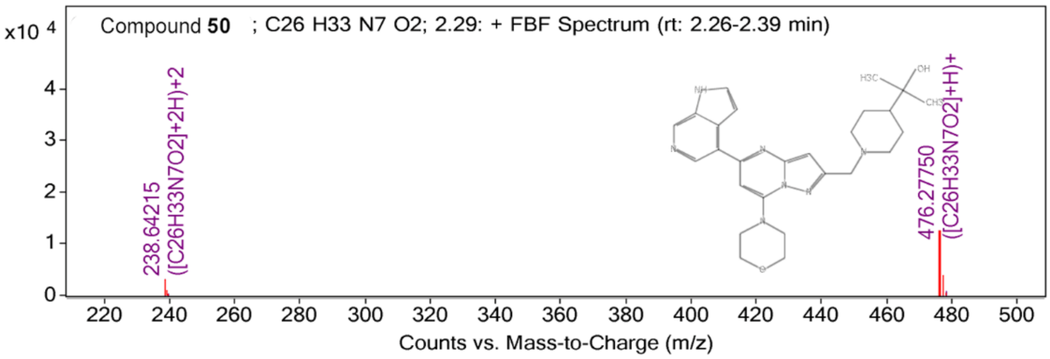

MS Zoomed Spectrum

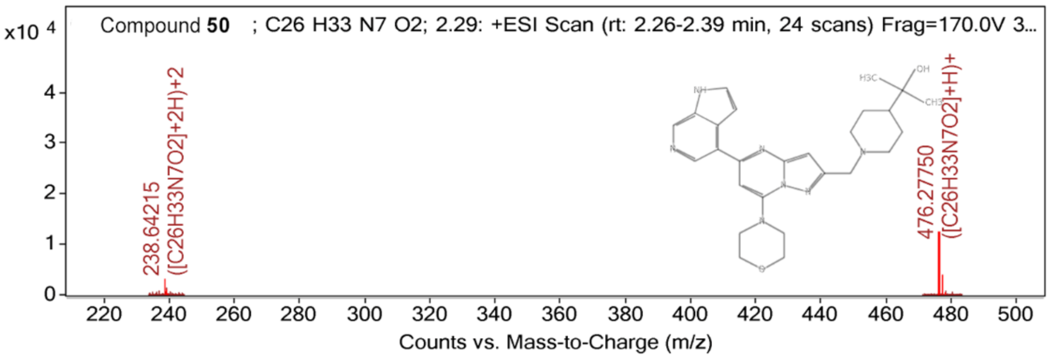

MS Spectrum Peak List

m/z	*Calc m/z*	Diff(ppm)	z	Abund	Formula	Ion
238.64215	238.64206	0.37	2	3046.57	C26H33N7O2	(M+2H)+2
239.6443	239.64487	-2.37	2	280.47	C26H33N7O2	(M+2H)+2
476.2775	476.27685	1.37	1	12351.18	C26H33N7O2	(M+H)+
477.28005	477.27972	0.68	1	3493.83	C26H33N7O2	(M+H)+
478.28194	478.28246	-1.1	1	760.19	C26H33N7O2	(M+H)+

--- End Of Report ---

Compound 51

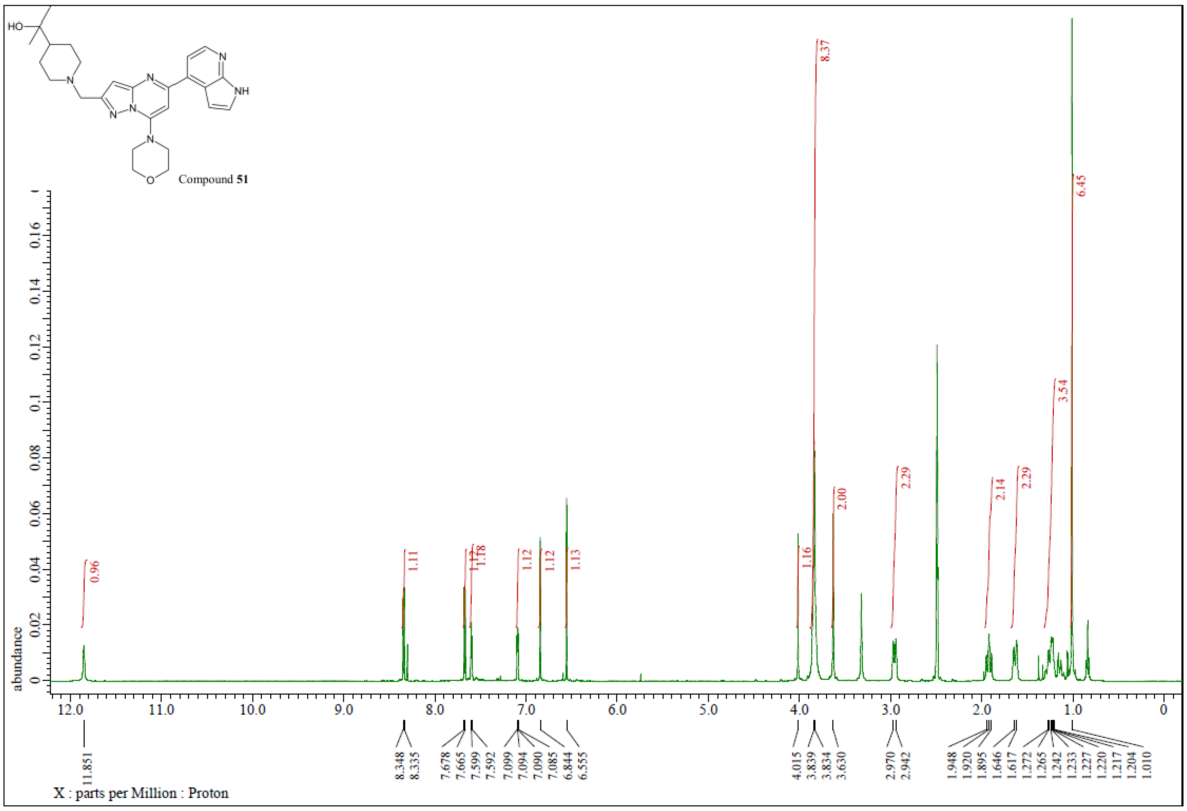
HO
N
N
N
N
NH
N
N
O
Compound 51
abundance
X : parts per Million : Proton
0.96
1.11
1.17
1.18
1.12
1.12
1.13
1.16
8.37
2.00
2.29
2.14
2.29
3.54
6.45
11.851
8.348
8.335
7.678
7.665
7.599
7.592
7.099
7.094
7.090
7.085
6.844
6.555
4.015
3.839
3.834
3.630
2.970
2.942
1.948
1.920
1.895
1.646
1.617
1.272
1.265
1.242
1.233
1.227
1.220
1.217
1.204
1.010

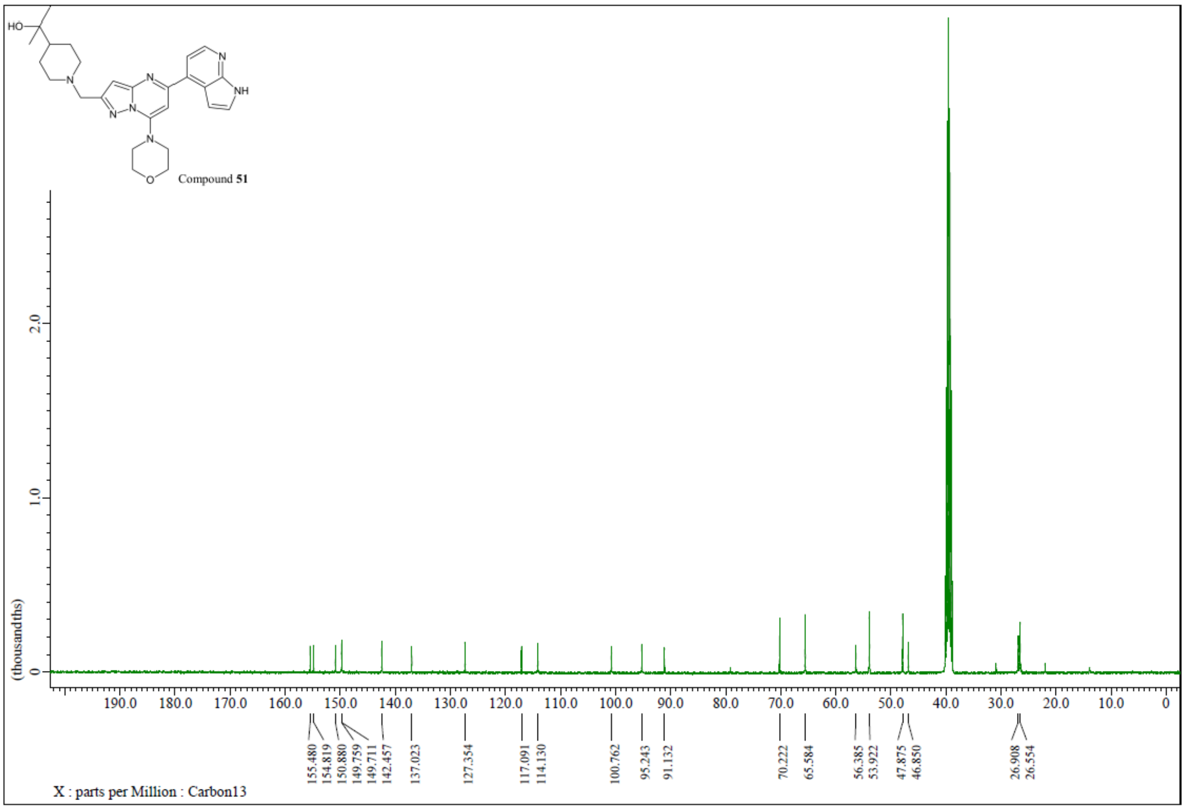
HO
N
N
N
N
NH
N
N
O
Compound 51
(thousandths)
X : parts per Million : Carbon13
155.480
154.819
150.880
149.759
149.711
142.457
137.023
127.354
117.091
114.130
100.762
95.243
91.132
70.222
65.584
56.385
53.922
47.875
46.850
26.908
26.554

Compound 51

Name	*Obs. m/z*	Obs. RT	Obs. Mass	Tgt Formula	Tgt Mass	Tgt Mass Error	Find Cpds Algorith
Compound **51**	476.27758	0.99	475.27041	C26 H33 N7 O2	475.26957	1.76	Find by Formula

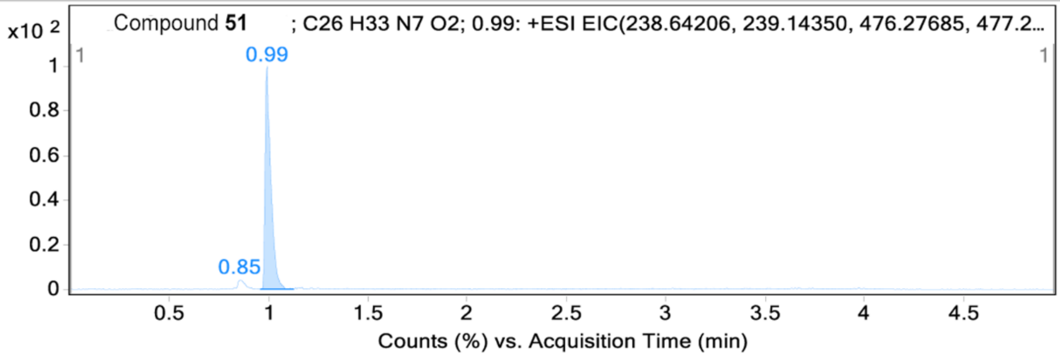

MS Zoomed Spectrum

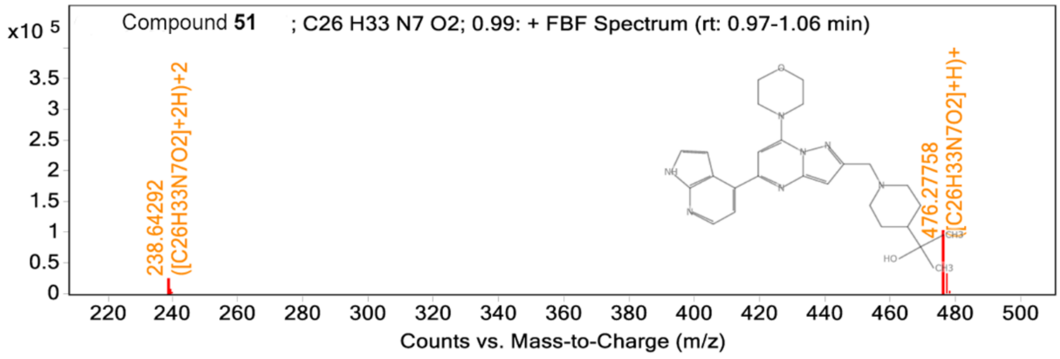

MS Zoomed Spectrum

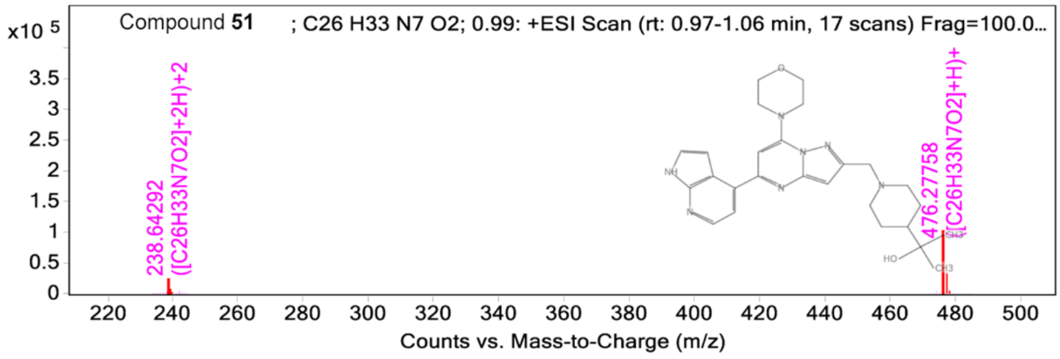

MS Spectrum Peak List

Obs. m/z	Charge	Abund	Formula	Ion/Isotope	Tgt Mass Error (ppm)
238.64292	2	23357.19	C26H33N7O2	(M+2H)+2	
239.14466	2	7984.02	C26H33N7O2	(M+2H)+2	
239.64525	2	1242.2	C26H33N7O2	(M+2H)+2	
476.27758	1	101934.38	C26H33N7O2	(M+H)+	
477.28003	1	32395.08	C26H33N7O2	(M+H)+	
478.28266	1	4780.62	C26H33N7O2	(M+H)+	
479.28502	1	601.29	C26H33N7O2	(M+H)+	
480.28589	1	75.69	C26H33N7O2	(M+H)+	
238.64292	2	23357.19	C26H33N7O2	(M+2H)+2	3.58
239.14466	2	7984.02	C26H33N7O2	(M+2H)+2	4.84
239.64525	2	1242.2	C26H33N7O2	(M+2H)+2	1.57
476.27758	1	101934.38	C26H33N7O2	(M+H)+	1.53
477.28003	1	32395.08	C26H33N7O2	(M+H)+	0.63
478.28266	1	4780.62	C26H33N7O2	(M+H)+	0.43
479.28502	1	601.29	C26H33N7O2	(M+H)+	-0.17
480.28589	1	75.69	C26H33N7O2	(M+H)+	-3.74

--- End Of Report ---

Compound 53

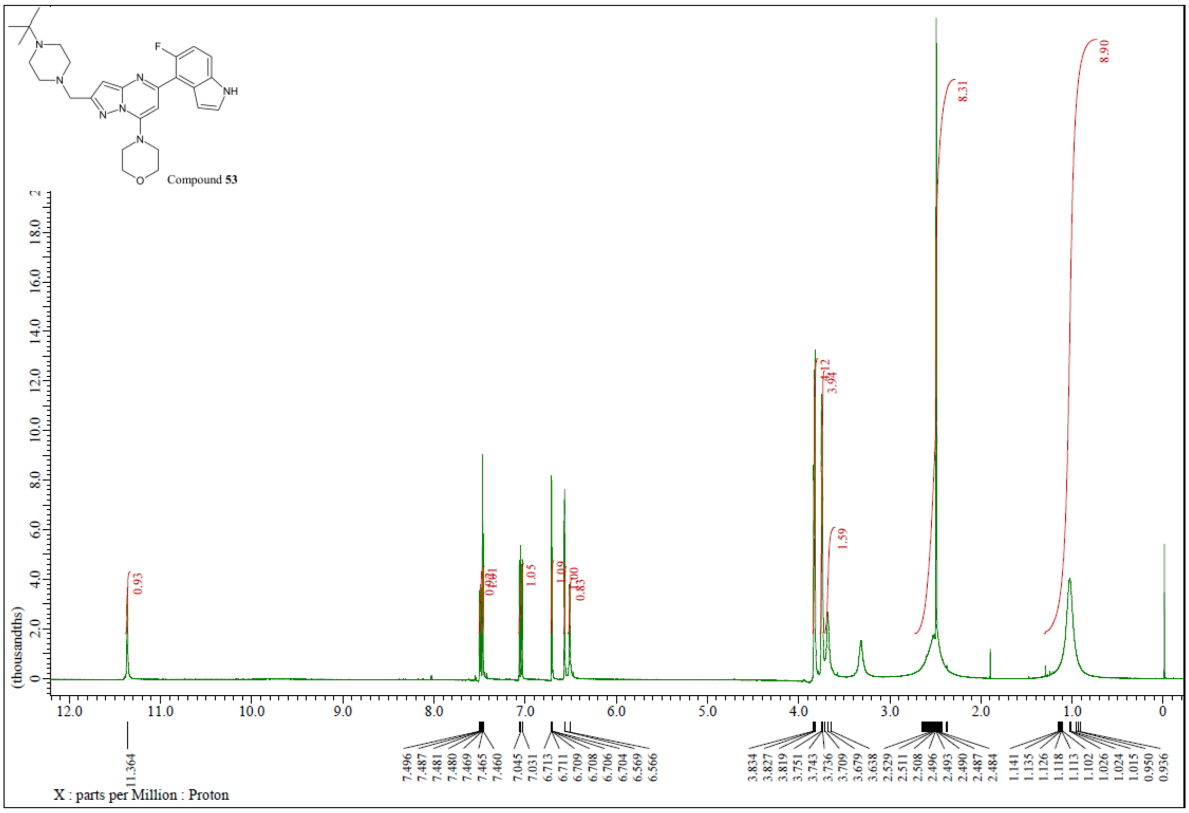
Compound 53
X : parts per Million : Proton
(thousandths)

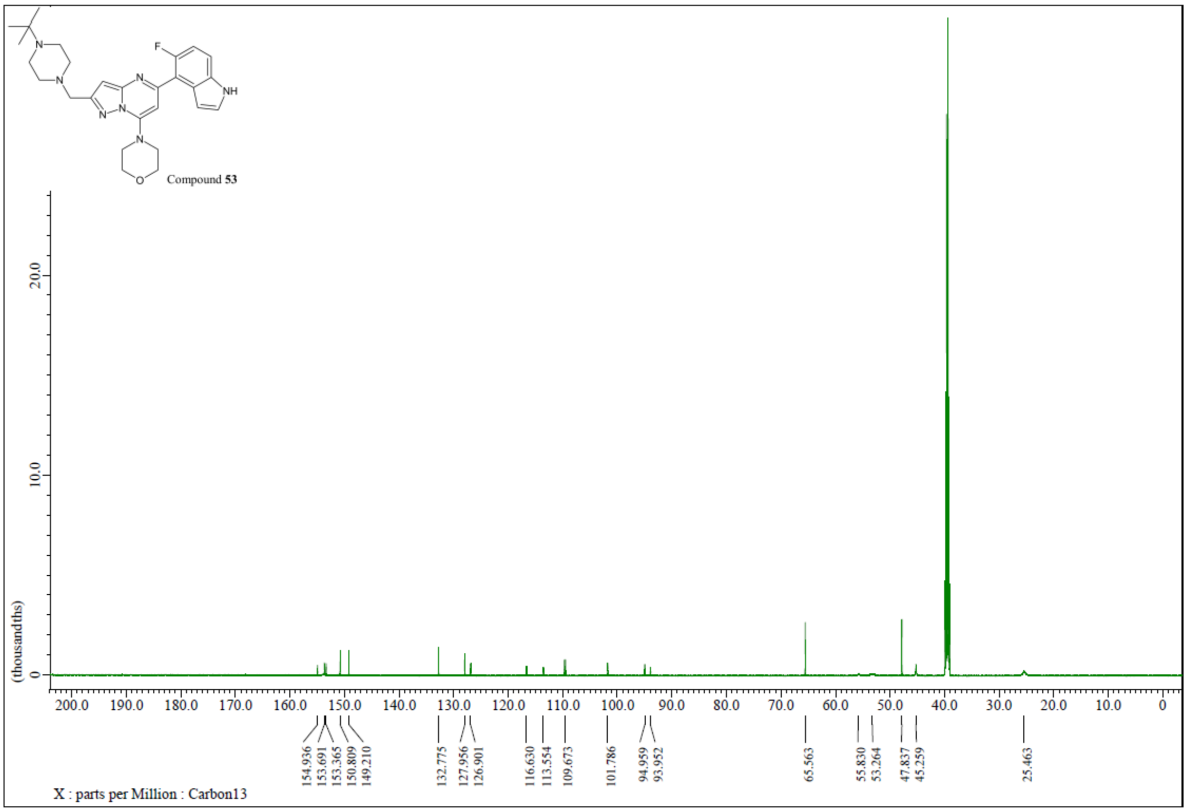
Compound 53
X : parts per Million : Carbon13
(thousandths)

Compound 53

Name	*Obs. m/z*	Obs. RT	Obs. Mass	Tgt Formula	Tgt Mass	Tgt Mass Error	Find Cpds Algorith
Compound **51**	476.27758	0.99	475.27041	C26 H33 N7 O2	475.26957	1.76	Find by Formula

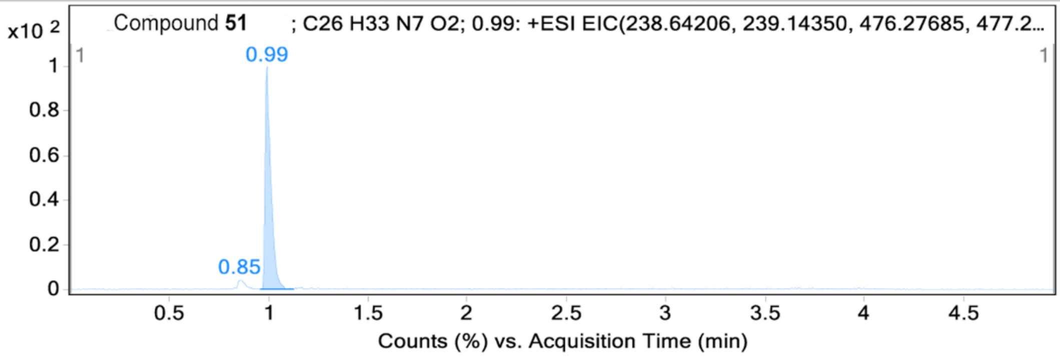

MS Zoomed Spectrum

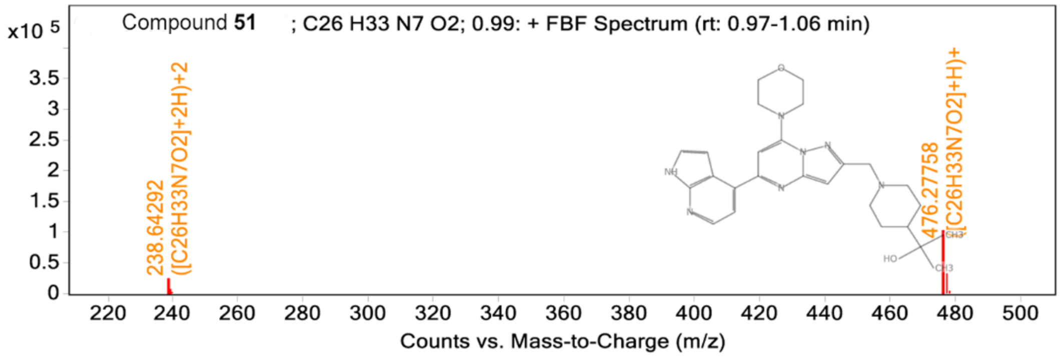

MS Zoomed Spectrum

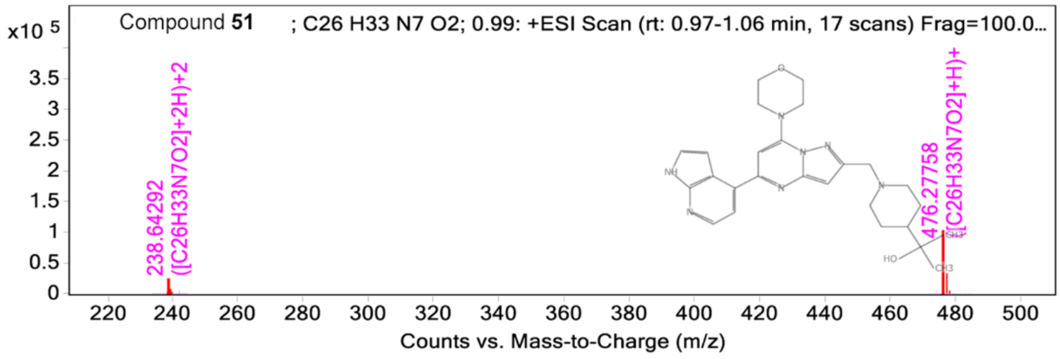

MS Spectrum Peak List

Obs. m/z	Charge	Abund	Formula	Ion/Isotope	Tgt Mass Error (ppm)
238.64292	2	23357.19	C26H33N7O2	(M+2H)+2	
239.14466	2	7984.02	C26H33N7O2	(M+2H)+2	
239.64525	2	1242.2	C26H33N7O2	(M+2H)+2	
476.27758	1	101934.38	C26H33N7O2	(M+H)+	
477.28003	1	32395.08	C26H33N7O2	(M+H)+	
478.28266	1	4780.62	C26H33N7O2	(M+H)+	
479.28502	1	601.29	C26H33N7O2	(M+H)+	
480.28589	1	75.69	C26H33N7O2	(M+H)+	
238.64292	2	23357.19	C26H33N7O2	(M+2H)+2	3.58
239.14466	2	7984.02	C26H33N7O2	(M+2H)+2	4.84
239.64525	2	1242.2	C26H33N7O2	(M+2H)+2	1.57
476.27758	1	101934.38	C26H33N7O2	(M+H)+	1.53
477.28003	1	32395.08	C26H33N7O2	(M+H)+	0.63
478.28266	1	4780.62	C26H33N7O2	(M+H)+	0.43
479.28502	1	601.29	C26H33N7O2	(M+H)+	-0.17
480.28589	1	75.69	C26H33N7O2	(M+H)+	-3.74

--- End Of Report ---

Compound 54

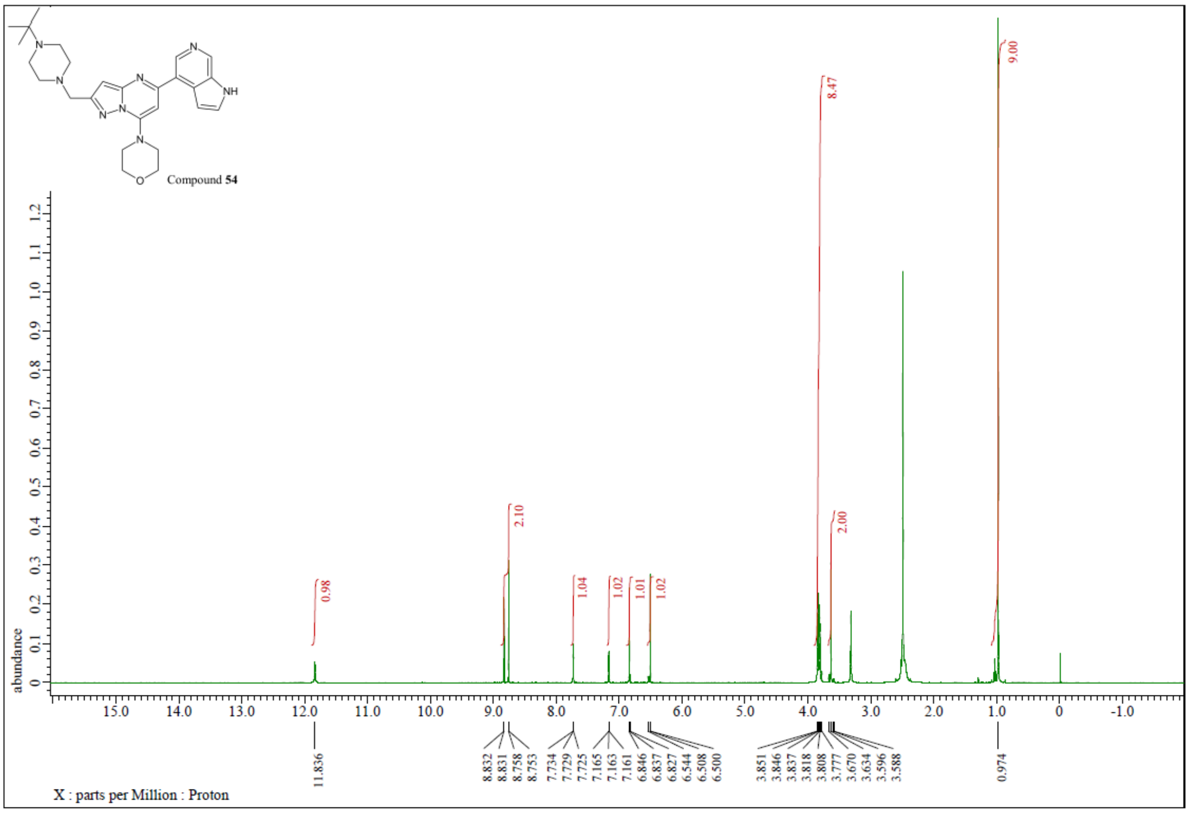

Compound 54
abundance
X : parts per Million : Proton
11.836
8.832
8.831
8.758
8.753
7.734
7.729
7.725
7.165
7.163
7.161
6.846
6.837
6.827
6.544
6.508
6.500
3.851
3.846
3.837
3.818
3.808
3.777
3.670
3.634
3.596
3.588
0.974
0.98
2.10
1.04
1.02
1.01
1.02
8.47
2.00
9.00

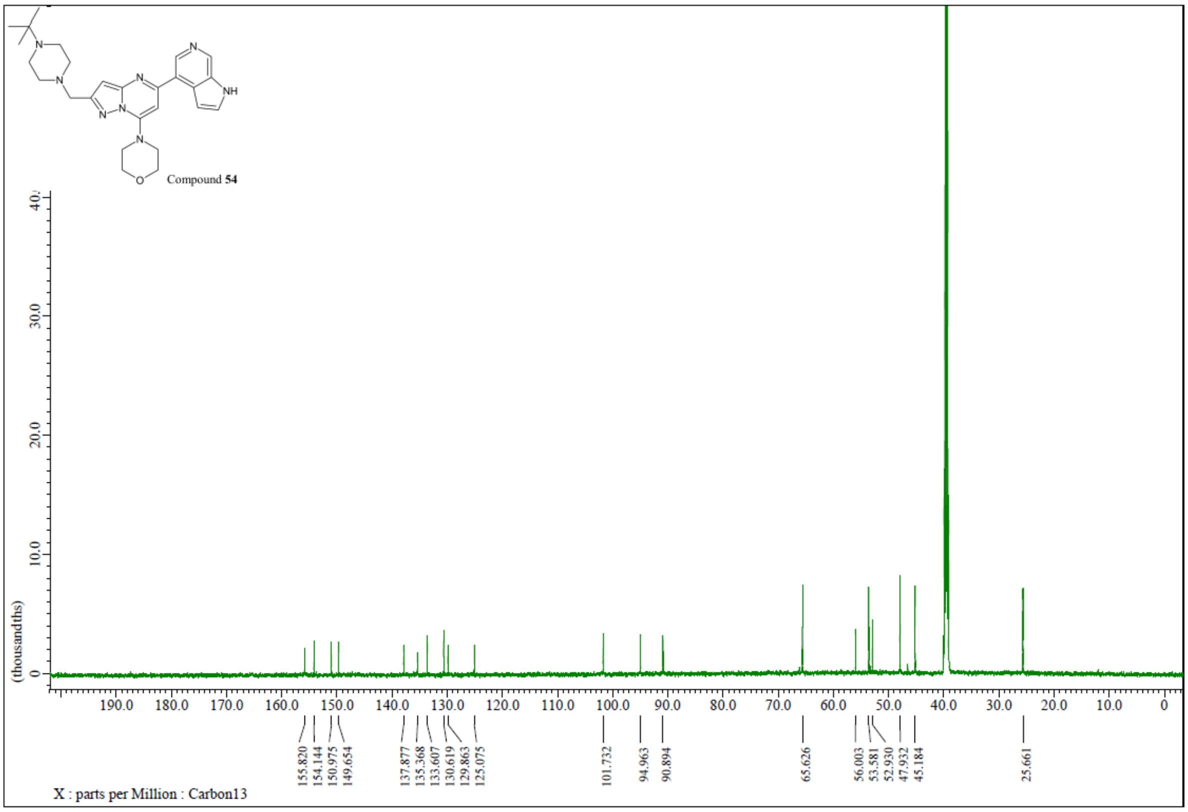

Compound 54
(thousandths)
X : parts per Million : Carbon13
155.820
154.144
150.975
149.654
137.877
135.368
133.607
130.619
129.863
125.075
101.732
94.963
90.894
65.626
56.003
53.581
52.930
47.932
45.184
25.661

Compound 54

Name	*Obs. m/z*	Obs. RT	Obs. Mass	Tgt Formula	Tgt Mass	Tgt Mass Error	Find Cpds Algorith
Compound **54**	475.29294	0.85	474.28648	C26 H34 N8 O	474.28556	1.94	Find by Formula

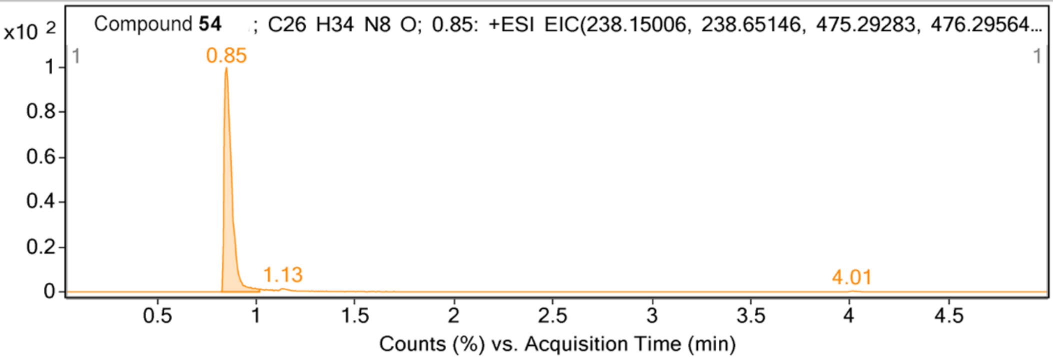

MS Zoomed Spectrum

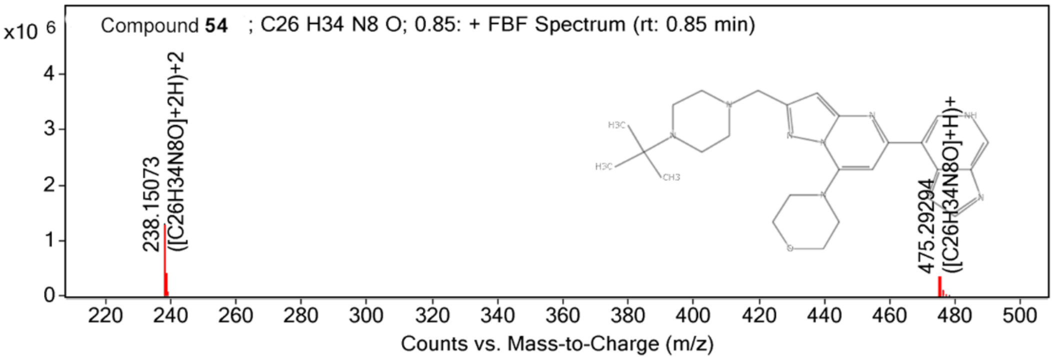

MS Zoomed Spectrum

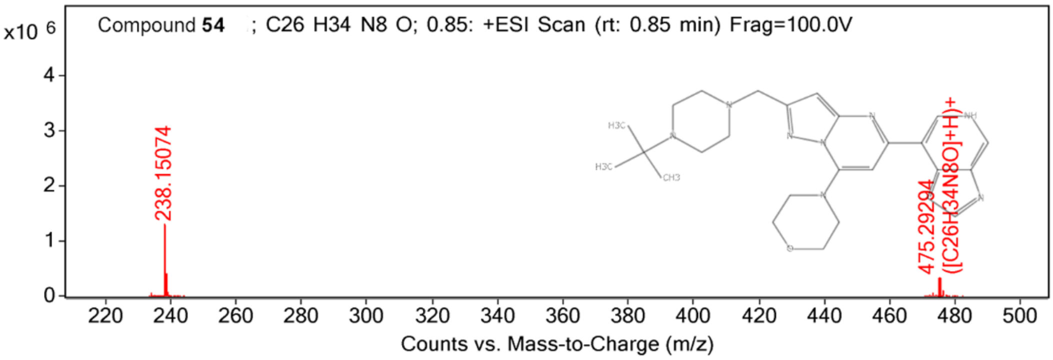

MS Spectrum Peak List

Obs. m/z	Charge	Abund	Formula	Ion/Isotope	Tgt Mass Error (ppm)
238.15073	2	1295540.25	C26H34N8O	(M+2H)+2	
238.65175	2	375450.88	C26H34N8O	(M+2H)+2	
239.15293	2	55981.86	C26H34N8O	(M+2H)+2	
475.29294	1	328461.72	C26H34N8O	(M+H)+	
476.29541	1	96053.09	C26H34N8O	(M+H)+	
477.29793	1	13882.22	C26H34N8O	(M+H)+	
478.29997	1	1075.67	C26H34N8O	(M+H)+	
238.15073	2	1295540.25	C26H34N8O	(M+2H)+2	2.85
238.15074		1302772.38			
238.65175	2	375450.88	C26H34N8O	(M+2H)+2	1.22
239.15293	2	55981.86	C26H34N8O	(M+2H)+2	0.43
475.29294	1	328461.72	C26H34N8O	(M+H)+	0.22
476.29541	1	96053.09	C26H34N8O	(M+H)+	-0.48
477.29793	1	13882.22	C26H34N8O	(M+H)+	-0.93
478.29997	1	1075.67	C26H34N8O	(M+H)+	-2.21

Compound 55

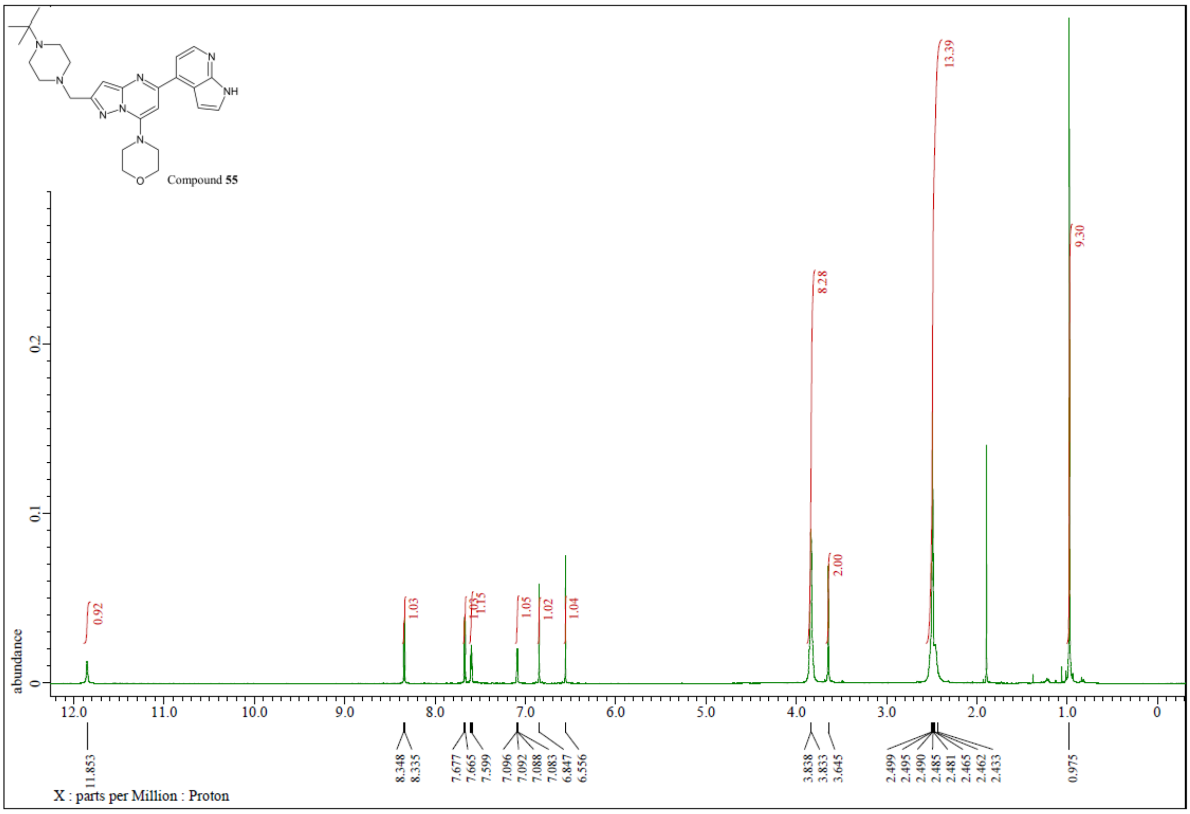
Compound 55
X : parts per Million : Proton
abundance
11.853
8.348
8.335
7.677
7.665
7.599
7.096
7.092
7.088
7.083
6.847
6.556
3.838
3.833
3.645
2.499
2.495
2.490
2.485
2.481
2.465
2.462
2.433
0.975
0.92
1.03
1.05
1.02
1.04
8.28
2.00
13.39
9.30

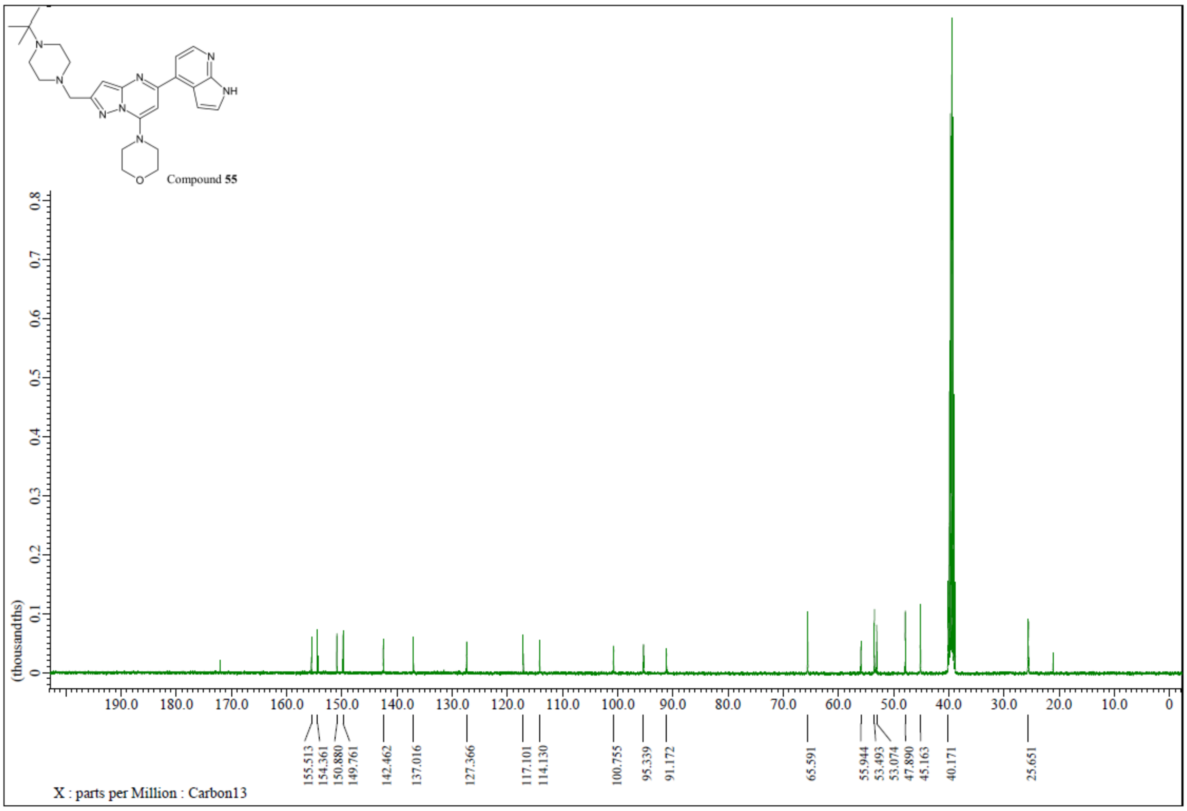
Compound 55
X : parts per Million : Carbon13
(thousandths)
155.513
154.361
150.880
149.761
142.462
137.016
127.366
117.101
114.130
100.755
95.339
91.172
65.591
55.944
53.493
53.074
47.890
45.163
40.171
25.651

Compound 55

Name	*Obs. m/z*	Obs. RT	Obs. Mass	Tgt Formula	Tgt Mass	Tgt Mass Error	Find Cpds Algorith
Compound **55**	475.2936	0.97	474.28629	C26 H34 N8 O	474.28556	1.55	Find by Formula

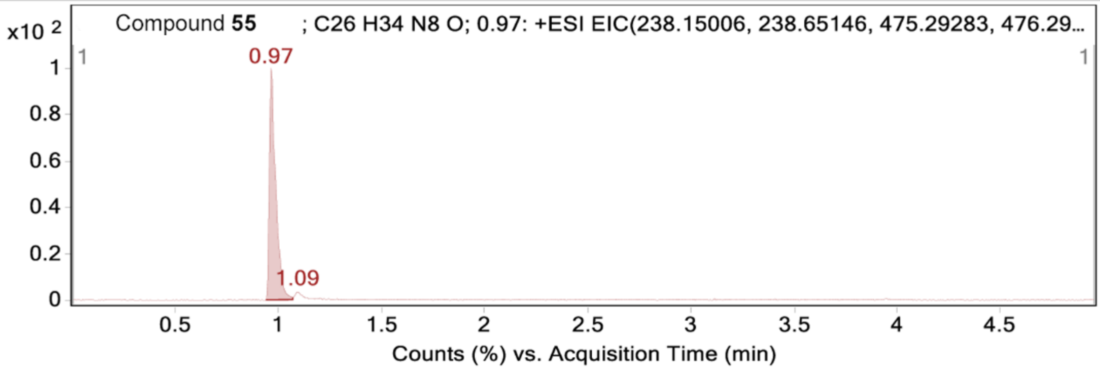

MS Zoomed Spectrum

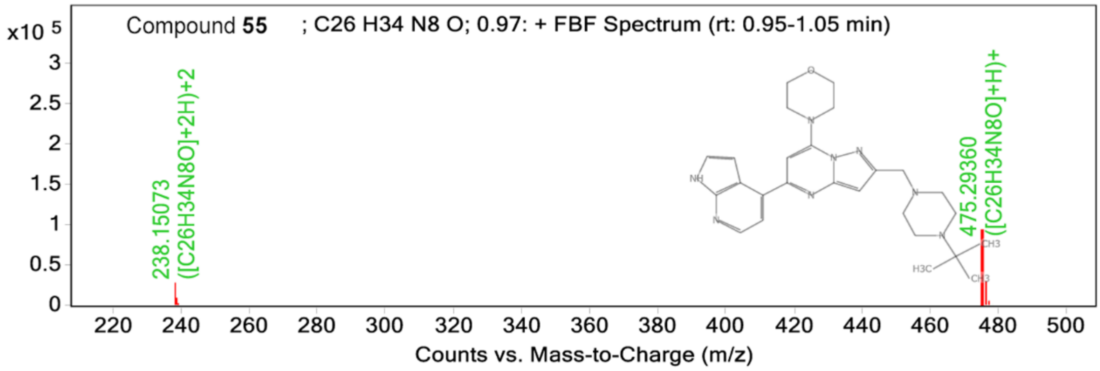

MS Zoomed Spectrum

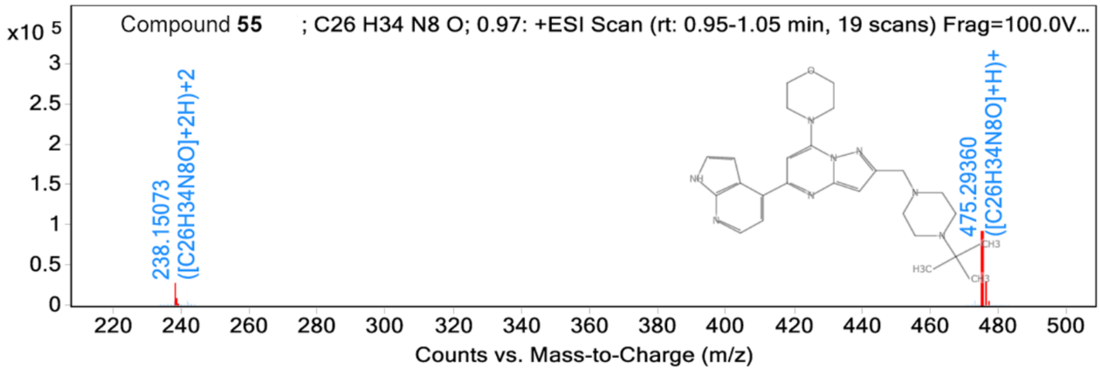

MS Spectrum Peak List

Obs. m/z	Charge	Abund	Formula	Ion/Isotope	Tgt Mass Error (ppm)
238.15073	2	27416.88	C26H34N8O	(M+2H)+2	
238.65185	2	8095.95	C26H34N8O	(M+2H)+2	
239.15226	2	1933.05	C26H34N8O	(M+2H)+2	
475.2936	1	92111.73	C26H34N8O	(M+H)+	
476.2959	1	28302.5	C26H34N8O	(M+H)+	
477.29863	1	4277.33	C26H34N8O	(M+H)+	
478.29884	1	406.24	C26H34N8O	(M+H)+	
238.15073	2	27416.88	C26H34N8O	(M+2H)+2	2.83
238.65185	2	8095.95	C26H34N8O	(M+2H)+2	1.65
239.15226	2	1933.05	C26H34N8O	(M+2H)+2	-2.38
475.2936	1	92111.73	C26H34N8O	(M+H)+	1.61
476.2959	1	28302.5	C26H34N8O	(M+H)+	0.54
477.29863	1	4277.33	C26H34N8O	(M+H)+	0.54
478.29884	1	406.24	C26H34N8O	(M+H)+	-4.58

--- End Of Report ---
